# Collaborative Optimization of Electromagnetic Interference Shielding, Adaptive Multi‐Color, and Thermal Camouflage of Basalt Fibers by Temperature‐Induced Gradient Structure Control

**DOI:** 10.1002/exp2.70135

**Published:** 2026-02-18

**Authors:** Sijie Qiao, Zhicheng Shi, Annan He, Zhiyu Huang, Aixin Tong, Binhao Wang, Jun He, Jiaxin Wang, Wei Ke, Na Yao, Shichao Zhao, Yong Qin, Weilin Xu, Fengxiang Chen

**Affiliations:** ^1^ State Key Laboratory of New Textile Materials and Advanced Processing College of Textile Science and Engineering Wuhan Textile University Wuhan Hubei China; ^2^ College of Materials Science and Engineering Qingdao University of Science and Technology Qingdao P. R. China; ^3^ Max Planck Institute of Microstructure Physics Halle Germany

**Keywords:** adaptive multicolor, annealing, basalt fiber, electromagnetic interference shielding, nickel/nickel oxide, thermal camouflage

## Abstract

Basalt fiber fabric (BFF) has gained extensive application in industrial, military, and aerospace fields due to its lightweight nature, chemical inertness, and mechanical durability. However, the inherent surface inertness and electrical insulation of BFs restrict their utilization in electromagnetic interference (EMI) shielding. In this work, we propose an innovative gradient functionalization strategy based on “plasma activation‐ALD bridging‐chemical plating‐post annealing treatments” to fabricate polychromatic BFs with exceptional EMI and thermal shielding performance. Plasma pretreatment synergizes with ALD TiO_2_ to enrich hydroxyl groups, serving as atomic‐scale “bridges” for anchoring dense Ni coatings. This process establishes interconnected conductive networks to reflect EM waves, while post annealing induces interfacial reconstruction, enhancing EMI shielding effectiveness (SE) through synergistic magnetic loss and interfacial polarization mechanisms. The optimized BFF demonstrates an outstanding EMI SE of 53.47 dB and maintains stable performance under high‐temperature and cryogenic conditions. Additionally, vivid and uniform structural colors derived from thin‐film interference were achieved on the fiber surface by modulating annealing temperatures. Notably, the high refractive index characteristics of TiO_2_, Ni, and NiO layers, coupled with their multiple refractive synergistic effects, lead to pronounced interfacial reflection of infrared radiation, which effectively reduces the radiation flux penetrating BFFs and significantly enhances overall shielding performance, underscoring their potential in thermal camouflage applications. This study establishes a groundbreaking strategy for designing multi‐color BFFs with EM and thermal shielding capabilities, and provides novel insights for developing multifunctional shielding materials while expanding BFF's application horizons in chromatic engineering and radiation protection domains.

## Graphical abstract

Polychromatic BFs with exceptional EMI and thermal shielding were fabricated based on a “plasma activation‐ALD bridging‐chemical plating‐post annealing treatments” gradient functionalization strategy. As prepared, basalt fiber/fabric demonstrates outstanding EMI SE of 53.47 dB and thermal infrared camouflage effect. Additionally, vivid and uniform structural colors derived from thin‐film interference were achieved on the fiber surface by modulating annealing temperatures.

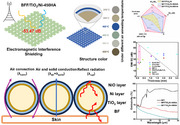

## 1 Introduction

Electromagnetic interference (EMI) originates from electromagnetic radiation signals generated by electrical loops. With the rapid advancement of wireless communication, radar technology, and the widespread use of gigahertz electrical equipment, electromagnetic pollution has reached unprecedented levels [1, 2, 3]. Research demonstrated that EMI not only disrupts the normal operation of precision electronic devices but also poses significant health risks to humans [4, 5, 6]. Particularly in the aerospace sector, high‐value precision equipment—such as satellite payloads and advanced avionics operates continuously in extremely complex service environments. These systems endure intense thermal cycling while simultaneously facing severe EMI threats and potential optical/infrared reconnaissance challenges. Traditional single‐function protective materials are inadequate to meet these dynamic integrated protection requirements. Consequently, there is an urgent need to develop efficient, lightweight, and durable EMI shielding materials suitable for aerospace, military, and other extreme environments [7, 8, 9, 10].

Basalt fibers (BFs), as a representative high‐performance inorganic fiber, possesses high tensile strength, chemical stability, corrosion resistance, thermal insulation, electrical insulation, and low dielectric constant. These properties make it an ideal low‐cost, high‐performance, and environmentally friendly alternative to carbon fibers (CFs), aramid, and ultra‐high molecular weight polyethylene fibers [11, 12, 13, 14]. However, its intrinsic properties present significant limitations in EMI SE, adaptive camouflage, and efficient thermal stealth. Consequently, multifunctional synergistic modification of BF is imperative to endow it with high‐efficiency EMI shielding capability, adaptive structural coloration, and high‐stability thermal stealth properties. Integrating these functionalities into intelligent protective skins or composite materials for external cladding of precision instruments aims to construct a triple‐functional integrated protection system capable of simultaneously evading electromagnetic detection, achieving adaptive optical camouflage, and effectively suppressing infrared thermal signature leakage. This advancement holds urgent demand and profound strategic significance for enhancing the survivability, reliability, and long‐term operational safety of aerospace precision equipment in complex electromagnetic environments and under multi‐domain reconnaissance [15, 16, 17].

Common strategies for functionalizing BF surfaces include electroless plating, chemical grafting, chemical vapor deposition, and atomic layer deposition (ALD) [18, 19]. Gao et al. successfully fabricated Ni–Co–P alloy coatings on basalt fibers via electroless plating, achieving interfacial optimization in aluminum‐based composites and significantly enhancing yield strength and tensile strength compared with unmodified counterparts [20]. In addition, Hu et al. employed ALD to construct a precisely controlled nanoscale TiO_2_/Al_2_O_3_/TiO_2_ multilayer structure on basalt fiber fabrics. By tuning the thickness of the intermediate Al_2_O_3_ layer at the nanometer scale, they achieved structural colors with varying hues, demonstrating an efficient, precisely controllable, and environmentally friendly coloration approach [21]. Despite the considerable progress in BF modification, conventional single‐step methods still suffer from inherent limitations. Electroless plating can effectively deposit conductive metals onto fiber surfaces, but the coatings often exhibit poor adhesion and nonuniform coverage due to the chemical inertness and low surface energy of BFs. In contrast, direct ALD modification ensures highly conformal film growth but typically leads to high interfacial resistance and limited electrical conductivity, since the deposited metal oxides are semiconducting or insulating in nature. Moreover, these techniques generally optimize only a single functionality (such as electrical conductivity or surface roughness), without addressing the mutual incompatibility among EMI shielding, structural color, and thermal camouflage performances. Therefore, how to fabricate a stable, uniform, and robust multifunctional coating with strong interfacial bonding on BF surfaces remains a huge challenge.

**FIGURE 1:**
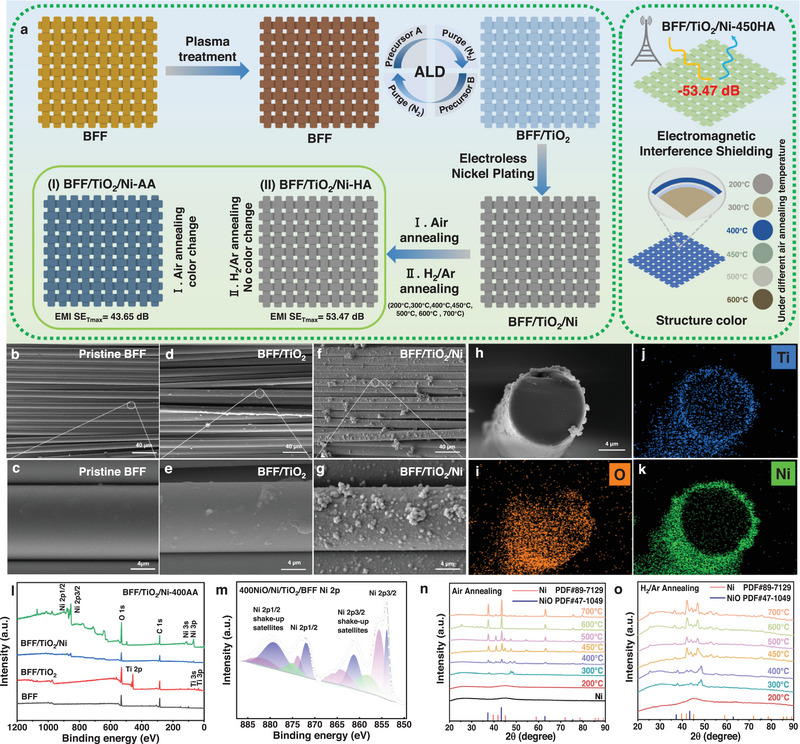
Fabrication, morphology, and structural characterizations of BFFs EMI shielding composites. (a) Schematic illustration of the preparation of polychromatic BFF/TiO_2_/Ni‐*x*AA (*x*HA) EMI shielding composites. Scanning electron microscopy (SEM) images of (b,c) pristine BFF, (d,e) BFF/TiO_2_, and (f,g) BFF/TiO_2_/Ni. (h–k) SEM image of cross‐section and corresponding element mappings of BFF/TiO_2_/Ni‐400AA showing the conformal coverage of TiO_2_, Ni, and NiO coating. (l) XPS spectrum of pristine BFF, BFF/TiO_2_, BFF/TiO_2_/Ni, and BFF/TiO_2_/Ni‐400AA. (m) XPS spectrum of Ni 2p for BFF/TiO_2_/Ni‐400AA. (n) XRD spectra of BFF/TiO_2_/Ni‐*x*AA. (o) XRD spectra of BFF/TiO_2_/Ni‐*x*HA.

In this work, we developed a novel gradient functionalization strategy based on “plasma activation‐ALD bridging‐chemical plating‐post annealing treatments,” which simultaneously enhances the hydroxyl density of the substrate surface, the interface bonding force, and the conductive continuity, achieving multi‐functional integration rather than the mere improvement of a single property, to fabricated polychromatic BFs materials with outstanding EMI shielding performance (Figure 1a). First, plasma pretreatment was utilized to introduce oxygen‐containing active groups, including hydroxyl and carboxyl, on the BF surface. These induced active groups serve as reactive sites for the subsequent ALD process, which can deposit a uniform, amorphous TiO_2_ nanocoating (≈50 nm) with high conformality (Figure S1, Supporting Information) [22, 23, 24]. The hydroxyl group (–OH) enrichment effect of the ALD TiO_2_ film provides anchoring sites for in‐situ nickel growth, creating a controllable heterogeneous “layer‐by‐layer” structure. The prepared BF material exhibits an EMI SE of 23.28 dB, demonstrating promising EMI shielding performance. Remarkably, subsequent annealing in air achieves a maximum shielding intensity of 43.65 dB, while annealing in H_2_/Ar atmospheres further enhances EMI SE to 53.47 dB, showcasing tunable EMI shielding performance. Crucially, the modified BFF maintains exceptional EMI SE stability under extreme thermal conditions, including sustained high‐temperature (150°C) and cryogenic (liquid nitrogen) environments. Furthermore, the annealed Ni layer and ALD TiO_2_, with their differences in refractive index, induce multiple reflections and refractions at the film's interfaces, generating vibrant and uniform polychromatic effects such as silver‐gray, brown, sky‐blue, and grass‐green. Interestingly, the high refractive index characteristics of TiO_2_, Ni, and NiO layers, coupled with their multiple refractive synergistic effects, lead to pronounced interfacial reflection of infrared radiation, which effectively reduces the radiation flux penetrating BFFs and significantly enhances overall shielding performance, underscoring their potential in thermal camouflage applications. “Temperature‐induced gradient structure control” promotes interface reconfiguration through thermodynamic driving forces, thereby adjusting the ratio of Ni/NiO, changing the oxygen vacancy density, and controlling the diffusion depth of TiO_2_. The resulting microstructural evolution not only enhances the adhesion of the coating but also achieves multiple functional couplings between electromagnetic shielding, optical, and thermal properties. The polychromatic BF materials prepared in this work exhibit excellent EM and thermal shielding performance and controllable multi‐color characteristics, providing new possibilities for applications of BFFs in defense, aerospace, and other fields, particularly in radar stealth, thermal camouflage, and advanced electronic equipment protection.

## 2 Experimental Section

### 2.1 Chemicals and Materials

BFFs were purchased from Hubei Huierjie Basalt Fiber Co., Ltd. The pristine BFFs used in this work is a plain‐woven fabric with an areal density of 210 g m^−2^, an average thickness of 0.12 mm, and a fiber diameter of approximately 12 µm. Prior to use, pristine BFFs were cut into 4 cm × 2.5 cm pieces and then ultrasonically rinsed with absolute ethanol and deionized water for 30 min, successively. After cleaning, the BFFs were dried in an oven at 120°C for 6 h to obtain the target fabrics required for the subsequent experiments. Titanium (IV) isopropanol (TIP, 99.999% metal‐based), sodium acetate anhydrous (CH_3_COONa) and hexadecyl trimethyl ammonium bromide (C_19_H_42_BrN, CTAB) were obtained from Shanghai Aladdin Industrial Co., Ltd. Absolute ethanol (CH_3_CH_2_OH, AR) was purchased from Sinopharm Chemical Regent Co., Ltd. High‐purity nitrogen with a purity of 99.999% was provided by Wuhan Xiangyun Gas Chemicals Co., Ltd. Sodium citrate tribasic hydrate (Na_3_C_6_H_5_O_7_·*n*H_2_O), palladium(II) chloride (PdCl_2_), tin(II) chloride (SnCl_2_), nickel chloride hexahydrate (NiCl_2_·6H_2_O), and sodium hypophosphite monohydrate (NaH_2_PO_2_·H_2_O) were obtained from Shanghai Macklin Biochemical Co., Ltd. All chemical reagents were analytically reagent and directly used without further purification. Deionized water used in this study was produced by Milli‐Q Plus 185 ultra‐pure water purification system (Millipore, Bedford, MA) with a resistivity of 18.2 MΩ·cm at 25°C.

### 2.2 Fabricated of Outstanding EMI and Adaptive Multi‐Color BFFs

#### 2.2.1 Surface Treatment of BFFs

For BFFs, the inert chemical surface makes it very difficult to chemically bond the functional components. Therefore, we attempted to enhance the surface activity by “plasma activation‐ALD bridging.” Although plasma treatment effectively introduces surface –OH and –COOH groups on BFFs, these reactive functionalities are typically superficial and thermodynamically unstable, and the plasma‐induced activation tends to decay within several hours due to surface relaxation and recombination. In contrast, the ALD TiO_2_ serves as a robust interfacial bridge that provides chemical stability, conformal coverage, and enhanced adhesion. Specifically, the ALD TiO_2_ layer exhibits permanent hydroxyl terminations at the atomic scale, ensuring long‐term reactivity for subsequent electroless nickel plating (ENP). Moreover, the conformal TiO_2_ coating offers uniform nucleation sites across the intrinsically rough BFF surface, effectively eliminating “dead zones” during metal deposition. In addition, the formation of C–O–Ti and Ti–O–Ni chemical bonds at the interface significantly strengthens interfacial mechanical integrity compared with the direct BF/Ni contact. Therefore, the introduction of the TiO_2_ bridge layer not only stabilizes the surface activation induced by plasma treatment but also establishes a durable and uniform platform for the subsequent metallization process. First, the oxygen plasma treatment was conducted in a Harrick Plasma Cleaner (PDC‐002) at 50 W, oxygen flow rate of 30 sccm (standard cubic centimeter per minute), chamber pressure of 0.8 Torr, and fiber–electrode distance of 5 cm for 5 min. Then, the ALD TiO_2_ procedure for BFFs was carried out in a closed‐chamber‐type, hot‐wall ALD reactor (D100‐4882, China) equipped with a gas‐flow system. The activated BFFs were transferred to the ALD reaction chamber and kept at 150°C for 30 min in a vacuum of ≈0.5 Torr to reach an equilibrium. High‐purity N_2_ was utilized as both the purging gas and carrier gas for both precursors at a steady flow rate of 50 sccm throughout the ALD process. TIP and H_2_O were used as the titanium source and oxygen source, respectively. TIP was kept at 80°C to produce sufficient vapor pressure in the ALD reaction chamber by carrier gas, while H_2_O was kept at the ambient temperature. The ALD TiO_2_ was carried out by alternately dosing TIP and H_2_O. The pulse, exposure, and purge time for the TIP precursor were 0.02, 8, and 25 s, and those for H_2_O were 0.1, 8, and 25 s, respectively. Thus, the duration of a complete ALD TiO_2_ cycle was 66.12 s. The estimated theoretical thickness of ALD TiO_2_ films is 0.1 nm per cycle. Therefore, the desired ALD TiO_2_ film thickness could be achieved by repeating the number of above ALD cycles. The number of ALD cycles in this study is fixed at 500 cycles.

#### 2.2.2 Synthesis of Ni‐Plated BFFs (BFF/TiO_2_/Ni)

Ni‐plated BFF was prepared via a facile electroless plating process. First, BFF was sensitized in a 50 mL 3.6 wt.% SnCl_2_/1.8 wt.% HCl aqueous solution under magnetic agitation for 20 min. The sensitized BFFs were rinsed with plenty of distilled water and dried. Second, the dried BFFs were subsequently activated in a 50 mL activation solution comprising 30 mg PdCl_2_ and 7 mL HCl at magnetic agitation for 10 min. The activated BFF was rinsed with plenty of distilled water and dried. Third, the BFF was reduced in a 50 mL 12 wt.% NaH_2_PO_2_·H_2_O aqueous solution under magnetic agitation for 10 min. The reduced BFF was rinsed with plenty of distilled water and dried. Finally, 1.4 g NiCl_2_, 1.4 g CH_3_COONa, 1.8 g NaH_2_PO_2_·H_2_O, 2.8 g C_6_H_5_Na_3_O_7_, and 2.6 mg C_19_H_42_BrN in 50 mL deionized water. The solution with a pH value of 8.2 ± 0.1 (measured using an INESA PHS‐25 pH meter) was placed in a water‐bath oscillator at 65°C. The treated BFF was immersed in the plating bath of the solution. After 30 min (as determined by subsequent experiments to be the optimal deposition time) of plating, the BFF/TiO_2_/Ni was washed with distilled water and ethanol and dried in a vacuum oven at 60°C.

#### 2.2.3 Thermal Annealing of Ni‐Plated BFFs

Further thermal annealing was conducted in air or a 10% hydrogen‐90% argon (10%H_2_‐90%Ar) atmosphere to obtain BFF/TiO_2_/Ni/NiO multilayer structures, respectively. The annealing temperatures were set at 200°C, 300°C, 400°C, 450°C, 500°C, 600°C, and 700°C, with the samples denoted as BFF/TiO_2_/Ni‐*x*AA and BFF/TiO_2_/Ni‐*x*HA, where *x* represents the annealing temperature. The annealing process featured a temperature increase/decrease rate of 2°C per minute and a holding time of 2 h.

### 2.3 Characterization

The surface morphologies and refined microcosmic structures of BFF samples were taken by the field‐emission scanning electron microscopy (FESEM, TESCAN MIRA LMS, Czech). Energy dispersive spectroscopy (EDS) mappings were performed by using the SEM. The roughness of BFF samples was measured using atomic force microscopy (AFM, Bruker Dimension Icon, Germany). The elemental states on the surface of BFF samples were tested by X‐ray photoelectron spectroscopy (XPS, Thermo Scientific K‐Alpha) under ultra‐high vacuum (2 × 10^−7^ mbar) using Al‐K*α* (*hv* = 1486.6 eV) as a radiation source. X‐ray diffraction patterns were obtained to reveal the phase composition and crystalline structure by using an X‐ray diffraction spectrometer (XRD, Bruker D8 Advance Diffractometer, Germany) at a scanning rate of 10° per minute in the 2*θ* range from 20° to 90°, equipped with a Cu‐Kα radiation source. The electrical conductivity and resistance of the materials was measured by four‐probe tester (RTS‐9, China) and Digital Multimeter (ZOYI ZT‐300AB, China), respectively. A vector network analyzer (Agilent E5071C, America) was used to test the EMI performance of the fabrics in the frequency range of 8.2–12.4 GHz (X‐band). The BFFs samples were cut to 2.5 cm × 1.5 cm in size with an average thickness of 0.13 mm. First, the samples were placed inside the mold, then the mold was placed between the two connectors (Figure S2, Supporting Information), and cables were connected separately for testing. Two scattering parameters (*S*_11_ and *S*_21_) were obtained using the waveguide method. *S*_11_ represents the electromagnetic wave received by port 1 after the electromagnetic wave emitted from port 1 is reflected by the shielding material, and *S*_21_ represents the electromagnetic wave received by port 2 after the electromagnetic wave emitted from port 1 passes through the shielding material. TE_10_ transverse wave was used as the signal source in the waveguide, in which the polarization direction of the electric field is parallel to the short edge of the waveguide. The reflection coefficient (*R*), transmission coefficient (*T*), absorption coefficient (*A*), total shielding effectiveness (SE*_T_*), reflection shielding effectiveness (SE*_R_*), and absorption shielding effectiveness (SE*_A_*) were calculated according to Equations (1)–(7) [25, 26]:

(1) $$R={\left|S_{11}\right|}^{2}$$

(2) $$T={\left|S_{21}\right|}^{2}$$

(3) A=1−R−T

(4) $$SE_{T}=-10\log\left(T\right)$$

(5) $$SE_{R}=-10\;\log\left(1-R\right)=-10\log\left(1-{\left|S_{11}\right|}^{2}\right)$$

(6) $$SE_{A}=-10\;\log\left(T/1-R\right)=-10\log\left({\left|S_{21}\right|}^{2}/1-{\left|S_{11}\right|}^{2}\right)$$

(7) $$SE_{T}=SE_{R}+SE_{A}+SE_{M}$$

Multiple reflection shielding effectiveness (SE*_M_*) is usually ignored when SE*_T_* > 15 dB [27]. For commercial uses, it is generally required that the SE*_T_* of the shielding material is > 20 dB; that is, it can attenuate more than 99% of the electromagnetic wave [28].

To demonstrate the cell phone signal blocking ability of the experimental sample in practical applications, optical photographs and videos of modified BFFs for cell phone signal shielding were taken with a cell phone camera (iPhone 14). In addition, to prove that the samples still have considerable EMI SE in extreme temperature environments, the BFF/TiO_2_/Ni‐400AA samples were subjected to a muffle furnace at 150°C for 24 h and liquid nitrogen for 2 h, respectively.

Colors after thermal annealing were observed with a 3D microscope (Olympus BX53, China). The corresponding reflective spectra and angle‐resolved reflective spectra of the colored BFFs were also collected by PG2000 Pro spectrometer (Idea Optics Co., Ltd., China) and angle resolved micro‐spectroscopy system (ARM160, China), respectively.

The mid‐infrared emissivity (*ε*) of pristine BFF and annealing BFFs were characterized using a Fourier transform infrared spectrometer (Nicolet IS50, America) equipped with an integrating sphere. The test range was 4000–400 cm^−1^ (2.5–25 µm). The surface temperature and infrared images of BFFs were measured using a thermocouple and captured with an infrared thermal imaging camera (FLIR E8), respectively.

## 3 Results and Discussion

### 3.1 Appearance and Morphology

Figure 1b–g presents SEM micrographs of the pristine BFF, BFF/TiO_2_, BFF/TiO_2_/Ni, and BFF/TiO_2_/Ni‐400AA EMI shielding composites. Individual fibers exhibit a relatively regular cylindrical morphology with a diameter of approximately 12 µm. Pristine BFF displays a smooth surface texture (Figure 1b,c). In contrast, BFF coated with TiO_2_ nanoparticles shows a small number of particle clusters on its surface (Figure 1d,e). The ALD TiO_2_ coating is uniformly distributed and conformal (Figure S3, Supporting Information). The functional groups on the TiO_2_ surface enhance its activity and improve the interfacial adhesion of the Ni layer deposited by ENP, remaining intact after annealing (Figure S4, Supporting Information). Larger particles are visible in Figure 1f,g, and combined with the corresponding O, Ti, and Ni elemental mapping images and surface elemental composition table (Figure S5, Supporting Information and Table S1, Supporting Information), confirm successful Ni deposition. Figure 1h shows a cross‐sectional SEM image of BFF/TiO_2_/Ni‐400AA after liquid nitrogen embrittlement. The coating is ultrathin, uniform, and does not significantly alter the fabric's inherent properties. Elemental mapping images of the cross‐section (Figure 1i–k) reveal uniform distributions of O, Ti, and Ni, with no apparent grooves or gaps at the interfaces. This confirms strong interfacial adhesion resulting from the self‐limiting chemical bonding reaction, further supported by SEM imaging (Figure S6, Supporting Information) and elemental mapping (Figure S4, Supporting Information). Figure S7, Supporting Information, presents AFM images of pristine BFF, BFF/TiO_2_, BFF/TiO_2_/Ni, and BFF/TiO_2_/Ni‐400AA. With the superposition of ALD TiO_2_, Ni, and NiO layers, the fabric's surface thickness and roughness gradually increased. This enhanced roughness is beneficial for improving the bonding strength between the substrate and resin in subsequent composite material preparation, which is significant for BFF applications.

### 3.2 Surface Structure and Chemical Composition

XPS was employed to identify the electronic states of the elements to obtain fundamental insights into the chemical interaction of ALD TiO_2_, Ni, and NiO, as shown in Figure 1l,m. In the XPS full spectrum, C and O are mainly present in the pristine BFF, whereas after the ALD treatment, the content of O increases, which is mainly because the pristine BFF is coated with TiO_2_ coating. Quantitative chemical analysis of Ni‐containing materials is challenging due to the complex Ni 2p peak shapes caused by multiplet splitting, shake‐up, and plasmon loss structures [29, 30]. The Ni 2p spectra of BFF/TiO_2_/Ni‐400AA are shown in Figure 1m, which consist of Ni 2p3/2 and Ni 2p1/2 peaks, which can be fitted into five components each using the Gaussian–Lorentzian function [31, 32]. The Ni 2p3/2 and Ni 2p1/2 peaks were detected at 853.98 and 871.98 eV, respectively, with corresponding satellite peaks at 861.18 and 879.38 eV, consistent with reported values for NiO [33]. Peaks at 853.88 eV (Ni 2p3/2) and 871.78 eV (Ni 2p1/2), along with their satellite peaks at 861.18 and 879.18 eV, correspond to Ni(OH)_2_ [34]. The peaks at 858.28 eV (Ni 2p3/2) and 875.08 eV (Ni 2p1/2) are attributed to NiOOH. In addition, two distinct peaks situated at 457.58 and 463.38 eV are attributed to Ti 2p3/2 and Ti 2p1/2, respectively (Figure S8a, Supporting Information), which agrees with the XPS spectra of Ti^4+^, indicating the presence of TiO_2_ layer on the BFF's surface [35].

To further explore the chemical interaction between metal oxide nano‐coatings and pristine BFF, the C 1s spectra (Figure S8b, Supporting Information) of BFF/TiO_2_ and O 1s spectra (Figure S8c,d, Supporting Information) of BFF/TiO_2_ and BFF/TiO_2_/Ni‐400AA were analyzed, respectively. The C 1s spectra of BFF/TiO_2_ located at 284.18, 285.68, and 287.68 eV correspond to the C─C/C─H, C─O─Ti, and O─C═O groups, respectively. Meanwhile, the O 1s spectra of BFF/TiO_2_ located at 531.7 and 532.6 eV correspond to C─O and C═O groups, respectively. In addition, the O 1s spectra of BFF/TiO_2_/Ni‐400AA located at 529.58, 530.98, and 531.28 eV correspond to Ni─O, O─Ni═O, and Ni─OH, respectively [36, 37]. These results suggested that the formation of C─O─M (M = Ti, Ni) bonds in metal oxide nanocoated BFF results from the self‐limiting reaction and nickel oxidation reaction that occurs at the interface between the metal oxide nanolayer and the BFF or Ni layer during ALD and annealing treatment, respectively.

Figure S9, Supporting Information, shows the XRD spectra of pristine BFF, BFF/TiO_2_, and BFF/TiO_2_/Ni. No characteristic diffraction peaks associated with ALD TiO_2_ were observed in BFF/TiO_2_, indicating the amorphous nature of the ALD TiO_2_ coating, which is consistent with the results previously reported [38]. Additionally, a characteristic diffraction peak at 44.5° in BFF/TiO_2_/Ni corresponds to the (111) plane of the face center cubic (FCC) phase of Ni [39]. Figure 1n,o shows the XRD patterns of modified BFF after annealing in air or H_2_/Ar atmospheres. For BFF/TiO_2_/Ni‐*x*AA (Figure 1n), peaks at 37.2°, 43.3°, 62.9°, 75.4°, and 79.4° correspond to the (111), (200), (220), (311), and (222) planes of FCC‐NiO (JCPDS No. 47–1049), consistent with literature [40]. As the annealing temperature increased, the Ni^3+^ content rose, inducing subtle lattice distortions on the material surface [37]. These distortions, attributed to the unique electronic structure of samples with abundant Ni^3+^ defects, likely enhanced the EMI shielding performance post‐annealing. Under H_2_/Ar annealing conditions, the reduced samples exhibited peaks at 39.7°, 41.8°, and 45.1° (Figure 1o), corresponding to the (100), (002), and (101) planes of hexagonal Ni (JCPDS No. 89–7129). Although these peaks partially overlap with those of FCC‐Ni, their relative intensity distribution and slight angular shift suggest the possible formation of a metastable hcp‐like Ni phase. Such a structural transformation is commonly associated with grain size refinement, lattice strain, and interfacial stress during annealing under H_2_/Ar conditions [41, 42]. Therefore, the existence of an hcp‐Ni structure is considered reasonable, consistent with the observed improvement in electrical conductivity and magnetic permeability of the annealed samples. This crystalline phase transformation in the Ni layer may also contribute to the improved EMI shielding performance (will be furthered later).

### 3.3 EMI Shielding Properties

**FIGURE 2:**
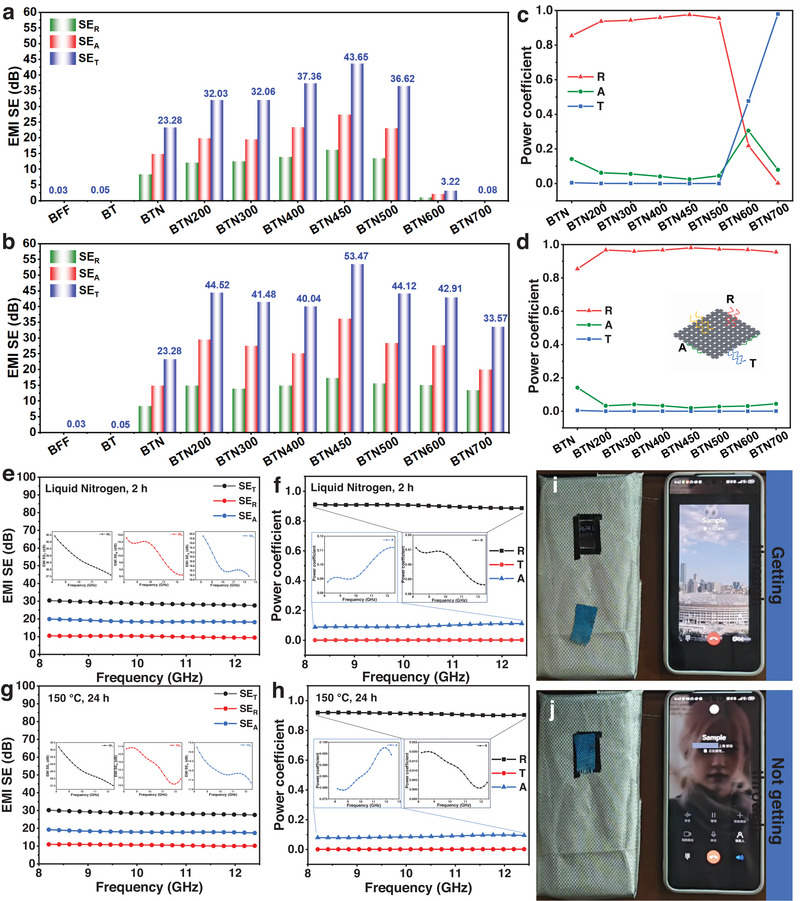
(a,b) EMI SE and (c,d) corresponding power coefficient of air annealing and H_2_/Ar annealing samples. (BFF/TiO_2_, BFF/TiO_2_/Ni, BFF/TiO_2_/Ni‐200AA, BFF/TiO_2_/Ni‐300AA, BFF/TiO_2_/Ni‐400AA, BFF/TiO_2_/Ni‐450AA, BFF/TiO_2_/Ni‐500AA, BFF/TiO_2_/Ni‐600AA, and BFF/TiO_2_/Ni‐700AA are simplified as BT, BTN, BTN200, BTN300, BTN400, BTN450, BTN500, BTN600, and BTN700, respectively.) (e–h) EMI shielding performance testing in extreme temperature environments. Realistic application simulation ((i) getting through and (j) not getting through) of the BFF/TiO_2_/Ni‐400AA for EMI shielding used in mobile phone communication.

Good electrical conductivity and outstanding stability under extreme temperature conditions lay a solid foundation for EMI shielding applications of BFFs. Figure 2 illustrates the shielding performance of BFFs after thermal annealing. As a conductive shielding material, when incident electromagnetic waves reach the fabric surface, most of them are directly reflected at the interface due to the Ni/NiO layer on the fabric surface. A vector network analyzer was used to measure the EMI shielding properties in the X‐band, which can tell each amount of the reflected, absorbed, and transmitted energy.

To further optimize the ENP process, the effect of plating duration on the EMI SE was systematically investigated. BFFs’ EMI SE subjected to different plating times (10, 20, 30, 40, 50, and 60 min) followed by H_2_/Ar annealing (*T* = 450°C) were evaluated (Figure S10, Supporting Information). The results revealed that the EMI shielding performance increased with plating time up to 30 min, reaching a peak value at this condition. Prolonging the plating beyond 30 min did not result in a significant improvement, indicating that excessive deposition contributed little to further enhancement of shielding efficiency. Therefore, 30 min was identified as the optimal plating duration, balancing both performance and process efficiency, since it not only delivered the highest shielding effectiveness but also reduced processing time compared with longer deposition periods. Accordingly, all ENP processes were conducted with a deposition time of 30 min.

Figure S11a–c corresponds to the EMI SE of pristine BFF, BFF/TiO_2_, and BFF/TiO_2_/Ni, respectively. Pristine BFF and BFF/TiO_2_ did not have EMI shielding capability (< 0.1 dB), while the EMI SE of the Ni‐coated fabric reaches 23.28 dB, demonstrating that BFF/TiO_2_/Ni exhibits certain EMI shielding properties. To further enhance EMI shielding performance and elucidate the shielding mechanism, the treated fabrics were thermally annealed in air and H_2_/Ar atmospheres to obtain modified BFFs with a “layer‐by‐layer” structure, and the EMI SE (including SE*_T_*, SE*_R_*, and SE*_A_*) of the annealed samples was tested (Figure 2a; Figure S12, Supporting Information). The results show that after thermal annealing of the fabric in an air atmosphere at 200°C, the EMI SE increases from 23.28 to 32.03 dB, attributed to the improved microstructure of the Ni coating, which becomes denser and more uniform, enhancing conductivity and reducing electromagnetic wave leakage [43]. Additionally, the formation and evolution of Ni/NiO phases strongly modulate internal multiple reflections and loss mechanisms, thereby influencing EMI shielding. In this work, the EMI SE reaches a maximum at 450°C in both atmospheres, with the H_2_/Ar‐annealed sample exhibiting the highest EMI SE (53.47 dB) and the air‐annealed sample a lower peak (43.65 dB) (Figure 2b; Figure S13, Supporting Information). This behavior is strongly correlated with the phase evolution revealed by XRD. Specifically, annealing in air leads to the formation of FCC‐NiO with a complete rock‐salt structure, whereas annealing in a reducing H_2_/Ar atmosphere stabilizes metallic Ni with a hexagonal‐like phase signature. The difference between these two crystalline phases fundamentally affects both carrier mobility and magnetic permeability [44]. This convergence at 450°C reflects an optimum balance between partial oxidation (Ni → NiO) and the preservation of conductive Ni pathways: at this temperature, the surface develops a NiO‐rich layer with relatively small grains and abundant oxygen vacancies, while residual metallic Ni domains or Ni/NiO interfacial motifs remain present [45]. Such a Ni/NiO composite structure enhances interfacial polarization, hopping/short‐range carrier transport, and magnetic‐related losses, producing a pronounced improvement in SE relative to the unmodified fabric. Moreover, annealing in an aerobic environment led to brittleness and cracking of the surface oxide coating (Figure S14, Supporting Information), interrupting the conductive network and increasing electromagnetic wave leakage. In contrast, in an H_2_/Ar atmosphere, hydrogen reacts preferentially with residual oxygen and NiO, thereby thermodynamically suppressing further oxidation and kinetically reducing existing oxides. This reductive environment maintains the integrity of the metallic Ni layer and promotes grain coarsening with fewer interfacial barriers, effectively preventing high‐temperature oxidative degradation. Consequently, H_2_/Ar‐annealed samples retain higher conductivity (3.70 × 10^4^ S m^−1^ for BFF/TiO_2_/Ni–450HA), both of which sustain Eddy‐current that preserve EMI SE above 30 dB even at 600°C–700°C. The EMI shielding performance of the modified BFFs can be precisely modulated through controlled annealing temperature, allowing it to be tailored to the requirements of specific application scenarios.

To quantitatively validate the formation and optimization of the conductive network, the electrical conductivity of BFF/TiO_2_/Ni, BFF/TiO_2_/Ni–450AA, and BFF/TiO_2_/Ni–450HA was measured using a four‐probe method. The corresponding conductivities were 7.94 × 10^3^, 1.25 × 10^4^, and 3.70 × 10^4^ S cm^−1^, respectively (Table S2, Supporting Information). In addition, the resistances measured by a digital multimeter were 8.7, 4.8, and 1.6 Ω, showing a consistent trend. The results confirm that thermal annealing significantly enhances the electrical continuity and density of the metallic Ni layer, thereby strengthening the conductive network. This improvement directly correlates with the observed increase in SE_T_, verifying that higher conductivity contributes to more efficient EM wave reflection and absorption.

Although reflection loss and absorption loss describe reflection and absorption, respectively, in Schelkunoff's theory and computational theory, neither of them represents the actual level of reflected and absorbed power [46]. When SE*_A_* is higher than SE*_R_*, this does not mean that the contribution of absorption is greater than that of reflection or that absorption is the primary shielding mechanism. Although SE*_A_* is greater than SE*_R_*, *A* is less than *R* in the range from BTN to BTN500 (Figure 2c,d). In this case, reflection is the dominant shielding mechanism rather than absorption. It is reasonable and intuitive to use the power coefficients of *R* and *A* to determine the type of shielding material and shielding mechanism.

To further clarify this issue, it is essential to distinguish the physical meanings of these parameters. The power coefficients *R* and *A* represent the energy distribution associated with a single interaction between the incident EM wave and the shielding material. Specifically, *R* denotes the fraction of incident power reflected at the surface upon first encounter, while *A* denotes the fraction of power absorbed within the material during the first transmission into the interior. They satisfy the energy conservation relationship *R* + *A* + *T* = 1, where *T* is the transmitted power fraction. In contrast, SE*_A_* is defined as the cumulative absorption loss derived from the difference between total transmission loss and reflection loss, which inherently includes corrections for multiple internal reflections.

The phenomenon in which SE*_A_* exceeds SE*_R_* while *A* remains lower than *R* arises directly from these definitional differences. When EM waves first impinge on a surface with high reflectivity, a large *R* leads to strong SE*_R_*, while only a small fraction of the energy penetrates the interior, resulting in a relatively low single‐pass absorption coefficient. However, this strong reflection simultaneously minimizes the transmitted energy escaping through the opposite surface. Under such conditions, multiple internal reflections become significant within the shielding body. The portion of energy not absorbed during the first pass undergoes repeated reflections between internal interfaces, and each reflection event contributes incremental absorption. Although the single‐pass absorption ratio (*A*) is small, the cumulative effect of multiple internal reflections leads to substantial total absorbed energy. In other words, even when *A* is low, internal reflections can amplify the total absorbed power, yielding a relatively large SE*_A_* value.

Pristine BFF and BFF/TiO_2_ exhibit high *T* (≈1), while *R* values reach 0.854 after nickel plating, indicating that Ni‐coated fabric primarily relies on reflection for EMI shielding (Figure S15, Supporting Information). As shown in Figure 2c and Figure S16, Supporting Information, the primary shielding mechanism of the fabric remains reflection in the range of 200°C–500°C. Specifically, *R* values increase from 0.937 to 0.976, while *A* values decrease from 0.062 to 0.024, with reflection power reaching a maximum at 450°C (*R* = 0.976). Annealing temperatures exceeding 600°C exacerbate the brittleness and cracking of the surface coating, disrupting the reflective mechanism and significantly increasing electromagnetic wave transmission, leading to a sharp decline in EMI SE. However, annealing in an H_2_/Ar atmosphere avoids damage to the coating and fabric toughness caused by oxygen, preserving the original properties of the fabric. Within the annealing temperature range of 200°C–700°C, the reflection power remains high (Figure 2d; Figure S17, Supporting Information). Specifically, *R* values increase from 0.967 to 0.980, while *A* values decrease from 0.033 to 0.019, reaching a maximum at 450°C (*R* = 0.981). Additionally, we tested the EMI shielding performance of the fabric under extreme environmental temperatures (Figure 2e–h). After immersing BFF/TiO_2_/Ni‐400AA in liquid nitrogen for 2 h and removing it, the sample maintains good EMI shielding performance, with EMI SE slightly decreasing from approximately 37.36 to 30 dB. In addition, after placing the sample in an oven at 150°C for 24 h, the sample's EMI SE slightly decreases from approximately 37.36 to 30 dB, but the fabric still retains excellent EMI shielding performance.

To visually demonstrate the EMI shielding performance of the BFF/TiO_2_/Ni‐400AA material, we conducted a practical application demonstration (Figure 2i,j). We wrapped a commercial fabric with EMI shielding properties around a mobile phone and cut a small hole in the fabric to allow electromagnetic waves to leak through the hole, enabling the wrapped phone to receive a call from another phone. When the blue sample (BFF/TiO_2_/Ni‐400AA) was placed over the hole, the electromagnetic waves were effectively shielded, and the phone could not receive the signal (Movie S1, Supporting Information), indicating that almost no electromagnetic waves could pass through, consistent with the *T* value approaching zero.

**FIGURE 3:**
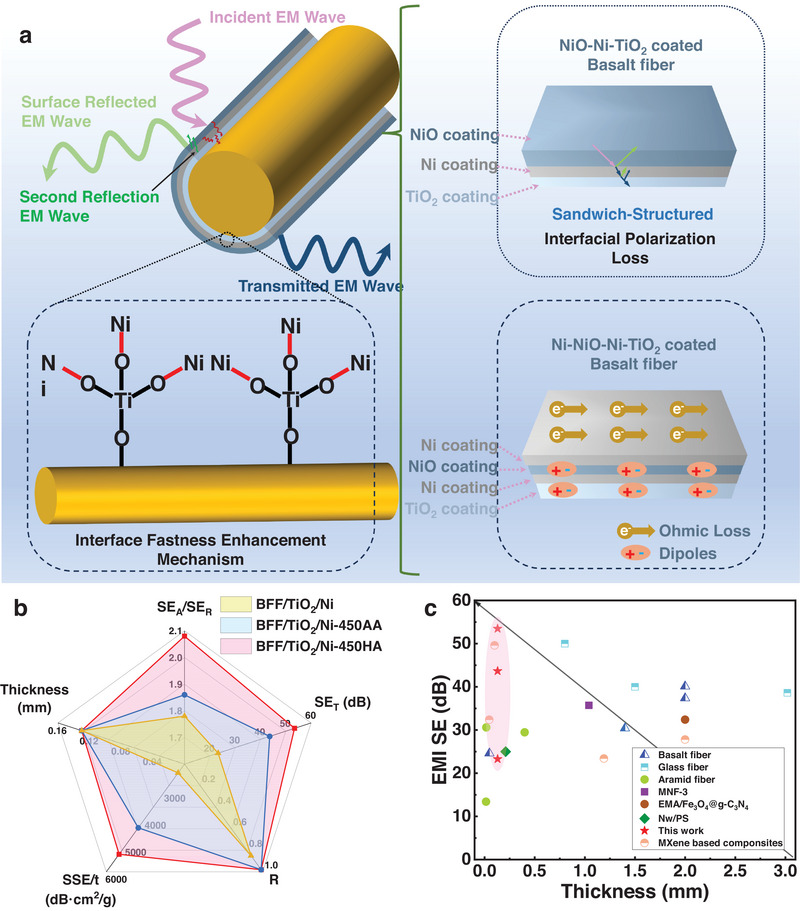
Schematic illustration of the EMI shielding mechanism and performance comparison of ultrathin materials. (a) EMI shielding mechanism schematic diagram of the modified BFFs. (b) Comparison between BFF/TiO_2_/Ni, BFF/TiO_2_/Ni‐400AA, and BFF/TiO_2_/Ni‐400HA. (c) Comparison of EMI SE and thickness of this work and previously reported some ultrathin materials with EMI shielding property.

Figure 3a illustrates the EMI shielding mechanism of BFFs with a “layer‐by‐layer” structure composed of ALD TiO_2_, Ni, and NiO layers. The outstanding EMI shielding performance can be attributed to significant reflection loss and appropriate attenuation capability. When incident electromagnetic waves reach the coating surface, the high conductivity of the Ni layer allows efficient reflection of electromagnetic waves at the interface, which is one of the main contributions to shielding performance. Subsequently, some electromagnetic waves enter the multilayer structure of NiO/Ni/TiO_2_. The conductivity and magnetism of this structure interact with high‐density electron carriers, leading to certain Ohmic losses. Ohmic loss arises from the movement of free electrons in the material under the influence of electromagnetic fields, interacting with lattice vibrations and dissipating energy. Additionally, thermal attenuation of EM wave energy, caused by this interaction, converts electromagnetic wave energy into heat. Significant dielectric losses, arising from the interaction of electromagnetic waves with polarized dipoles within the material, are also non‐negligible. Dielectric loss occurs due to the continuous reorientation of polarized dipoles in the alternating electromagnetic field, leading to energy dissipation [47]. This loss mechanism plays an indispensable role in the material, especially in the shielding of high‐frequency electromagnetic waves. Heterojunctions between layers induce dipole polarization losses and enhance the reflection and scattering of electromagnetic waves. The presence of heterojunctions causes multiple reflections and scattering of electromagnetic waves at the interfaces of different materials, increasing the propagation path of electromagnetic waves within the material and further enhancing their attenuation [48]. This multiple reflection and scattering mechanism is caused by differences in the dielectric constants between different material layers. Interestingly, the ALD TiO_2_ layer not only provides excellent chemical stability but also enhances electromagnetic wave attenuation through its dielectric properties. The high conductivity of the Ni layer ensures efficient reflection of electromagnetic waves, while the NiO layer provides additional loss mechanisms through its magnetic and dielectric properties. This rational combination addresses the challenges of BF in EMI shielding, achieving efficient EMI shielding with approximately 99.999% of incident electromagnetic waves blocked, surpassing the EMI shielding performance of many reported ultrathin materials (Figure 3b,c and Table S3, Supporting Information), making it promising for applications in defense, aerospace, and other fields.

The complex permittivity (*ε*′, *ε″*) and permeability (*μ*′, *μ*″) of BFF/TiO_2_/Ni and BFF/TiO_2_/Ni–450AA were calculated from the measured S‐parameters using the Nicolson–Ross–Weir (NRW) algorithm and transmission line theory [49, 50]. The real (*ε*′) and imaginary (*ε*″) parts of permittivity are shown in Figure S18a,b, Supporting Information. The *ε*″ component represents the energy dissipation capacity of the material, which primarily originates from interfacial polarization and conduction loss. The enhancement in *ε*′ for the annealed sample can be attributed to the formation of the continuous Ni/NiO network, which increases interfacial polarization sites and local charge displacement. The larger *ε*″ observed in BFF/TiO_2_/Ni–450AA indicates higher dielectric loss, consistent with the improved conductivity of the Ni layer after annealing.

Figure S18c,d, Supporting Information, shows the variation of the real (*μ*′) and imaginary (*μ*″) parts of the permeability as a function of frequency. Both *μ*′ and *μ*″ values for BFF/TiO_2_/Ni–450AA are enhanced compared to BFF/TiO_2_/Ni, with *μ*′ increasing from 0.9 to 1.2 and *μ*″ from 0.09 to 0.20 in the measured range. This enhancement demonstrates that post‐annealing improves the magnetic response of the composite. The increase in *μ*′ reflects higher magnetic storage capacity, whereas the rise in *μ*″ indicates strengthened magnetic loss. The improved *μ*″ is associated with multiple mechanisms, including the displacement of magnetic domain walls, enhanced ferromagnetic–antiferromagnetic exchange coupling at Ni/NiO interfaces, and the reduction of lattice strain and grain boundary pinning after annealing. These magnetic loss processes play a crucial role in the SE_A_. The enhanced *μ*″ facilitates magnetic dipole relaxation and eddy current loss, which convert incident electromagnetic energy into thermal energy.

To further evaluate magnetic loss behavior, the permeability tangent (tan *δ*_m_ = *μ*″/*μ*′) (Figure S19, Supporting Information) shows an obvious increase after annealing, confirming that magnetic dissipation becomes more pronounced in BFF/TiO_2_/Ni–450AA. The higher tan *δ*_m_ reflects stronger magnetic relaxation and eddy‐current effects, which facilitate the conversion of incident EM energy into thermal energy. The magnetic loss is responsible for the enhanced SE_A_ observed in BFF/TiO_2_/Ni–450AA, confirming that the NiO layer—through interfacial magnetic coupling and electron polarization—plays an essential role in the overall EMI shielding performance.

### 3.4 Structure Color Properties

**FIGURE 4:**
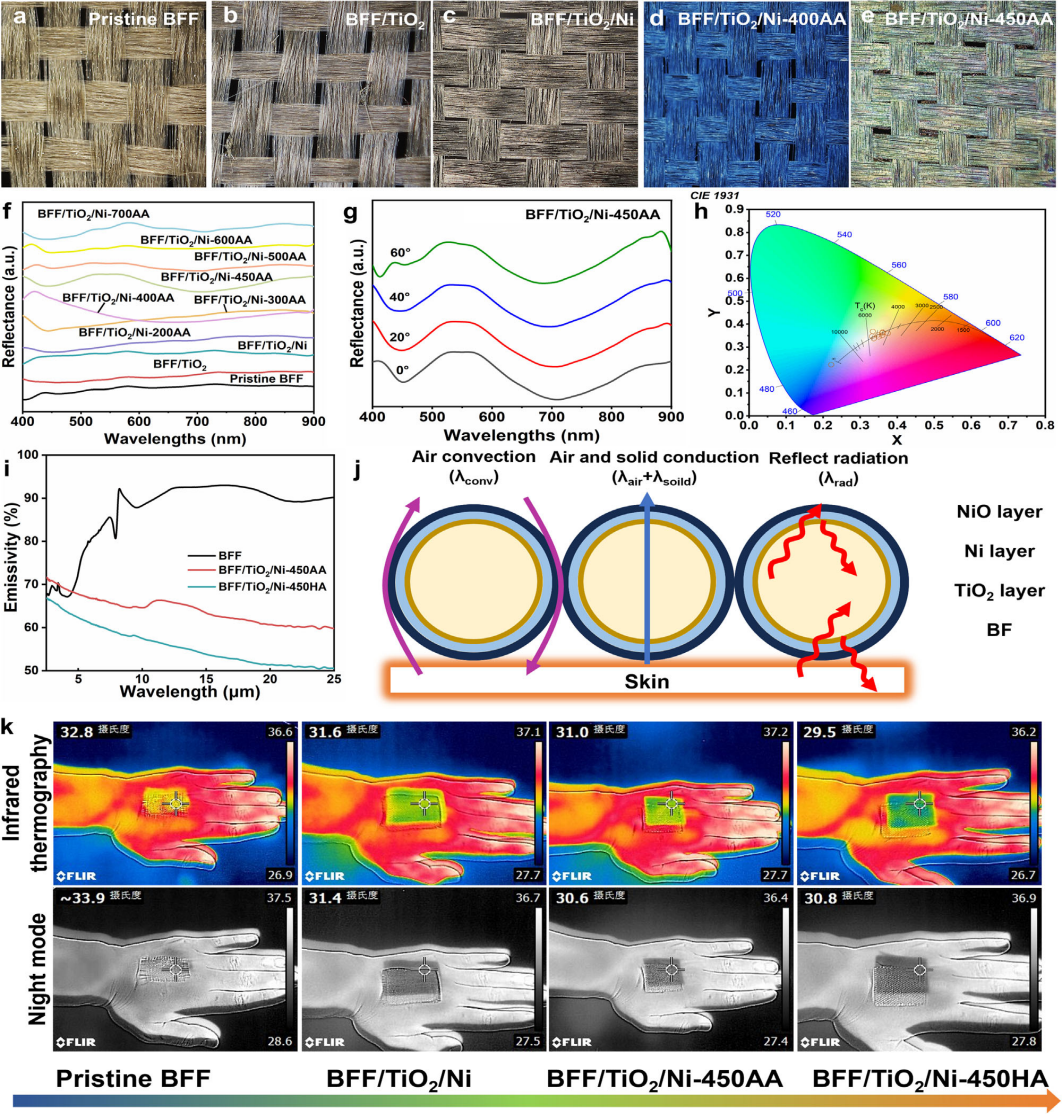
Optical properties of colored BFFs EMI shielding composites. Optical microscopy images of colorful (a) pristine BFF, (b) BFF/TiO_2_, (c) BFF/TiO_2_/Ni, (d) BFF/TiO_2_/Ni‐400AA, and (e) BFF/TiO_2_/Ni‐450AA. (f) Reflection spectra of pristine BFF, BFF/TiO_2_, BFF/TiO_2_/Ni, and BFF/TiO_2_/Ni‐*x*AA (*x* = 200, 300, 400, 450, 500, 600, and 700). (g) Different angle‐resolved reflectance spectra of BFF/TiO_2_/Ni‐450AA. (h) CIE1931 chromaticity diagram of pristine BFF, BFF/TiO_2_, BFF/TiO_2_/Ni, and BFF/TiO_2_/Ni‐*x*AA. (i) The infrared emissivity of the BFFs samples. (j) The thermal insulation mechanism of the modified BFFs sample. (k) Thermal infrared image of BFFs placed upon a hand.

After the BFF/TiO_2_/Ni samples were annealed in a series of temperatures in an air atmosphere at a rate of 2°C per minute for 2 h, the surface of the material showed bright and uniform structural colors such as silver‐gray, brown, sky blue, and grass green. To investigate the formation mechanism of the structural colors on the BFF surface after thermal annealing, we annealed the NiO layer on the BFF/TiO_2_/Ni surface through a series of temperature gradients (200°C, 300°C, 400°C, 450°C, 500°C, 600°C, and 700°C). The structural color of the fabrics was characterized using optical microscopy and reflectance spectroscopy to reveal the intrinsic mechanism. At low annealing temperatures (below 400°C), NiO may exist in an amorphous state or in a localized Ni(OH)_2_‐like transitional structure. XRD analysis confirms that with increasing annealing temperature, the surface gradually transforms into crystalline cubic NiO (rock salt structure). The cubic NiO phase with smaller grain sizes and more oxygen vacancies was formed at the surface during annealing at 400°C and was further stabilized at 600°C with a further reduction of grain boundaries [51]. Unlike long‐range periodic photonic crystals, the surface of the annealed BFFs forms a multilayer thin‐film architecture consisting of TiO_2_, Ni, and NiO layers with nanoscale thickness and refractive index differences. The vivid coloration arises primarily from thin‐film interference rather than Bragg diffraction [52, 53]. Specifically, constructive and destructive interference of incident light occurs at the multiple interfaces, governed by differences in refractive indices (*n*TiO_2 _> *n*NiO > *n*Ni) and the optical path length. This interference selectively enhances or suppresses certain wavelengths of visible light, generating angle‐insensitive structural colors observable to the naked eye. This interpretation is strongly supported by two key observations: (1) SEM and AFM images show no periodic microstructures that would be indicative of photonic crystal ordering and (2) angle‐resolved reflection spectra exhibit negligible peak shifts with increasing incidence angle, confirming the absence of iridescent behavior. As the annealing temperature increases, variations in the layer thickness, refractive index, and oxidation degree collectively modulate the optical path difference, resulting in a series of distinct color transitions from silver gray to brown, sky blue, and grass green (Figure 4a–e; Figure S20, Supporting Information). Moreover, optical microscopy observations demonstrate that the coloration remains uniform across both microscopic and macroscopic scales, confirming that the interference‐based colors are homogeneous and thermally stable.

To study the color properties of the prepared colored BFFs in more detail, the reflectance spectra of colored BFFs were recorded using an optical spectrometer (Figure 4f). In fact, visible light in nature consists of seven different colors, red, orange, yellow, green, cyan, blue, and violet, with wavelengths ranging from 400 to 760 nm. Among these colors, red, green, and blue represent the three primary colors, and the combination of two or more colors can produce a new composite color [54, 55, 56]. The spectrum of the bionic structural color BFFs prepared in this study contains multiple peaks over the entire spectrum of the incident light (Figure 4g), thus illustrating that the color of BFFs with a NiO layer deposited on the surface is not monochromatic, but a composite color.

The “iridescence effect” is a common light interference phenomenon observed in various minerals and gemstones, in which the color of an object changes depending on the angle of the observer [57]. In nature, this effect is beneficial to living organisms because it allows them to avoid natural enemies by signaling and camouflaging for their different survival needs, but it is unfriendly to textiles. Therefore, we recorded pristine BFF, BFF/TiO_2_, BFF/TiO_2_/Ni, and BFF/TiO_2_/Ni‐*x*AA at incidence angles of 0°, 20°, 40°, and 60° reflectance spectra (Figure 4g and Figure S21, Supporting Information). The minimal spectral shift is attributed to the averaging of Bragg diffraction between neighboring fibers [58] and the non‐periodic thin‐film morphology, thereby confirming the absence of iridescence in the thermally induced structural colors. It should be noted that the term “Bragg diffraction averaging” here does not refer to a true photonic crystal Bragg reflection, which requires periodic ordering at the submicron scale. Instead, it describes the macroscopic optical averaging effect arising from the random distribution and orientation of adjacent fibers, which helps to suppress angle‐dependent color shifts and contributes to the observed non‐iridescent behavior. As a result, almost no peak drift was observed in the reflection spectra, proving that the color of colored BFF prepared by thermal annealing does not have an “iridescent effect.” Finally, the CIE chromaticity values extracted from the reflectance spectra were used to quantify the color change of the colored BFF under different thermal annealing temperatures (Figure 4h).

### 3.5 Thermal Camouflage Properties

According to Wien displacement law and Stefan–Boltzmann law, the infrared radiation of an object is primarily concentrated in specific wavelength ranges related to its temperature [59, 60]. Therefore, the mid‐infrared range (3–50 µm) is the key infrared radiation band for objects at human body temperature and typical environmental temperatures. Testing the performance of modified BFFs within this range can effectively assess their infrared stealth and thermal camouflage capabilities under similar environmental conditions. To test the infrared radiation blocking performance of the modified BFFs in the mid‐infrared range for the human body, the mid‐infrared emissivity of the modified BFFs was characterized using a Fourier transform infrared spectrometer equipped with an integrating sphere, as shown in Figure 4i. Compared to the pristine BFF, the annealed samples (BFF/TiO_2_/Ni‐450AA and BFF/TiO_2_/Ni‐450HA) exhibited significantly lower emissivity in the mid‐infrared region due to the dense TiO_2_ layer and metallic Ni layer that blocks infrared radiation diffusion, with the air‐annealed sample additionally featuring a NiO layer. Notably, the emissivity curves of both BFF/TiO_2_/Ni‐450AA and BFF/TiO_2_/Ni‐450HA show minimal differences across the 2.5–25 µm wavelength range, displaying a characteristic gradual decline. It is well‐established that radiative characteristics, including reflectivity, transmittance, and emissivity in multilayer architectures, exhibit strong dependence on the incident radiation's directionality and wavelength parameters [61]. These properties are also influenced by thin‐film coatings and surface roughness characteristics. The marginal differences observed between BFF/TiO_2_/Ni‐450AA and BFF/TiO_2_/Ni‐450HA primarily originate from their comparable surface conditions resulting from similar processing parameters.

The performance enhancement of modified BFFs in reflecting and blocking mid‐infrared radiation is predominantly attributed to the high refractive indices of the TiO_2_, Ni, and NiO layers, combined with the synergistic effects of multiple refraction mechanisms. The Fresnel equations describe the interaction of light at the interface between two media with different refractive indices [62]. For light incident perpendicularly, the reflectance *R* can be expressed as:

(8) $$R={\left(\frac{n_{1}-n_{2}}{n_{1}+n_{2}}\right)}^{2}$$

where *n*_1_ and *n*_2_ represent the refractive indices of the incident medium and the refractive medium, respectively. The refractive indices are approximately 1.3 for air, 2.5 for the TiO_2_ coating, 4.0 for the Ni layer, 2.4 for the NiO coating, and 1.6 for BFFs (at mid‐IR wavelengths, *λ* ≈ 10 µm). According to Fresnel equations, theoretical calculations reveal the following reflectivity values: *R*_(BFF‐TiO2)_ ≈ 4.0%, *R*_(TiO2‐Ni)_ ≈ 6.25%, *R*_(Air‐NiO)_ ≈ 8.8%, and *R*_(Air‐Ni)_ ≈ 25.9%. Although each internal interface contributes a relatively small reflectance individually, the multi‐layer interference led to multiple partial reflections and constructive interference in the mid‐IR range, enhancing the overall total reflectivity *R*. This effectively reduces radiative flux penetration through modified BFFs, thereby significantly enhancing their overall shielding performance. Furthermore, when the incident radiation angle exceeds the critical threshold, refraction ceases entirely, triggering total internal reflection. Therefore, the thermal conductivity mechanism of modified BFFs can be described as a comprehensive effect of limiting air convection (*λ*_conv_), gas–solid thermal conduction (*λ*_air _+ *λ*_solid_), and infrared radiation (*λ*_rad_), as shown in Figure 4j.

To establish a quantitative correlation between reflectivity enhancement and emissivity suppression, the calculated *R* values were compared with the infrared emissivity data in Figure 4i. According to Kirchhoff's law of thermal radiation (*ε* + *R* + *T* = 1), the decrease in emissivity is inherently coupled with the increase in reflectivity. As shown in Figure 4i, the pristine BFF exhibits a high emissivity of approximately 0.88 at mid‐IR wavelengths (*λ* ≈ 10 µm), indicating strong infrared radiation emission and poor thermal shielding. In contrast, the emissivity of BFF/TiO_2_/Ni–450AA and BFF/TiO_2_/Ni–450HA decreases significantly to 0.65 and 0.58, respectively, consistent with the predicted increase in *R*_t_ based on interfacial optical calculations.

The synergy between enhanced reflectivity and reduced emissivity effectively suppresses both external radiative absorption and intrinsic radiative emission, resulting in a substantially lower apparent surface temperature under infrared observation. Consequently, BFF/TiO_2_/Ni–450AA and BFF/TiO_2_/Ni–450HA exhibit temperature contrasts of 4.9°C and 4.7°C relative to the human hand—significantly lower than pristine BFF (1.6°C)—confirming their superior infrared thermal camouflage capability. These results verify that the optimized multilayer optical structure modulates thermal radiation through combined interference and reflection effects, forming a dual mechanism that concurrently improves mid‐IR shielding efficiency and thermal‐infrared stealth performance.

To investigate the thermal infrared shielding performance of modified BFFs, infrared thermal imaging was performed. The thermal infrared images of modified BFFs (Figure 4k) demonstrate superior infrared radiation‐blocking capabilities. Quantitative measurements reveal the following temperature differentials: 1.6°C, 4.1°C, 4.9°C, and 4.7°C between pristine BFF, BFF/TiO_2_/Ni, BFF/TiO_2_/Ni‐450AA, BFF/TiO_2_/Ni‐450HA, and the human hand, respectively. Corresponding temperature differences between these samples and the ambient environment were measured as 5.3°C, 2.8°C, 2.0°C, and 2.2°C. Notably, BFF/TiO_2_/Ni‐450AA and BFF/TiO_2_/Ni‐450HA exhibited a color closer to the ambient environment in thermal imaging, attributable to their enhanced infrared thermal reflectance properties.

Furthermore, to further elucidate the role of the TiO_2_ interlayer in enhancing the thermal camouflage performance, infrared thermal imaging was conducted using a thermal camera (FLIR E4, American) under a controlled ambient temperature. Figure S22, Supporting Information, shows the infrared images of BFF/TiO_2_/Ni (a) and BFF/Ni (b) samples placed on a human hand. The temperature differences between the samples and the hand were measured to be 6.8°C and 5.9°C, respectively, while the corresponding temperature differences between the samples and the surrounding environment were 2.7°C and 3.6°C. Notably, the BFF/TiO_2_/Ni sample exhibited a surface color in the thermal image that was closer to that of the background environment, indicating a reduced apparent temperature contrast. This improvement can be attributed to the enhanced infrared reflection and decreased emissivity induced by the TiO_2_ interlayer, which effectively suppresses thermal radiation exchange between the sample and the environment. These results confirm that the introduction of the TiO_2_ bridge layer contributes to improved thermal stealth capability and a more stable infrared camouflage performance.

It is well known that conventional camouflage systems primarily target evasion within the visual spectrum, yet their concealment capabilities prove ineffective against unmanned aerial vehicles (UAVs) equipped with infrared thermal imaging technology. The modified BFFs developed in this study demonstrate transformative potential for thermal‐infrared stealth applications in extreme operational environments. This integrated multifunctional textile exhibits significant application value and potential in infrared camouflage domains. Providing comprehensive protection for precision equipment and personnel in aerospace and battlefield environments, the fabric substantially reduces vulnerability to attacks from UAVs with advanced infrared thermal imaging capabilities while effectively evading aerial surveillance. Critically, surface modifications through metal oxide coatings and metallic nanolayers minimally impact the substrate's performance, thereby showcasing exceptional development potential for next‐generation aerospace thermal shielding systems and adaptive battlefield concealment solutions.

## 4 Conclusion

In conclusion, we have successfully designed EMI shielding BFFs with adaptive structural colors and thermal camouflage. As‐prepared EMI shielding BFFs achieved a SE*_T_* value of 53.47 dB at an annealing temperature of 450°C, and even after exposure to liquid nitrogen and sustained high temperatures of 150°C, the SE_T_ value remained above 30 dB. In addition, when the BFF/TiO_2_/Ni samples were annealed in an air atmosphere, the fabric surface could generate vibrant structural colors with uniform hues spanning different color categories by controlling the annealing temperature. Moreover, the high refractive indices of the TiO_2_, Ni, and NiO layers enhance infrared reflection at the interface, reducing radiation flux through BFFs and improving thermal shielding. This thermodynamic‐driven effect resulting from “temperature‐induced gradient structure control” promotes interface reconfiguration, adjusts the ratio of Ni/NiO, alters the density of oxygen vacancies, and controls the diffusion depth of TiO_2_. These microscopic structural changes not only enhance the adhesion of the coating but also broaden the multifunctionality and adaptability of EMI shielding fabrics and show great potential for enhancing their intelligence and applicability in adaptive stealth fields.

## Author Contributions

**Sijie Qiao**: investigation, writing – original draft. **Zhicheng Shi**: investigation, writing – original draft. **Annan He**: writing – review and editing. **Zhiyu Huang**: writing – review and editing. **Aixin Tong**: writing – review and editing. **Binhao Wang**: writing – review and editing. **Jun He**: writing – review and editing. **Jiaxin Wang**: writing – review and editing. **Wei Ke**: writing – review and editing, visualization. **Na Yao**: writing – review and editing, visualization. **Shichao Zhao**: writing – review and editing, visualization. **Yong Qin**: supervision. **Weilin Xu**: supervision. **Fengxiang Chen**: conceptualization, methodology, visualization, supervision, writing – review and editing, funding acquisition.

## Conflicts of Interest

The authors declare no conflicts of interest.

## Supporting information




**Supporting File 1**: exp270135‐sup‐0001‐SuppMat.docx.

**Supporting File 2**: exp270135‐sup‐0002‐Movie1.mp4.

**Supporting File 3**: exp270135‐sup‐0003‐data.pptx.

## Data Availability

The data that support the findings of this study are available from the corresponding author upon reasonable request.
