# Supplementary material for: Collaborative Optimization of Electromagnetic Interference Shielding, Adaptive Multi‐Color, and Thermal Camouflage of Basalt Fibers by Temperature‐Induced Gradient Structure Control
Source: Exploration (Beijing). 2026 Feb 18;6(2):70135. doi: 10.1002/exp2.70135 (PMC13094530; doi:10.1002/exp2.70135)
Supplement: Supplementary file 1 — Supporting File 1: exp270135‐sup‐0001‐SuppMat.docx. [file EXP2-6-70135-s002.docx]

**Supporting Information**

for

**Collaborative Optimization of Electromagnetic Interference Shielding，Adaptive Multi-color, and Thermal Camouflage of Basalt Fibers by Temperature-Induced Gradient Structure Control**

Sijie Qiao^1^, Zhicheng Shi^1^, Annan He^1^, Zhiyu Huang^1^, Aixin Tong^1^, Binhao Wang^1^, Jun He^1^, Jiaxin Wang^1^, Wei Ke^1,*^, Na Yao^1,*^, Shichao Zhao^2,*^, Yong Qin^2,3^, Weilin Xu^1^, Fengxiang Chen^1,*^

^1^ State Key Laboratory of New Textile Materials and Advanced Processing, College of Textile Science and Engineering, Wuhan Textile University, Hubei, Wuhan; 430200, China

^2^ College of Materials Science and Engineering, Qingdao University of Science and Technology, Qingdao 266042, P. R. China

^3^ Max Planck Institute of Microstructure Physics, Weinberg 2, D-06120 Halle, Germany

*Corresponding author: wke@wtu.edu.cn (W. Ke); [nayao@wtu.edu.cn](mailto:%20nayao@wtu.edu.cn) (N. Yao); ucas126@126.com (S. Zhao); fxchen_czx@wtu.edu.cn (F. Chen)

**Supplementary Figures and Tables**


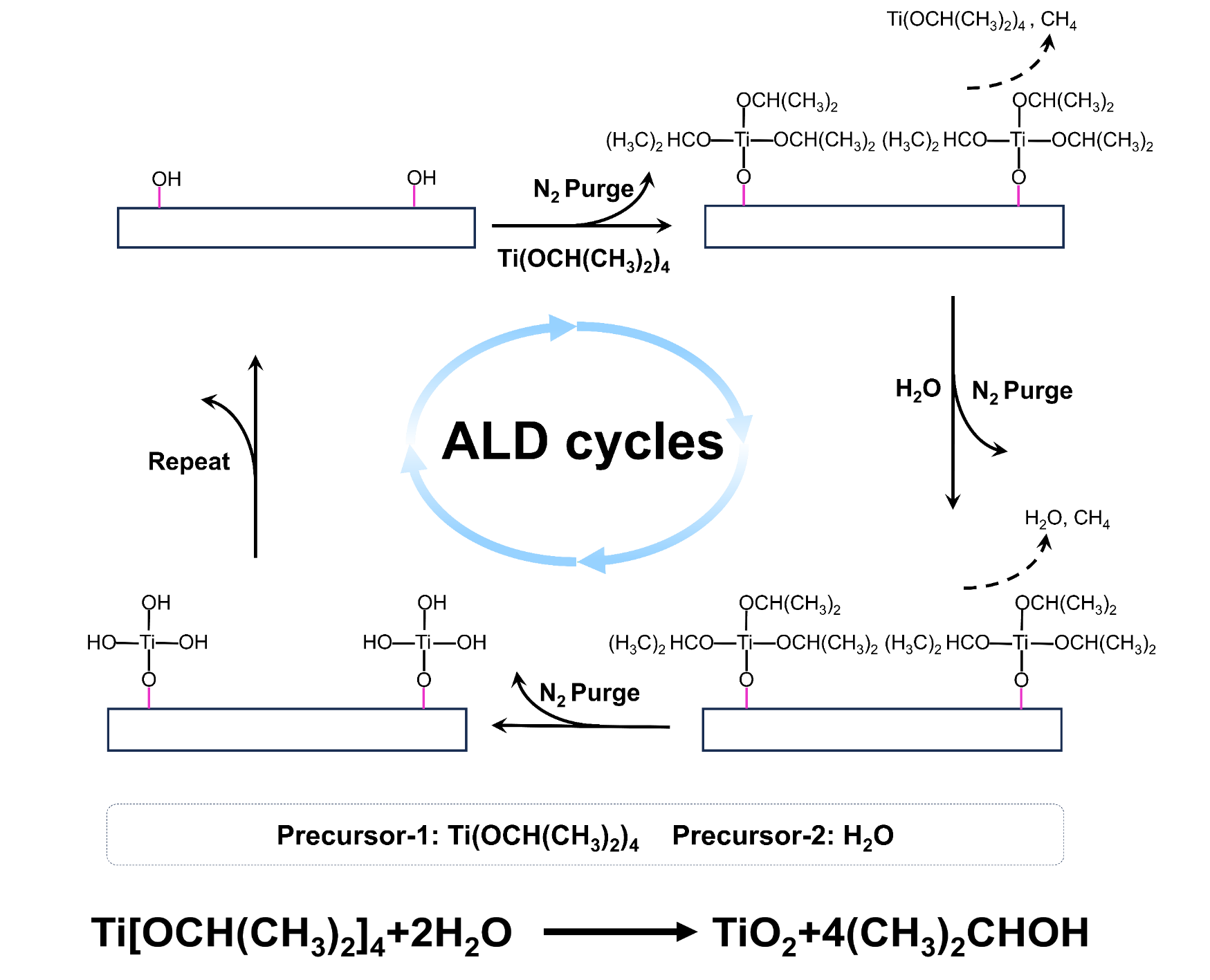


**Fig. S1** Schematic illustration of ALD TiO_2_ process.


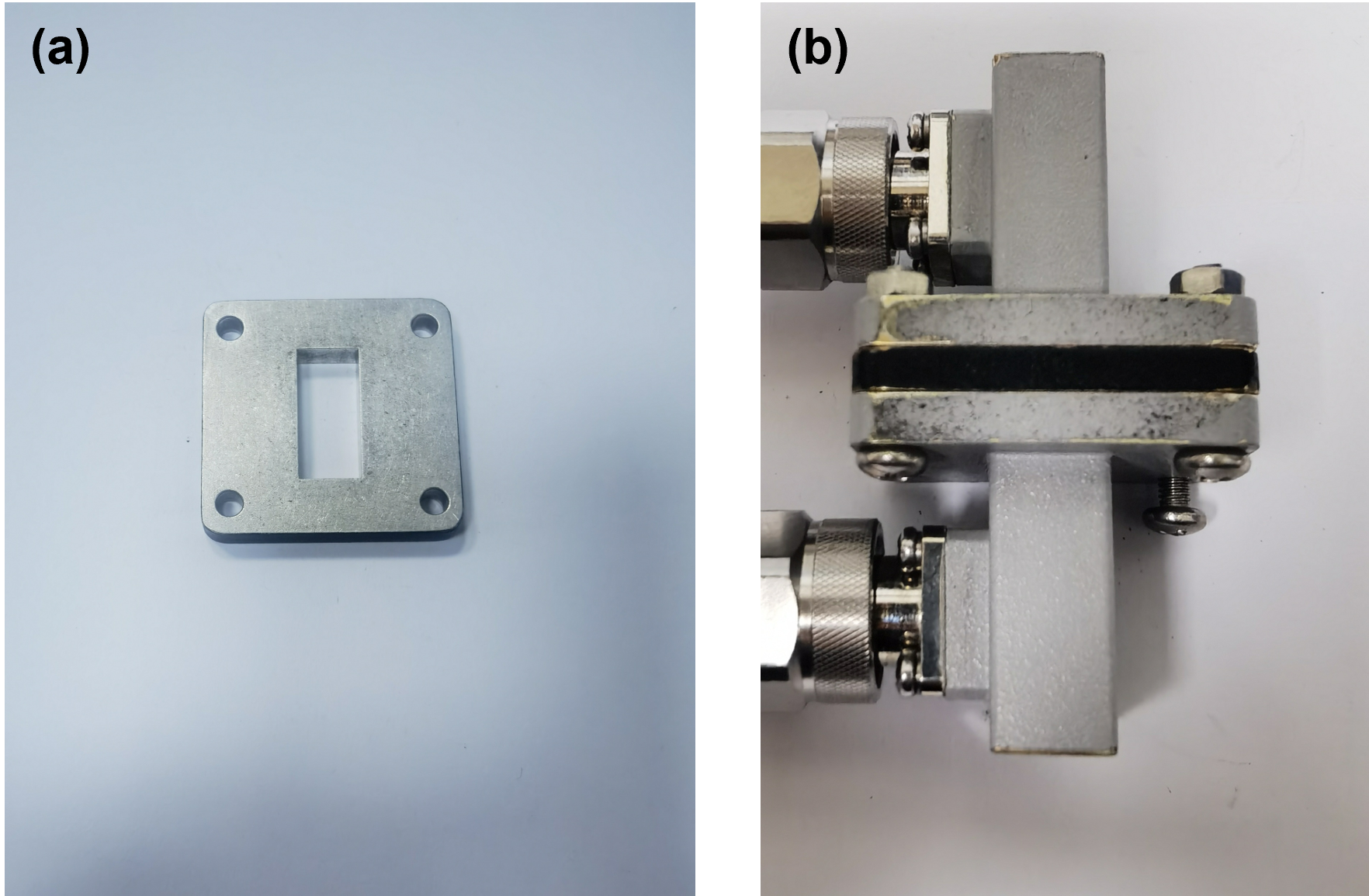


**Fig. S2** **a)** Photograph of the bracket used for installing BFFs samples and **b)** the connector for placing the mold.


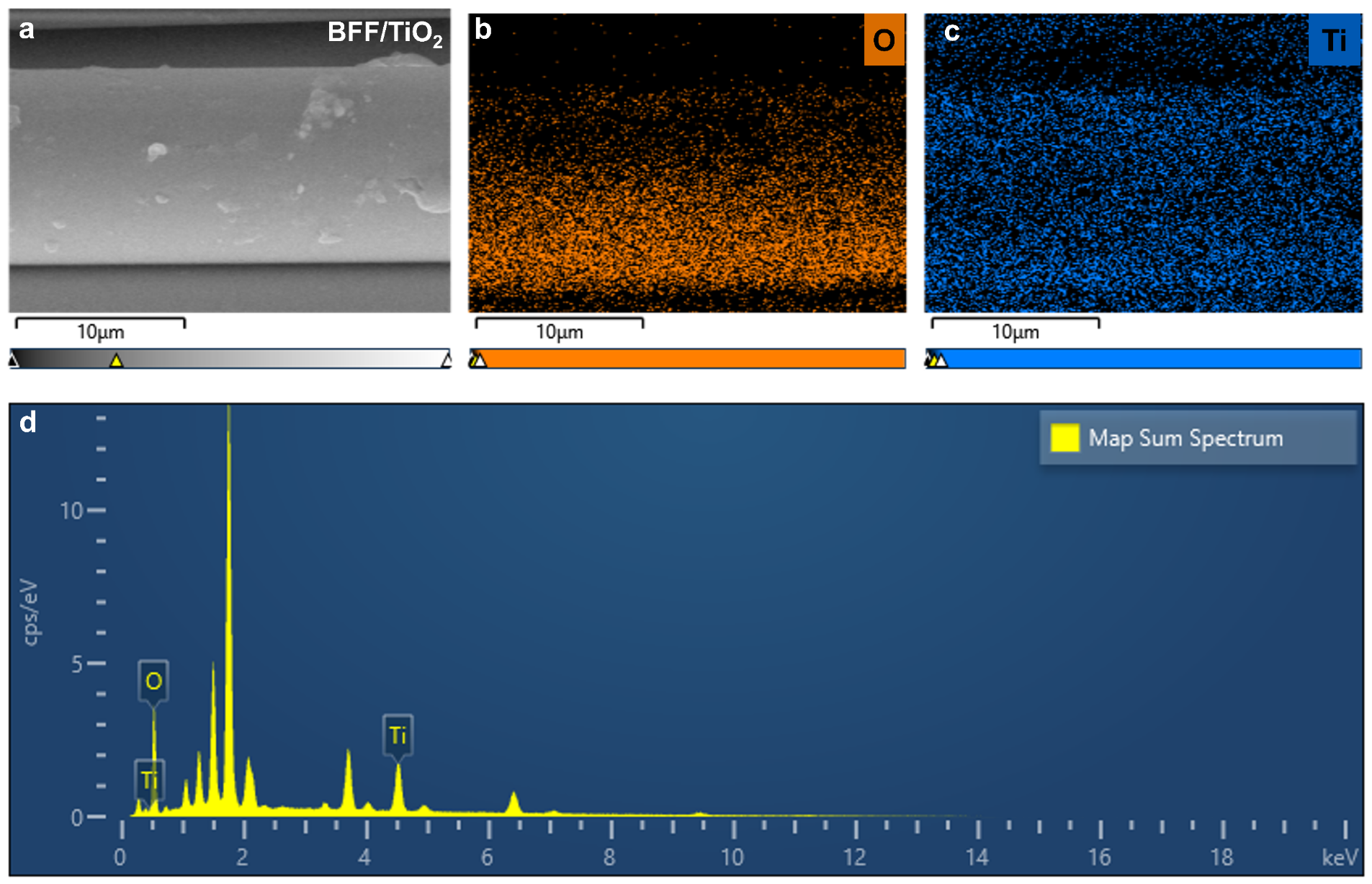


**Fig. S3 a**) SEM images, **b**) element mapping and **c**) Map Sum Spectrum of BFF/TiO_2_’s surface.


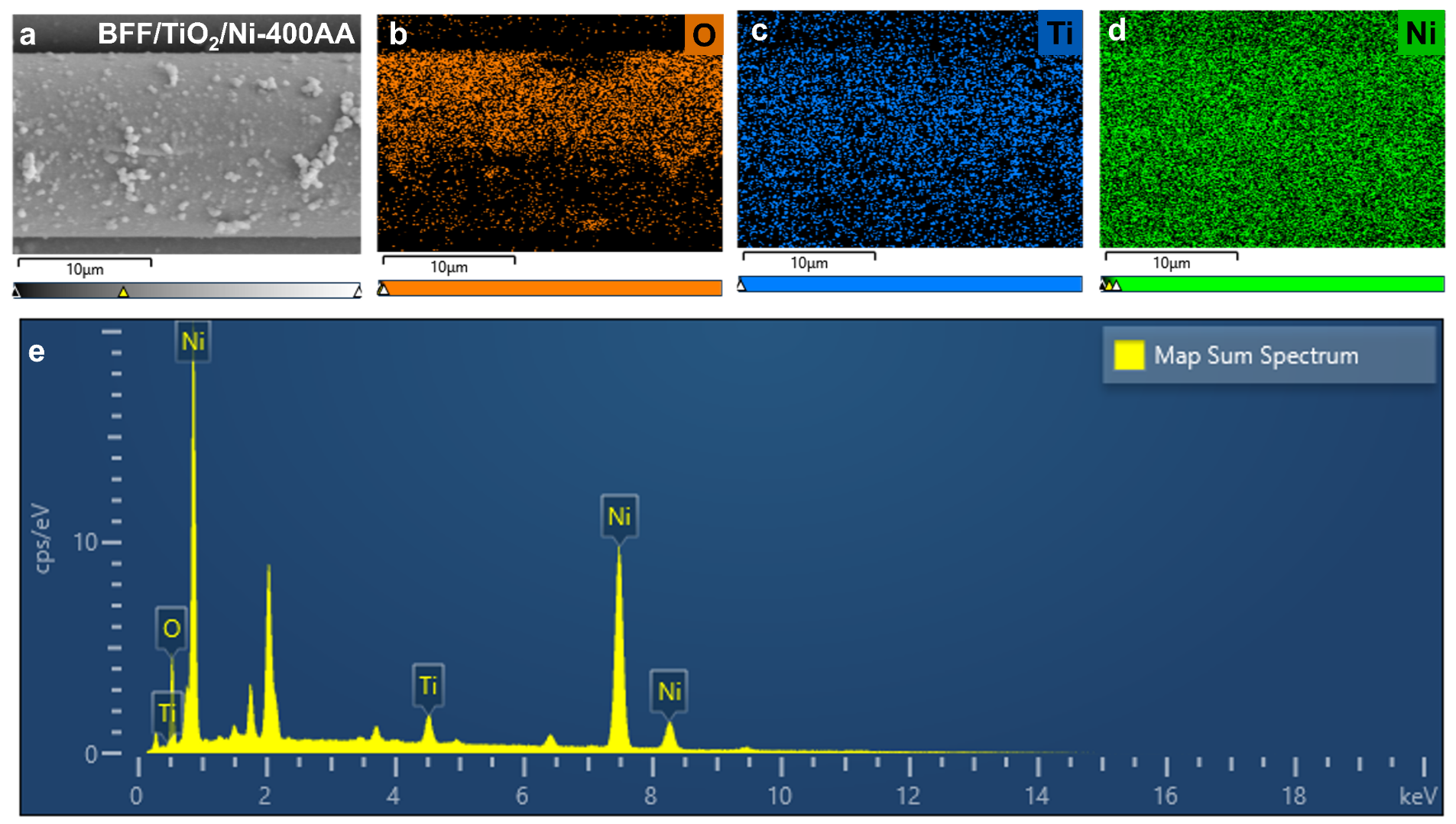


**Fig. S4 a**) SEM images, **b**) element mapping and **c**) Map Sum Spectrum of BFF/TiO_2_/Ni-400AA’s surface.


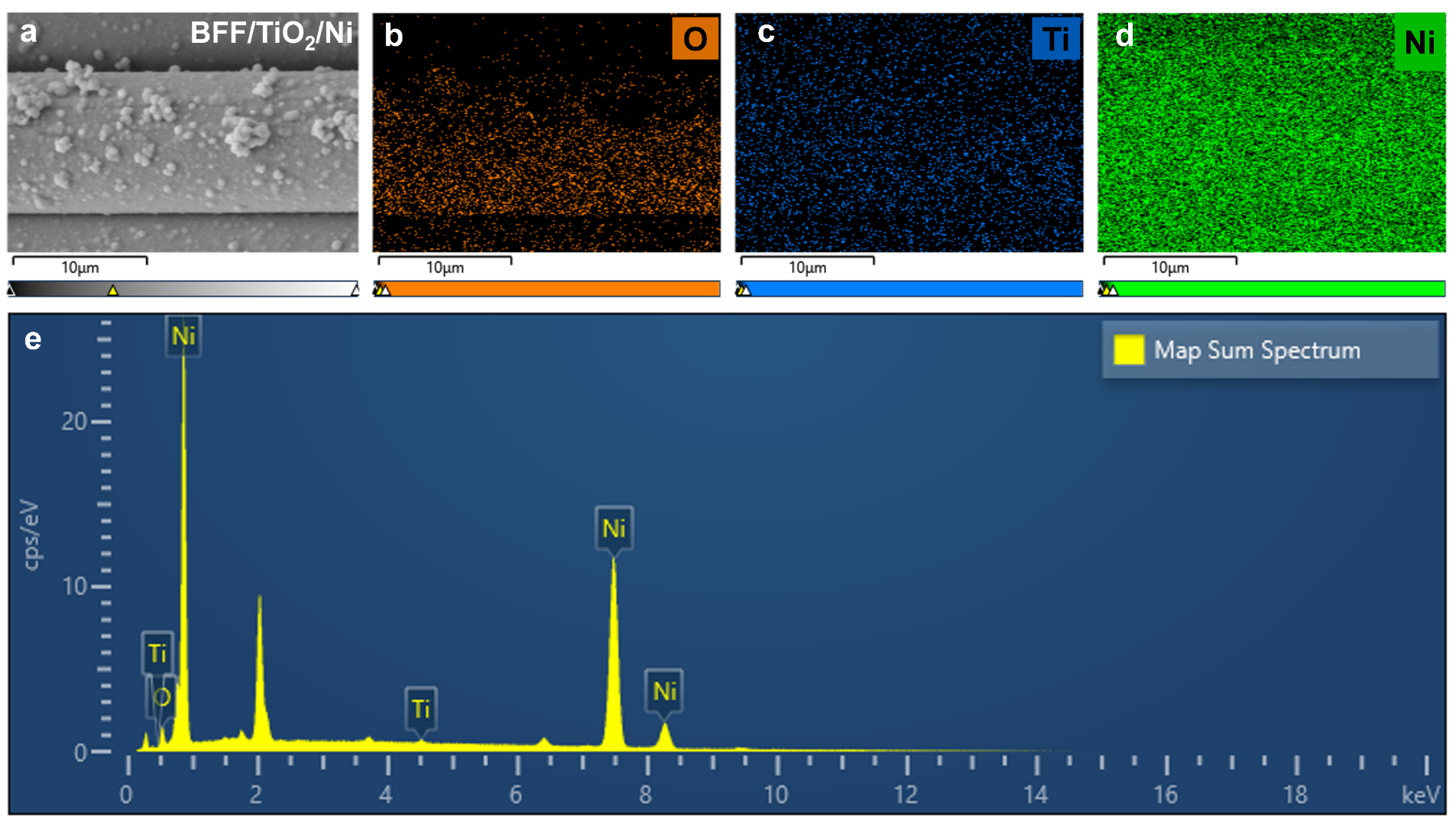


**Fig. S5 a**) SEM images, **b**) element mapping and **c**) Map Sum Spectrum of BFF/TiO_2_/Ni’s surface.


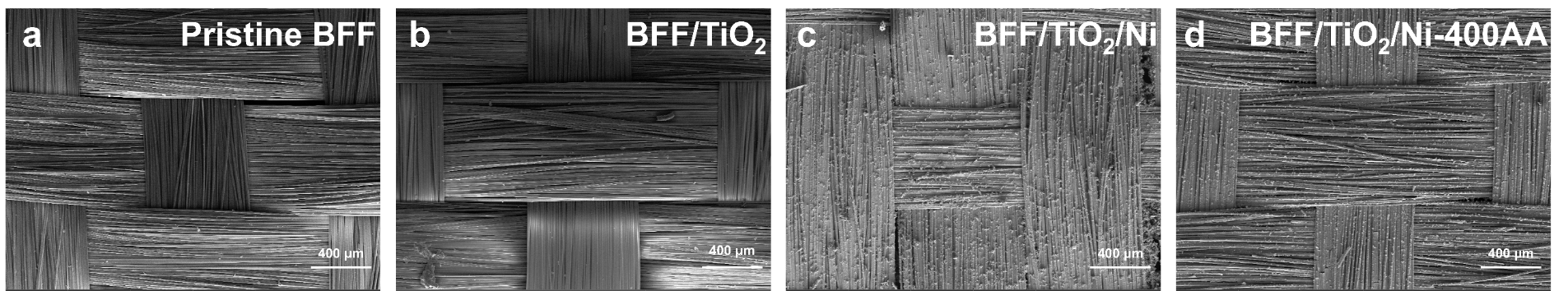


**Fig. S6** SEM images of **a**) pristine BFF, **b**) BFF/TiO_2_, **c**) BFF/TiO_2_/Ni and **d**) BFF/TiO_2_/Ni-400AA.


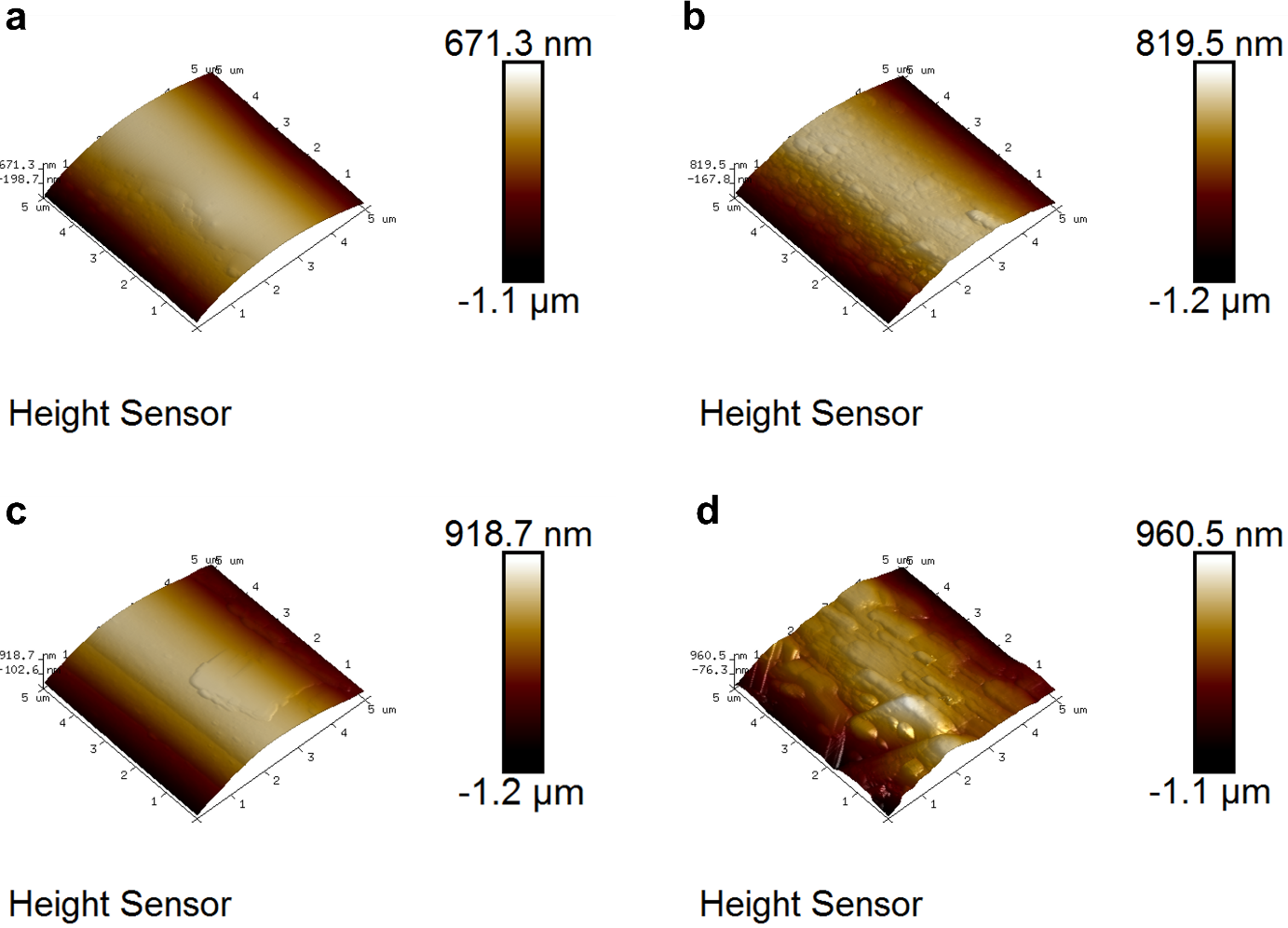


**Fig. S7** AFM images of **a**) pristine BFF, **b**) BFF/TiO_2_, **c**) BFF/TiO_2_/Ni, and **d**) BFF/TiO_2_/Ni-400AA.


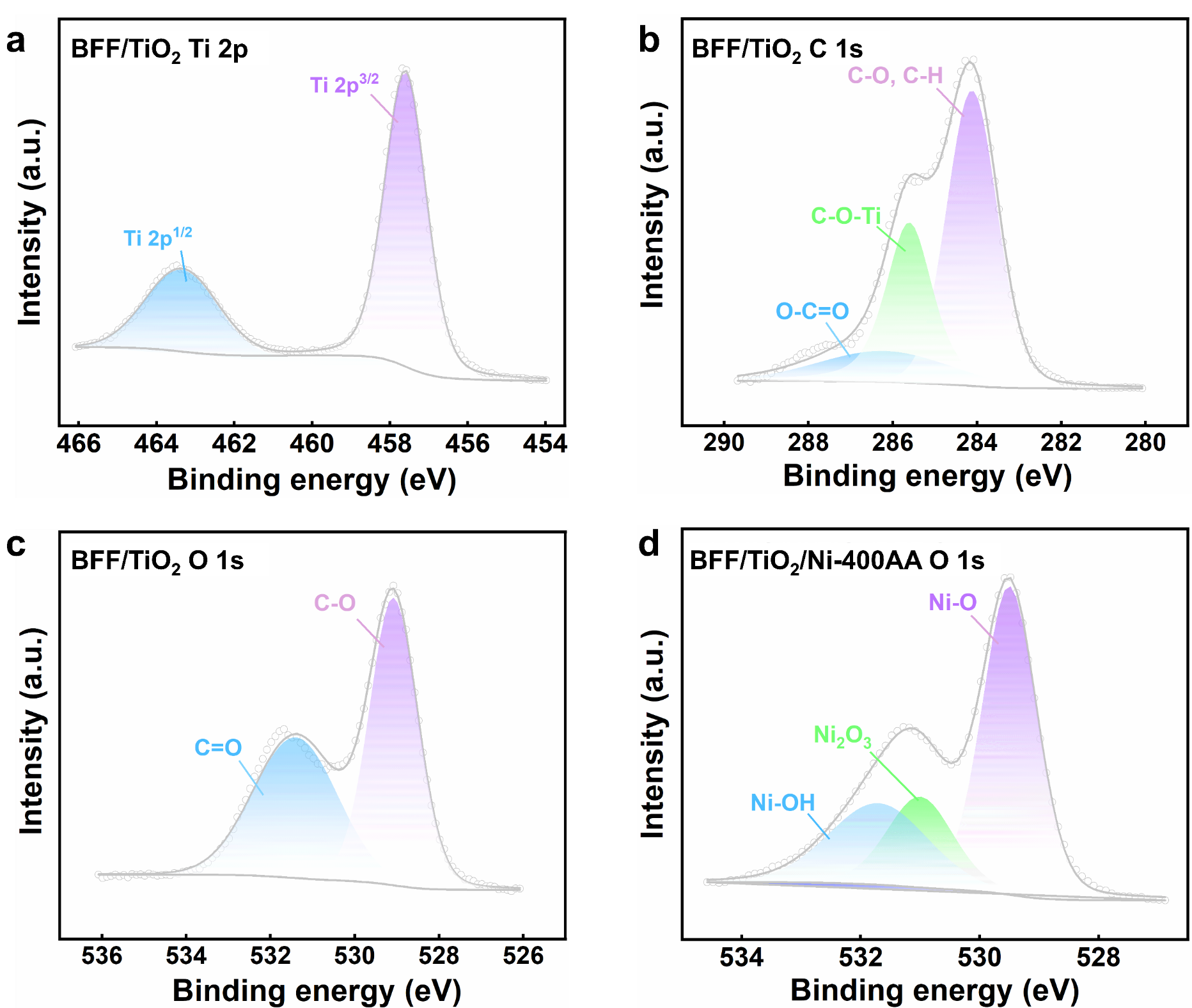


**Fig. S8** XPS spectra of **a**) Ti 2p, **b**) C 1s, **c**) O 1s of BFF/TiO_2_, and **d**) O 1s of BFF/TiO_2_/Ni-400AA.





**Fig. S9** XRD spectrum of pristine BFF, BFF/TiO_2_, and BFF/TiO_2_/Ni.





**Fig. S10** EMI SE of BFF/TiO_2_/Ni-450HA with different electroless plating times (10 min, 20 min, 30 min, 40 min, 50 min, and 60 min).


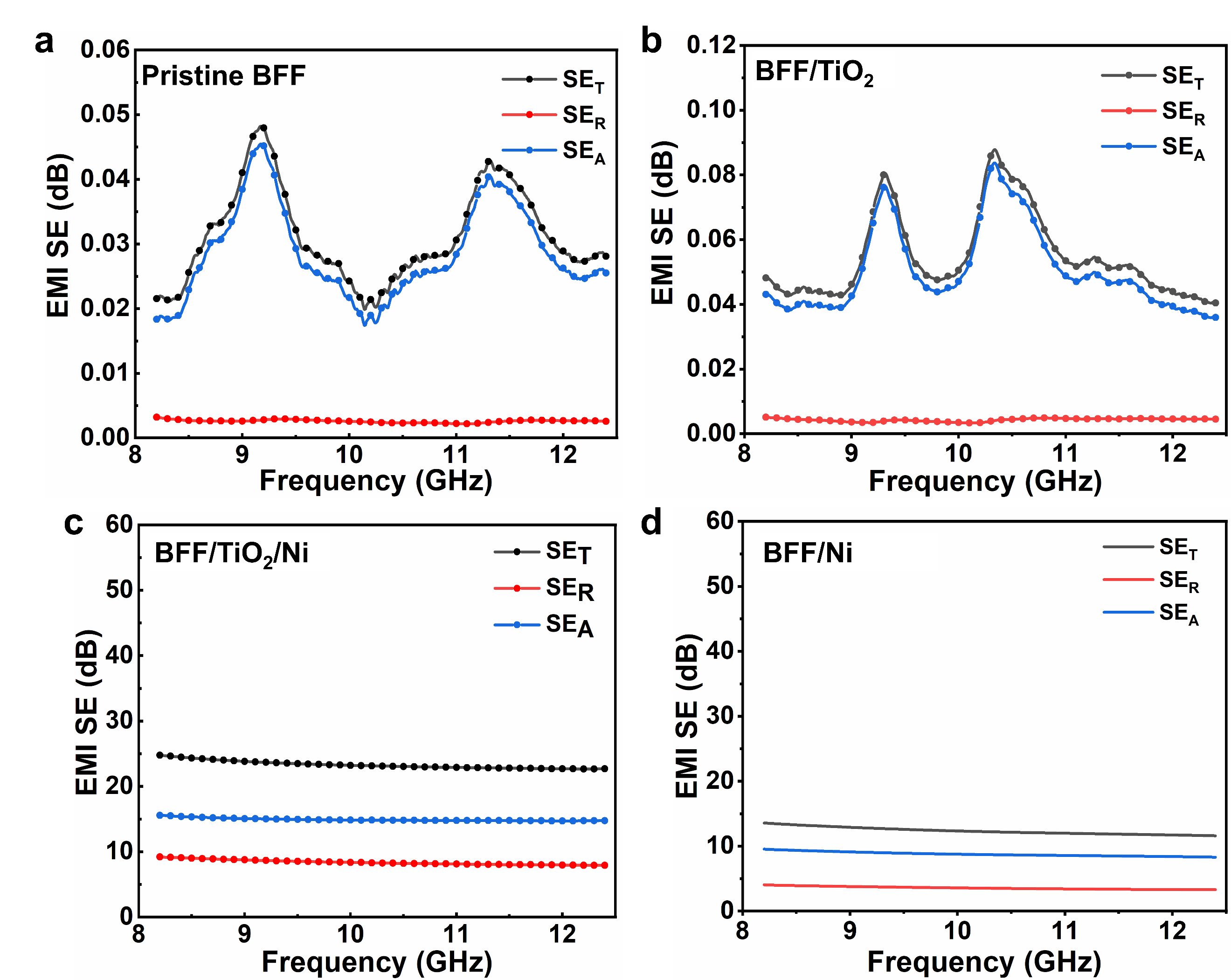


**Fig. S11** EMI SE of **a**) pristine BFF, **b**) BFF/TiO_2_, **c**) BFF/TiO_2_/Ni, and **d**) BFF/Ni (after plasma treatment).


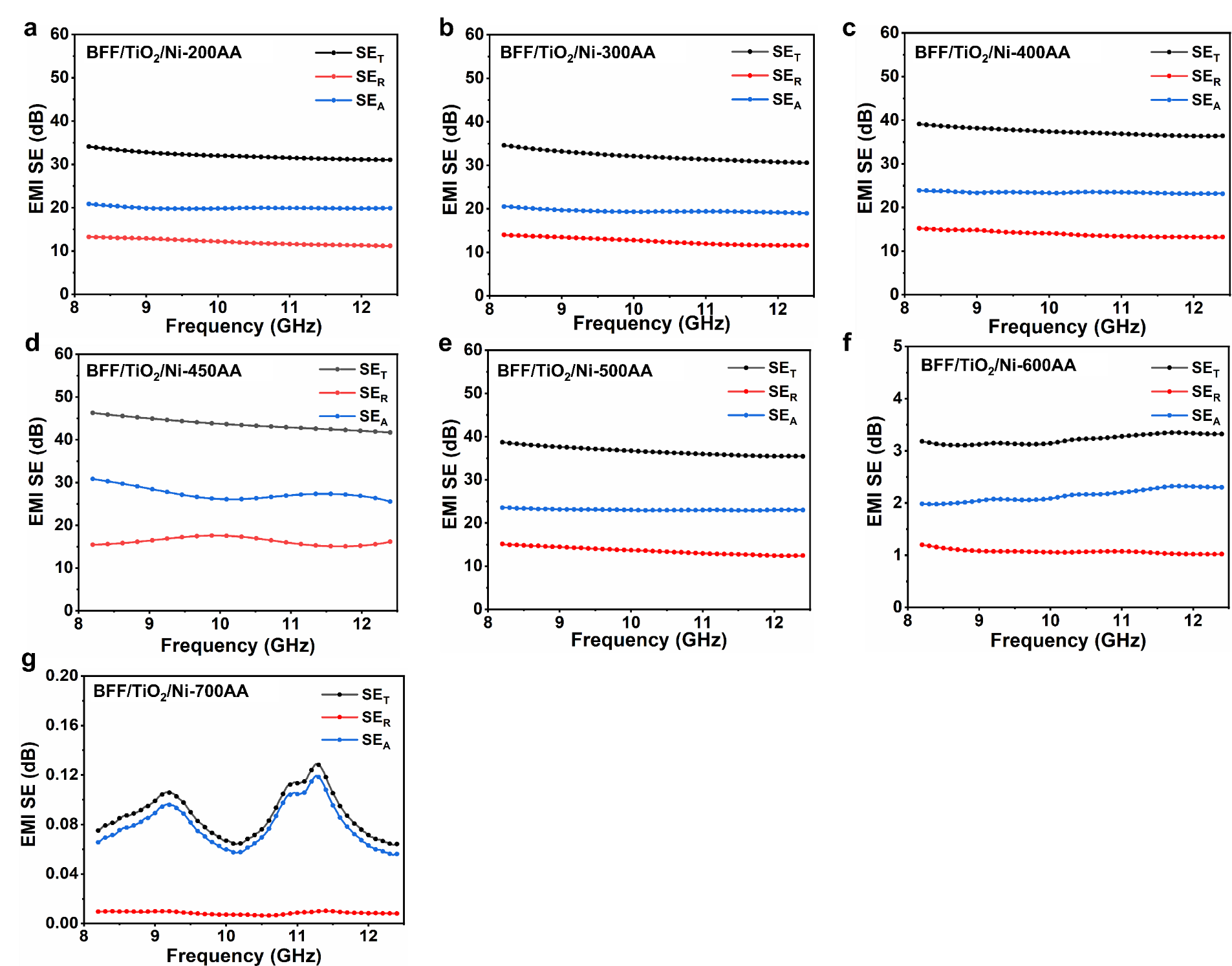


**Fig. S12** EMI SE of **a**) BFF/TiO_2_/Ni-200AA, **b**) BFF/TiO_2_/Ni-300AA, **c**) BFF/TiO_2_/Ni-400AA, **d**) BFF/TiO_2_/Ni-450AA, **e**) BFF/TiO_2_/Ni-500AA, **f**) BFF/TiO_2_/Ni-600AA, and **g**) BFF/TiO_2_/Ni-700AA.


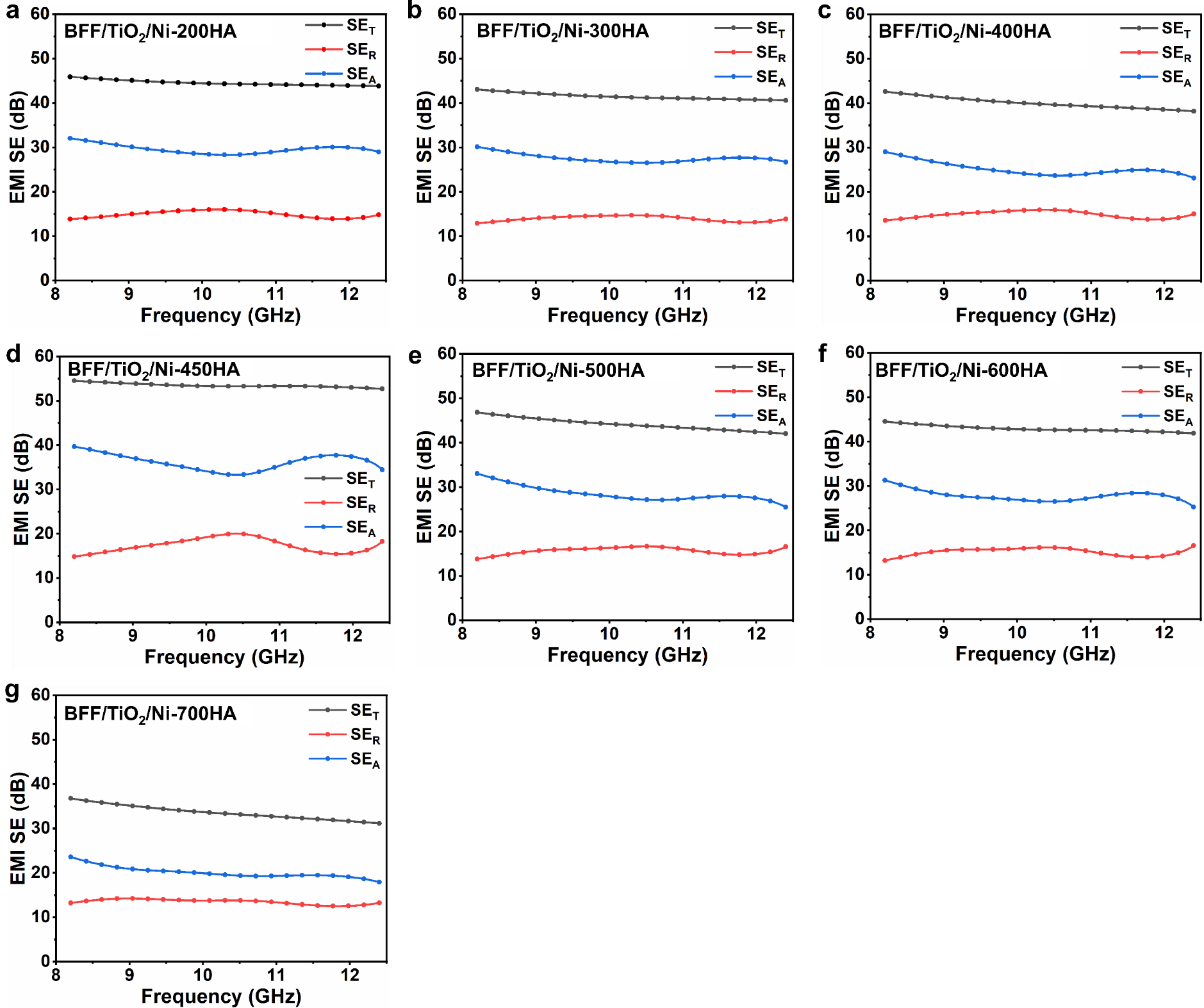


**Fig. S13** EMI SE of **a**) BFF/TiO_2_/Ni-200HA, **b**) BFF/TiO_2_/Ni-300HA, **c**) BFF/TiO_2_/Ni-400HA, **d**) BFF/TiO_2_/Ni-450HA, **e**) BFF/TiO_2_/Ni-500HA, **f**) BFF/TiO_2_/Ni-600HA, and **g**) BFF/TiO_2_/Ni-700HA.


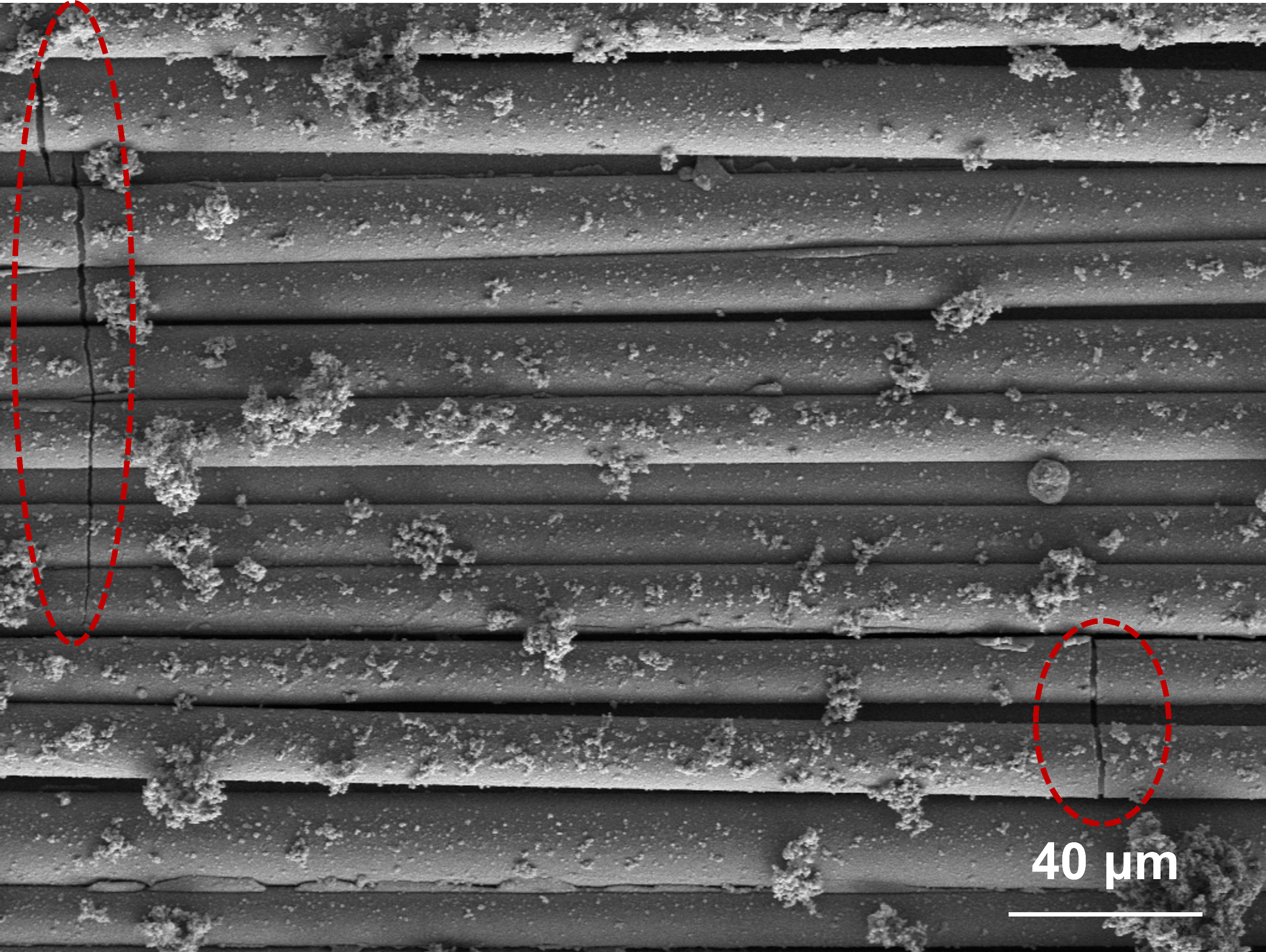


**Figure S14** SEM images of BFF/TiO_2_/Ni-600AA’s surface.


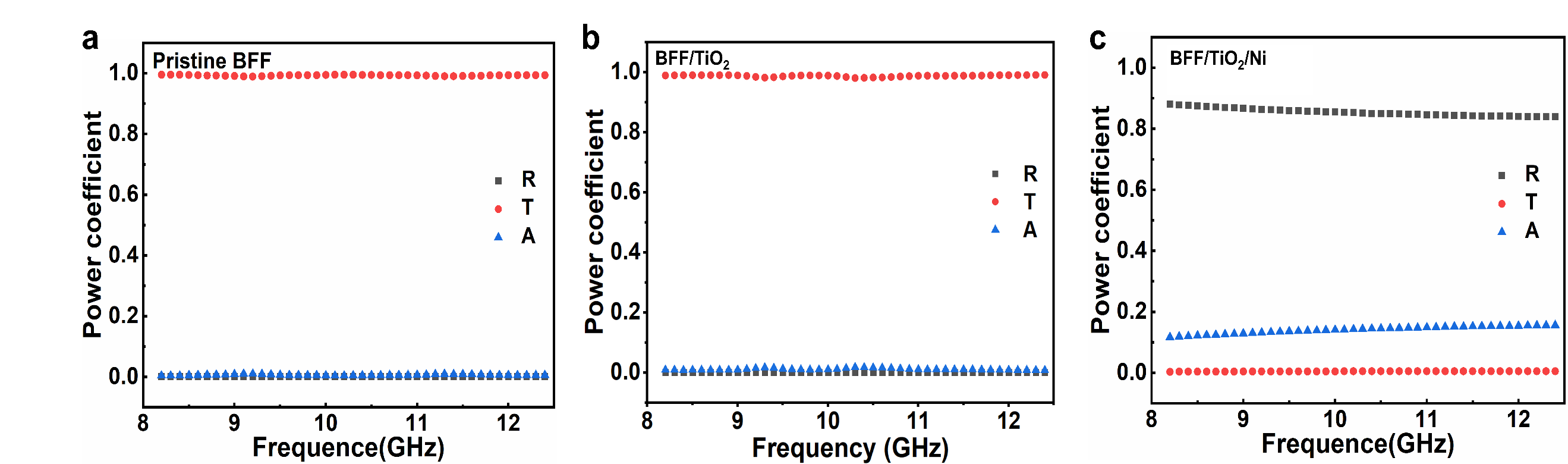


**Fig. S15** Power coefficient of **a**) pristine BFF, **b**) BFF/TiO_2_, and **c**) BFF/TiO_2_/Ni.


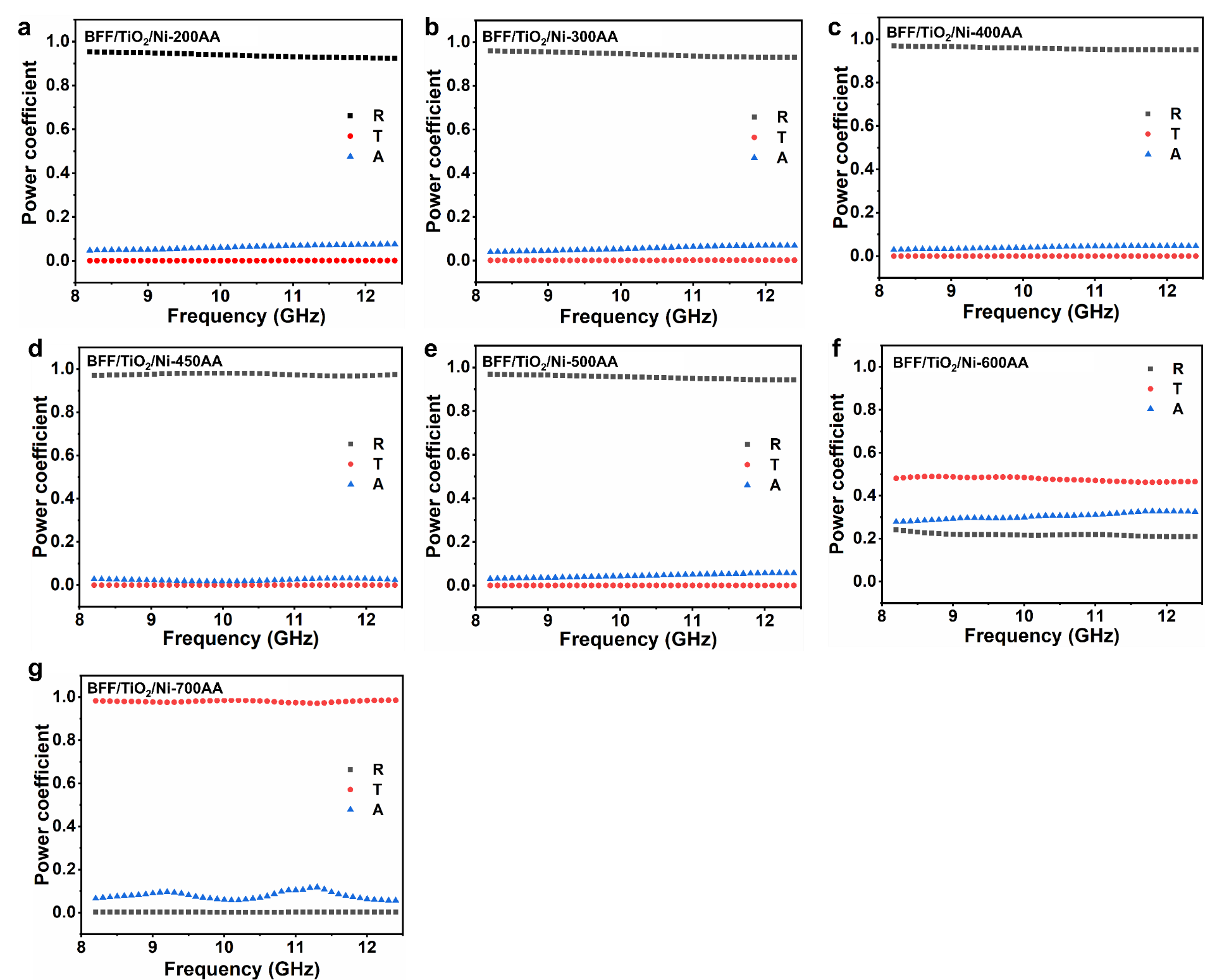


**Fig. S16** Power coefficient of **a**) BFF/TiO_2_/Ni-200AA, **b**) BFF/TiO_2_/Ni-300AA, **c**) BFF/TiO_2_/Ni-400AA, **d**) BFF/TiO_2_/Ni-450AA, **e**) BFF/TiO_2_/Ni-500AA, **f**) BFF/TiO_2_/Ni-600AA, and **g**) BFF/TiO_2_/Ni-700AA.


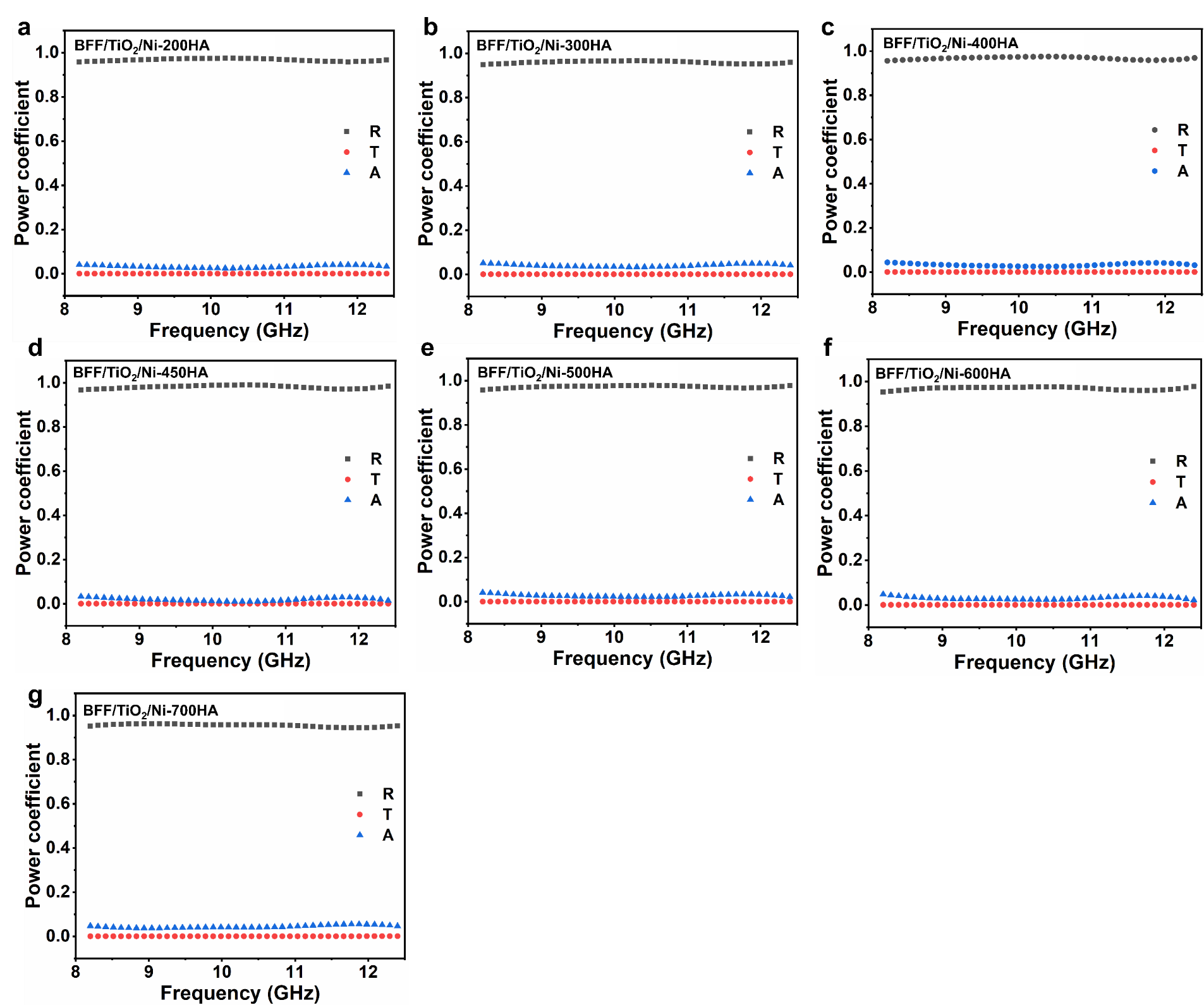


**Fig. S17** Power coefficient of **a**) BFF/TiO_2_/Ni-200HA, **b**) BFF/TiO_2_/Ni-300HA, **c**) BFF/TiO_2_/Ni-400HA, **d**) BFF/TiO_2_/Ni-450HA, **e**) BFF/TiO_2_/Ni-500HA, **f**) BFF/TiO_2_/Ni-600HA, and **g**) BFF/TiO_2_/Ni-700HA.


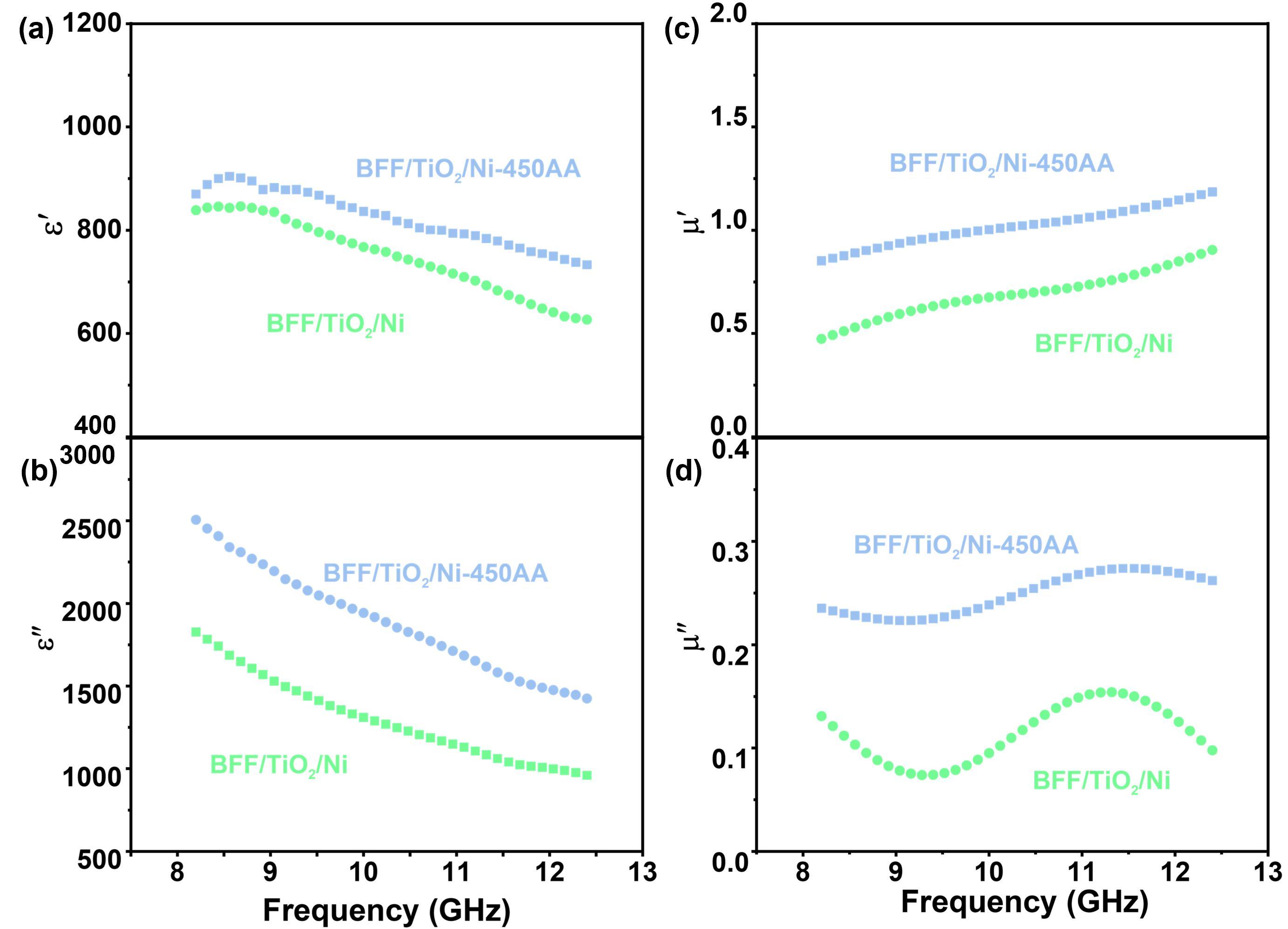


**Fig. S18** Comparison of EM parameters of BFF/TiO_2_/Ni and BFF/TiO_2_/Ni-450AA. a) Real part of the complex permittivity, b) Imaginary part of the complex permittivity, c) real part of the complex permeability, d) imaginary part of the complex permeability, e) tangent of the permittivity, f) tangent of the permeability.


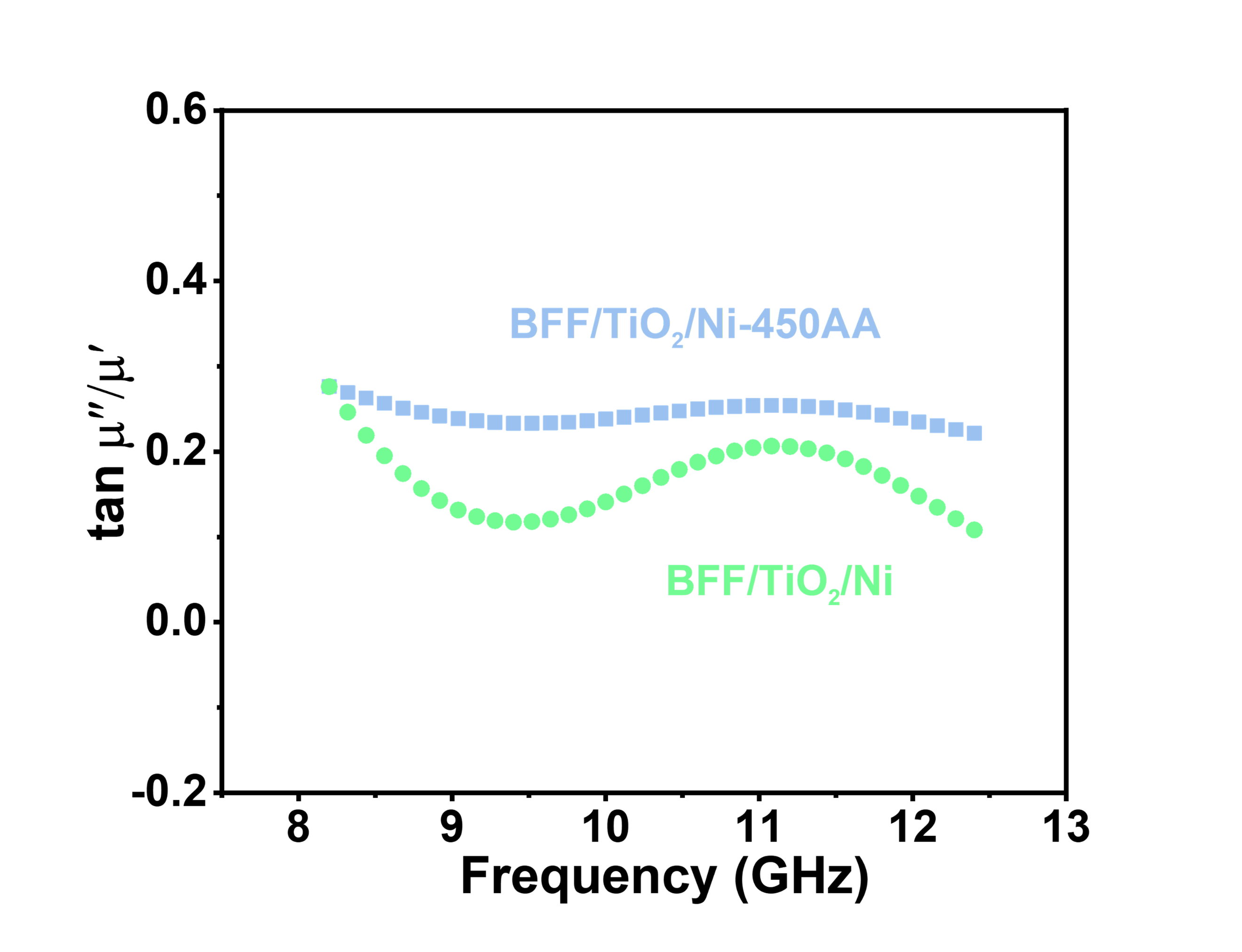


**Fig. S19** Tangent of the permeability for BFF/TiO_2_/Ni and BFF/TiO_2_/Ni-450AA.


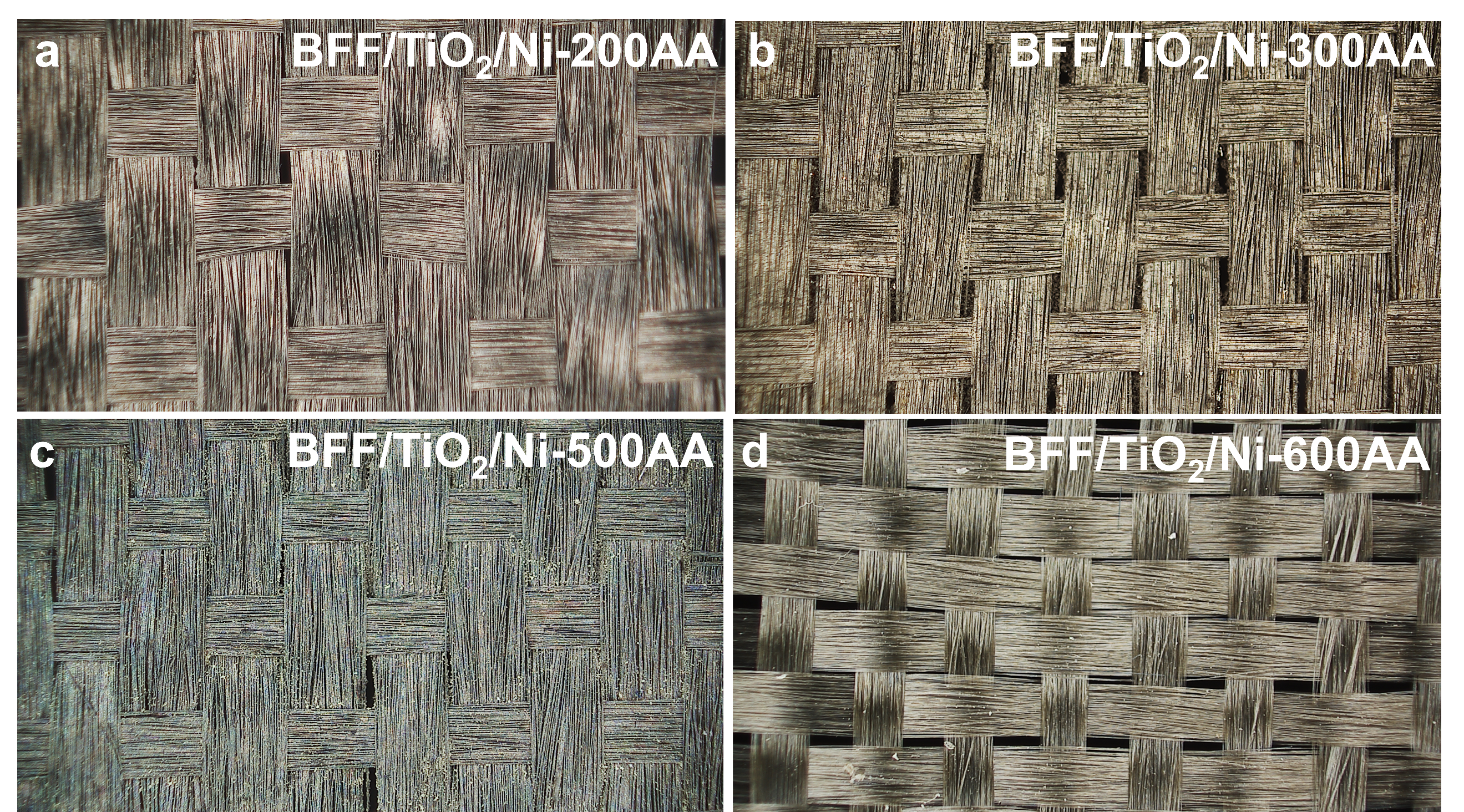


**Fig. S20** Optical microscopy images of colorful (a) BFF/TiO_2_/Ni-200AA, (b) BFF/TiO_2_/Ni-300AA, (c) BFF/TiO_2_/Ni-500AA, and (d) BFF/TiO_2_/Ni-600AA.


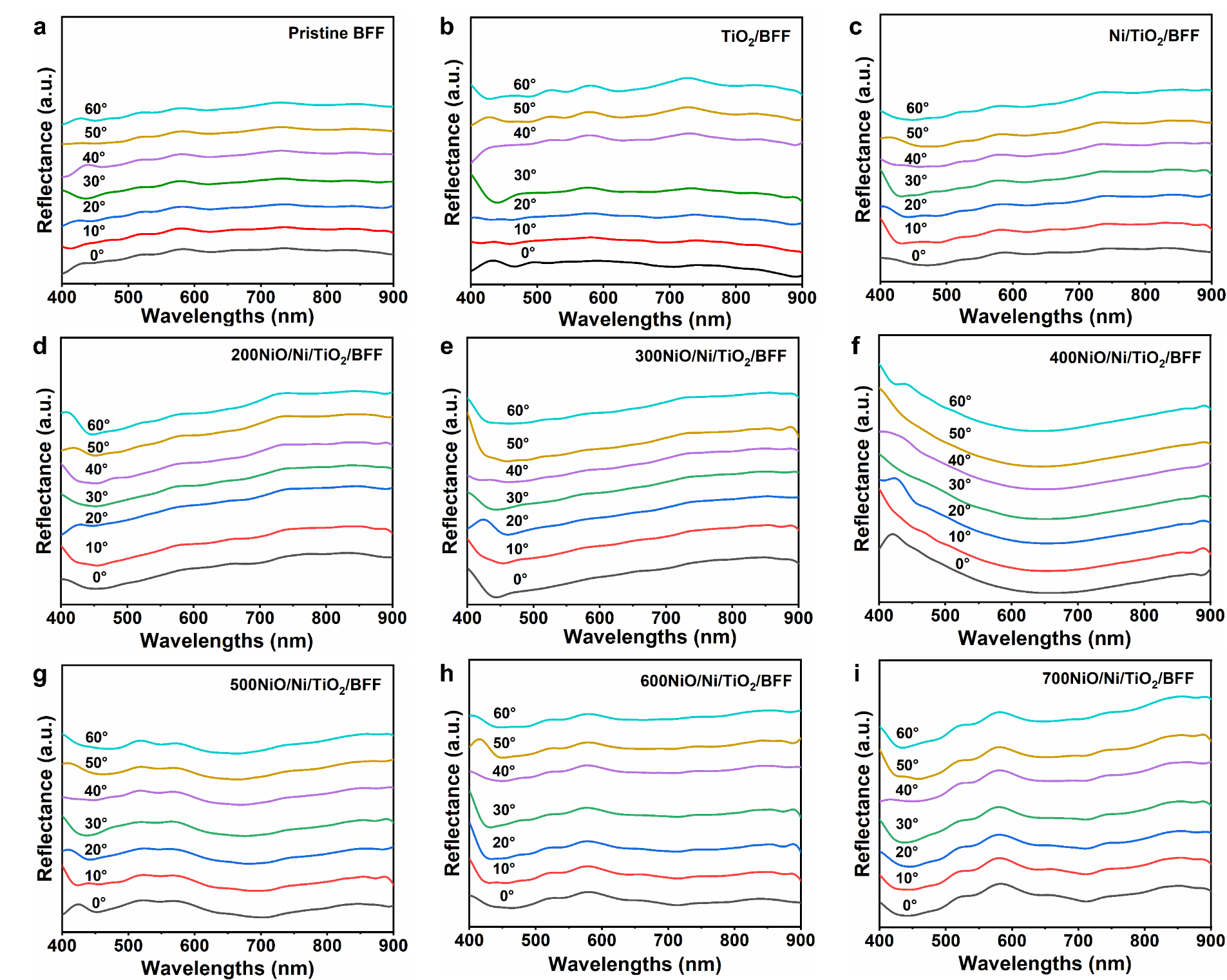


**Fig. S21** Different angle-resolved reflectance spectra of **a**) pristine BFF, **b**) BFF/TiO_2_, **c**) BFF/TiO_2_/Ni, **d**) BFF/TiO_2_/Ni-200AA, **e**) BFF/TiO_2_/Ni-300AA, **f**) BFF/TiO_2_/Ni-400AA, **g**) BFF/TiO_2_/Ni-500AA, **h**) BFF/TiO_2_/Ni-600AA, and **i**) BFF/TiO_2_/Ni-700AA.

**
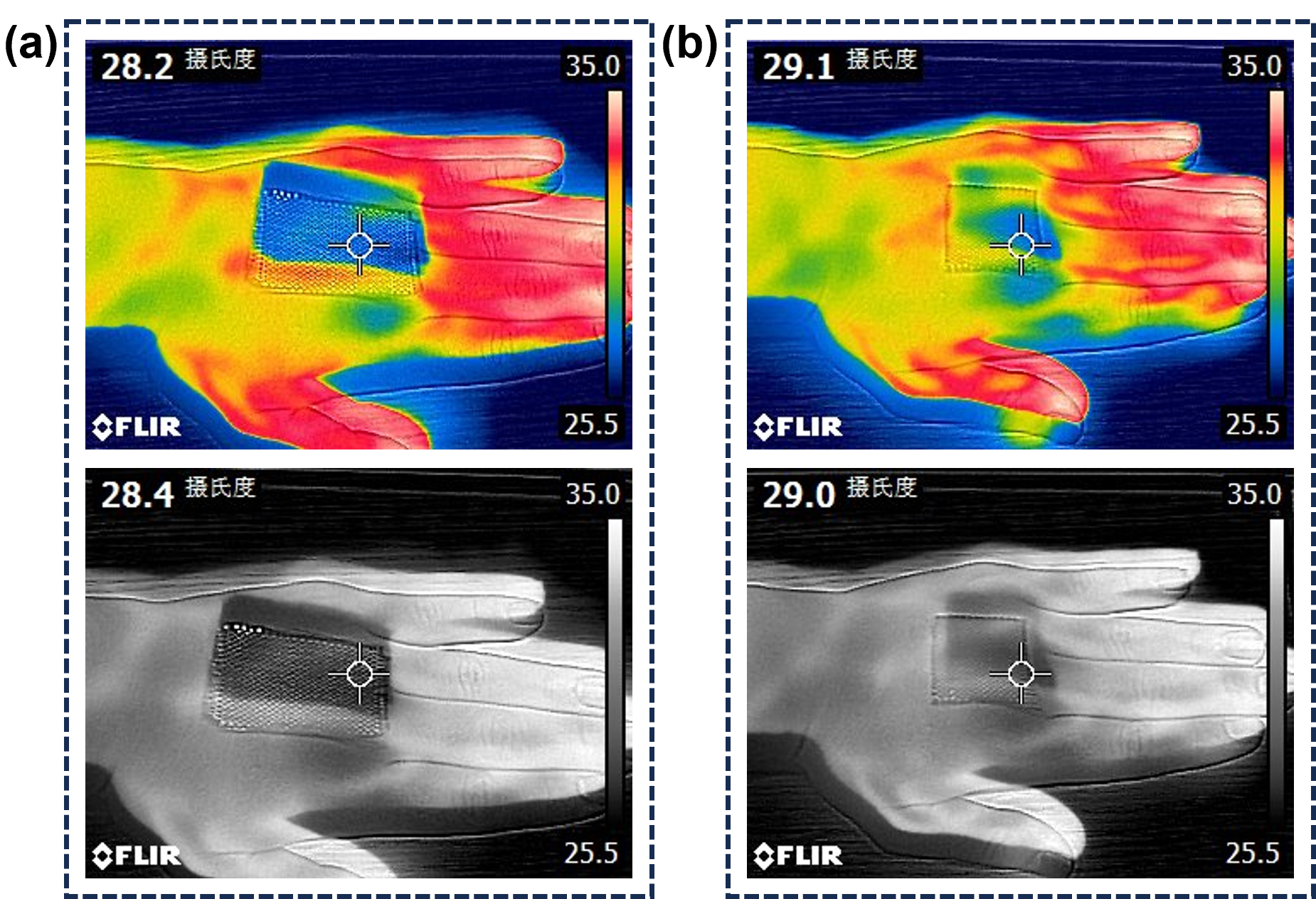
**

**Fig. S22** Thermal infrared image of (a) BFF/TiO_2_/Ni and (b) BFF/Ni placed upon a hand.

**Table S1** O, Ti and Ni elements on the fiber surface.

| Sample (surface) | Atomic (%) | | |
| --- | --- | --- | --- |
|  | O | Ti | Ni |
| BFF/TiO_2_ | 86.34 | 13.66 | - |
| BFF/TiO_2_/Ni | 5.41 | 0.43 | 94.16 |
| BFF/TiO_2_/Ni-400AA | 19.33 | 3.37 | 77.30 |

**Table S2** Resistance and electrical conductivity for BFF/TiO_2_/Ni, BFF/TiO_2_/Ni-450AA, and BFF/TiO_2_/Ni-450HA.

| Sample | Resistance (Ω) | Electrical Conductivity (s·cm^-1^) |
| --- | --- | --- |
| BFF/TiO_2_/Ni | 8.7 | 7.94 × 10^3^ |
| BFF/TiO_2_/Ni-450AA | 4.8 | 1.25 × 10^4^ |
| BFF/TiO_2_/Ni-450HA | 1.6 | 3.70 × 10^4^ |

**Table S3** Comparison of EMI SE and thickness of this work and previously reported EMI shielding composite materials.

| Shielding material | EMI SE  (dB) | Frequency  (GHz) | Thickness  (mm) | References |
| --- | --- | --- | --- | --- |
| rGO-GF/UP composites | 21.3 | 8.2-12.4 | 10 | [S1] |
| MWCNT/xGnP-GF composites (GF04) | 38.6 | 0.3-1.5 | 3.02±0.03 | [S2] |
| HNGF-CNTs-120 | ~40 | 8.2-12.4 | 1.5 | [S3] |
| 9.2 wt% GF@Ni-CNTs/epoxy | ~50 | 8.2-12.4 | 0.8 | [S4] |
| AF@Ni | 12.3-13.4 | 8.2-12.4 | 0.01438 | [S5] |
| AF@Ni/Cu | 17.0-19.4 | 8.2-12.4 | 0.01542 | [S5] |
| AF@Ni/Cu/Ni | 28.3-30.6 | 8.2-12.4 | 0.01724 | [S5] |
| 1 layer GRAF | 29.45 | 8.2-12.4 | 0.4 | [S6] |
| 5 layer GRAF | 67.86 | 8.2-12.4 | 2 | [S6] |
| BF/ANF/CNT composite paper | 24.6 | 8.2-12.4 | 0.048 | [S7] |
| BFF/CNTs/PDMS | 37.37 | 8.2-12.4 | 2 | [S8] |
| BFF-CNT/PDMS | 40.1 | 8.2-12.4 | 9 layers | [S9] |
| BF/T4/TNR | 41.53 | 8.2-12.4 | - | [S10] |
| Single layer MNF-3 | 35.7 | 8.2-12.4 | 1.04 | [S11] |
| EMA/Fe_3_O_4_@g-C_3_N_4_ composite | 32.4 | 8.2-12.4 | 2 | [S12] |
| 5 Graphene-0.5CNT-Al_2_O_3_ | 39 | 8.2-12.4 | - | [S13] |
| Ni/PVA composite film | 30.5 | 8.2-12.4 | - | [S14] |
| NF modified cement mortar | 22.1 | 8.2-12.4 | 10 | [S15] |
| NiO@Carbon@Polypyrrole | 21.9 | 8.2-12.4 | 6 | [S16] |
| Nw/PS nanocomposites | 25 | 8.2-12.4 | 0.21 | [S17] |
| 3% NiO/Graphene/PVDF | 39 | 8-13 | - | [S18] |
| Laser-induced graphene/basalt fiber reinforced expoxy | ~20 | 8.2-12.4 | 1 layer LIG | [15] |
| Laser-induced graphene/basalt fiber reinforced expoxy | ~50 | 8.2-12.4 | 3 layers LIG | [15] |
| BF/CNT120/epoxy | 30.38 | 8.2-12.4 | 1.4 | [18] |
| BiSn/BF/CNT/epoxy | 30.4 | 8.2-12.4 | 3 plies CNT | [19] |
| 0.1% MXene-1% PU-EP/CF | 27.8 | 8.2-12.4 | 2 | [S19] |
| EVA/MXene/EVA-100 | 49.6 | 8.2-12.4 | 0.1 | [S20] |
| CNT@MXene fiber | 23.4 | 8.2-12.4 | 1.19 | [S21] |
| 60 wt% CuNWs/MXene/ANFs hybrid film | 32.38 | 8.2-12.4 | 0.047 | [S22] |
| FCFe/MXene membrane | 44.56 | 8.2-12.4 | 0.0849 | [S23] |
| BFF/TiO_2_/Ni | 23.28 | 8.2-12.4 | 0.13 | **this work** |
| BFF/TiO_2_/Ni-450AA | 43.65 | 8.2-12.4 | 0.13 | **this work** |
| BFF/TiO_2_/Ni-450HA | 53.47 | 8.2-12.4 | 0.13 | **this work** |

**Supplementary References**

1. Wan, X.; Lu, H.; Kang, J.; Li, S.; Yue, Y. Preparation of graphene-glass fiber-resin composites and its electromagnetic shielding performance. *Composite Interfaces* **2018**, *25* (10), 883-900. DOI: https://doi.org/10.1080/09276440.2018.1439641.
2. Gnidakouong, J. R. N.; Kim, J.-H.; Kim, H.; Park, Y.-B. Electromagnetic interference shielding behavior of hybrid carbon nanotube/exfoliated graphite nanoplatelet coated glass fiber composites. *Materials Science and Engineering: B* **2019**, *248*, 114403. DOI: https://doi.org/10.1016/j.mseb.2019.114403.
3. Kim, D.-K.; Han, W.; Kim, K.-W.; Kim, B.-J. Electromagnetic interference shielding effectiveness of direct-grown-carbon nanotubes/carbon and glass fiber-reinforced epoxy matrix composites. *Materials* **2023**, *16* (7), 2604. DOI: https://doi.org/10.3390/ma16072604.
4. Liu, Y.; He, D.; Dubrunfaut, O.; Zhang, A.; Pichon, L.; Bai, J. In-situ growing carbon nanotubes on nickel modified glass fiber reinforced epoxy composites for EMI application. *Applied Composite Materials* **2021**, *28*, 777-790. DOI: https://doi.org/10.1007/s10443-021-09894-y.
5. Tang, J.; Zhang, X.; Wang, J.; Zou, R.; Wang, L. Achieving flexible and durable electromagnetic interference shielding fabric through lightweight and mechanically strong aramid fiber wrapped in highly conductive multilayer metal. *Applied Surface Science* **2021**, *565*, 150577. DOI: https://doi.org/10.1016/j.apsusc.2021.150577.
6. Guo, Q.; Tian, H.; Cheng, Y.; Wang, S.; Li, Z.; Hao, H.; Liu, J.; Jiao, K.; Gao, X.; Zhang, J. Structural-functional integrated graphene-skinned aramid fibers for electromagnetic interference shielding. *ACS nano* **2024**, *18* (49), 33566-33575. DOI: https://doi.org/10.1021/acsnano.4c11782.
7. Song, S.; Li, L.; Ji, D.; Zhao, J.; Wu, Q.; Wang, Q. Flexible basalt fiber/aramid nanofiber/carbon nanotube electromagnetic shielding paper with outstanding environmental stability and joule heating performance. *ACS Applied Materials & Interfaces* **2023**, *15* (29), 35495-35506. DOI: https://doi.org/10.1021/acsami.3c06138.
8. Chen, Z.-X.; Chang, C.; Yue, X.; Li, H.; Liang, C.-G.; Ma, P.-C. Experimental and theoretical study on the electromagnetic shielding performance of polymer nanocomposites consisting of basalt fiber and CNTs. *Composites Science and Technology* **2024**, *247*, 110399. DOI: https://doi.org/10.1016/j.compscitech.2023.110399.
9. Chang, C.; Yue, X.; Hao, B.; Xing, D.; Ma, P.-C. Direct growth of carbon nanotubes on basalt fiber for the application of electromagnetic interference shielding. *Carbon* **2020**, *167*, 31-39. DOI: https://doi.org/10.1016/j.carbon.2020.05.074.
10. Yu, J.; Cui, Z.; Lu, J.; Zhao, J.; Zhang, Y.; Fan, G.; Liu, S.; He, Y.; Yu, Y.; Qi, D. Integrated hierarchical macrostructures of flexible basalt fiber composites with tunable electromagnetic interference (EMI) shielding and rapid electrothermal response. *Composites Part B: Engineering* **2021**, *224*, 109193. DOI: https://doi.org/10.1016/j.compositesb.2021.109193.
11. Wang, X. F.; Lei, Z. W.; Ma, X. D.; He, G. F.; Xu, T.; Tan, J.; Wang, L. L.; Zhang, X. S.; Qu, L. J.; Zhang, X. J. A lightweight MXene-Coated nonwoven fabric with excellent flame Retardancy, EMI Shielding, and Electrothermal/Photothermal conversion for wearable heater. *Chemical Engineering Journal* **2022**, *430*, 132605. DOI: https://doi.org/10.1016/j.cej.2021.132605.
12. Katheria, A.; Das, P.; Singh, H.; Nayak, J.; Paul, S.; Biswas, S.; Das, N. C. Highly flexible EMA/Fe_3_O_4_@ g-C_3_N_4_ composite for thermal control and EMI shielding application. *Colloids and Surfaces A: Physicochemical Engineering Aspects* **2024**, *700*, 134756. DOI: https://doi.org/10.1016/j.colsurfa.2024.134756.
13. Zhang, Z. J.; Huang, Y. J.; Zhang, P.; Wan, C. L. Outstanding EMI shielding properties of Al_2_O_3_ reinforced with sandwiched graphene/CNTs. *Journal of the European Ceramic Society* **2023**, *43* (9), 4082-4087. DOI: https://doi.org/10.1016/j.jeurceramsoc.2023.02.068.
14. Joshi, M. A.; Khanna, P. K.; Mohammad, N.; Joshi, P. G. Synthesis and EMI shielding study of Ni (II) semicarbazone driven nickel nanoparticles. *Materials Chemistry and Physics* **2025**, *329*, 130064. DOI: https://doi.org/10.1016/j.matchemphys.2024.130064.
15. Yan, Y.; Du, X.; Kuang, Y. Study on the influence of nickel-iron alloy powder on the mechanical strength and electromagnetic shielding effectiveness of iron ore tailings/cement composite. *Case Studies in Construction Materials* **2024**, *21*, e03740. DOI: https://doi.org/10.1016/j.cscm.2024.e03740.
16. Anwar, U.; Sultan, N.; Yasmeen, G.; Shati, K.; Nadeem, M. Temperature-dependent conduction mechanism of NiO@Carbon@Polypyrrole nanomaterial with EMI shielding characteristics. *Heliyon* **2023**, *9* (12), e23193. DOI: https://doi.org/10.1016/j.heliyon.2023.e23193.
17. Gelves, G. A.; Al-Saleh, M. H.; Sundararaj, U. Highly electrically conductive and high performance EMI shielding nanowire/polymer nanocomposites by miscible mixing and precipitation. *Journal of Materials Chemistry* **2011**, *21* (3), 829-836. DOI: https://doi.org/10.1039/C0JM02546A.
18. Dinakaran, K.; Senthamilselvi, S.; Gayathri, T.; Kavitha, N.; Roy, D. Enhanced electromagnetic interference shielding in nickel oxide and graphene composite nanostructures loaded polyvinylidene difluoride thin films. *Journal of the Indian Chemical Society* **2023**, *100* (10), 101091. DOI: https://doi.org/10.1016/j.jics.2023.101091.
19. Hu Y; He X; Liu Y; Yang Y; Ma C. H; Sun Z. W; Jiang J. J. Multifunctional Carbon Fiber Composites via Interface-Engineered MXene-Polyurethane Hybrids: Synergistically Enhanced Mechanical, In Situ Damage Sensing, and EMI Shielding Properties. *ACS Applied Nano Materials* **2025**, *8* (21), 10985-11001. DOI: https://doi.org/10.1021/acsanm.5c01180.
20. Zeng Z. Y; Li J; Fang L. M; Zheng C. R; Chen H. M; Huang J; Qiao K; Li H. X; Li W. B. Flexible and durable shape memory EVA/MXene/EVA fiber membrane for programmable EMI shielding. *Chemical Engineering Journal* **2024**, *501*, 157599. DOI: https://doi.org/10.1016/j.cej.2024.157599.
21. Feng W. D; Zou L. H; Lan C. T; E S. J; Pu X. Core-sheath CNT@ MXene fibers toward absorption-dominated electromagnetic interference shielding fabrics. *Advanced Fiber Materials* **2024**, *6* (5), 1657-1668. DOI: https://doi.org/10.1007/s42765-024-00452-2.
22. Jia F. F; Dong J. Y; Dai X. Y; Liu Y. Q; Wang H.R; Lu Z. Q. Robust, flexible, and stable CuNWs/MXene/ANFs hybrid film constructed by structural assemble strategy for efficient EMI shielding. *Chemical Engineering Journal* **2023**, *452*, 139395. DOI: https://doi.org/10.1016/j.cej.2022.139395.
23. Shao R. Z; Wang G. L; Chai J. L; Lin J; Zhao G. Q; Zeng Z. H; Wang G. Z. Multifunctional Janus-structured polytetrafluoroethylene-carbon nanotube-Fe_3_O_4_/MXene membranes for enhanced EMI shielding and thermal management. *Nano-Micro Letters* **2025**, *17* (1), 1-22. DOI: https://doi.org/10.1007/s40820-025-01647-x.
