# Supplementary material for: Collaborative Optimization of Electromagnetic Interference Shielding, Adaptive Multi‐Color, and Thermal Camouflage of Basalt Fibers by Temperature‐Induced Gradient Structure Control
Source: Exploration (Beijing). 2026 Feb 18;6(2):70135. doi: 10.1002/exp2.70135 (PMC13094530; doi:10.1002/exp2.70135)
Supplement: Supplementary file 3 — Supporting File 3: exp270135‐sup‐0003‐data.pptx. [file EXP2-6-70135-s003.pptx]

## Slide 1
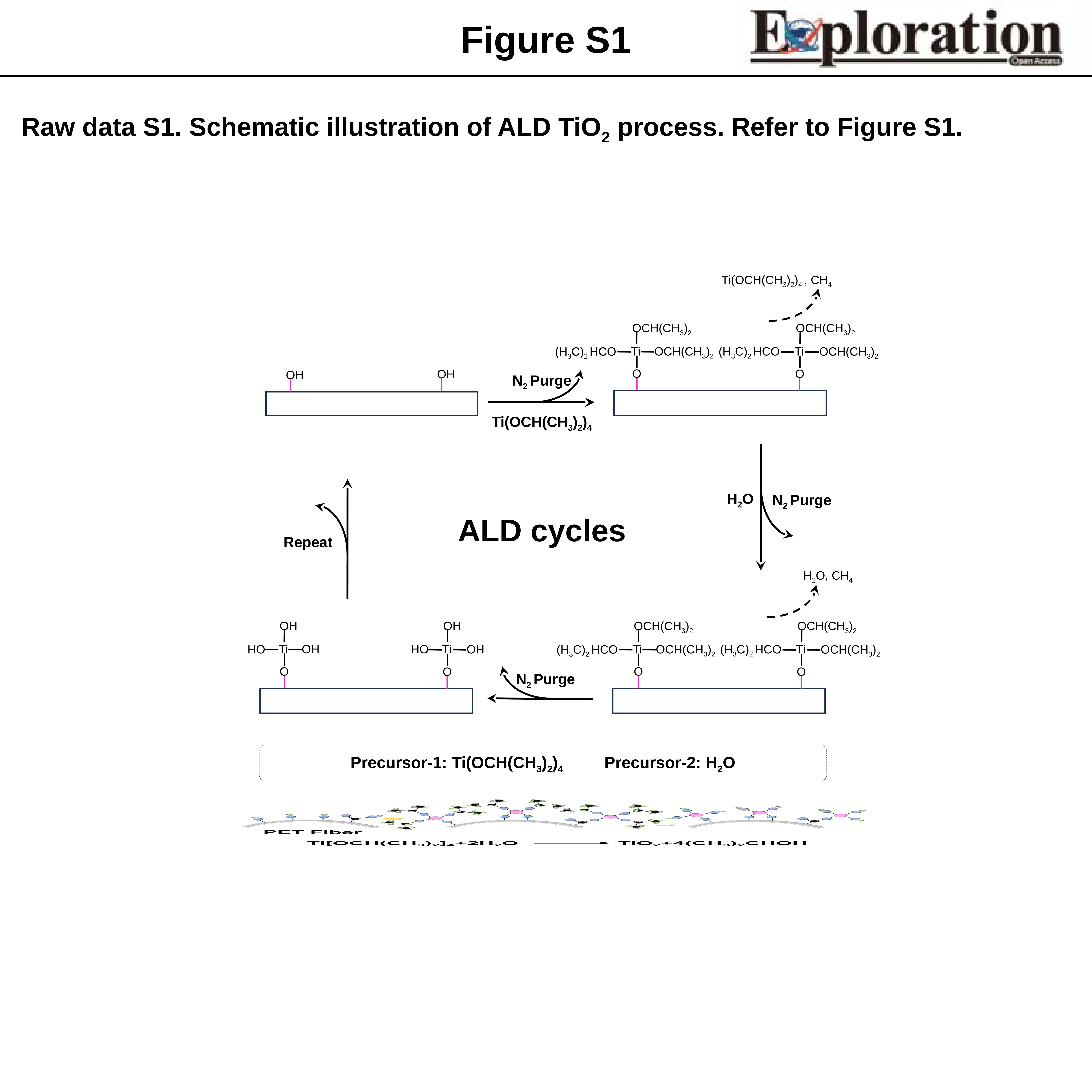

Figure S1
Raw data S1. Schematic illustration of ALD TiO2 process. Refer to Figure S1.
Ti(OCH(CH3)2)4 , CH4
OCH(CH3)2
OCH(CH3)2
Ti
OCH(CH3)2
(H3C)2 HCO
Ti
(H3C)2 HCO
OCH(CH3)2
O
O
OH
OH
N2 Purge
Ti(OCH(CH3)2)4
H2O
N2 Purge
ALD cycles
Repeat
H2O, CH4
OCH(CH3)2
OCH(CH3)2
Ti
OCH(CH3)2
(H3C)2 HCO
Ti
(H3C)2 HCO
OCH(CH3)2
O
O
OH
OH
Ti
OH
HO
Ti
HO
O
O
OH
N2 Purge
Precursor-1: Ti(OCH(CH3)2)4 		Precursor-2: H2O

## Slide 2
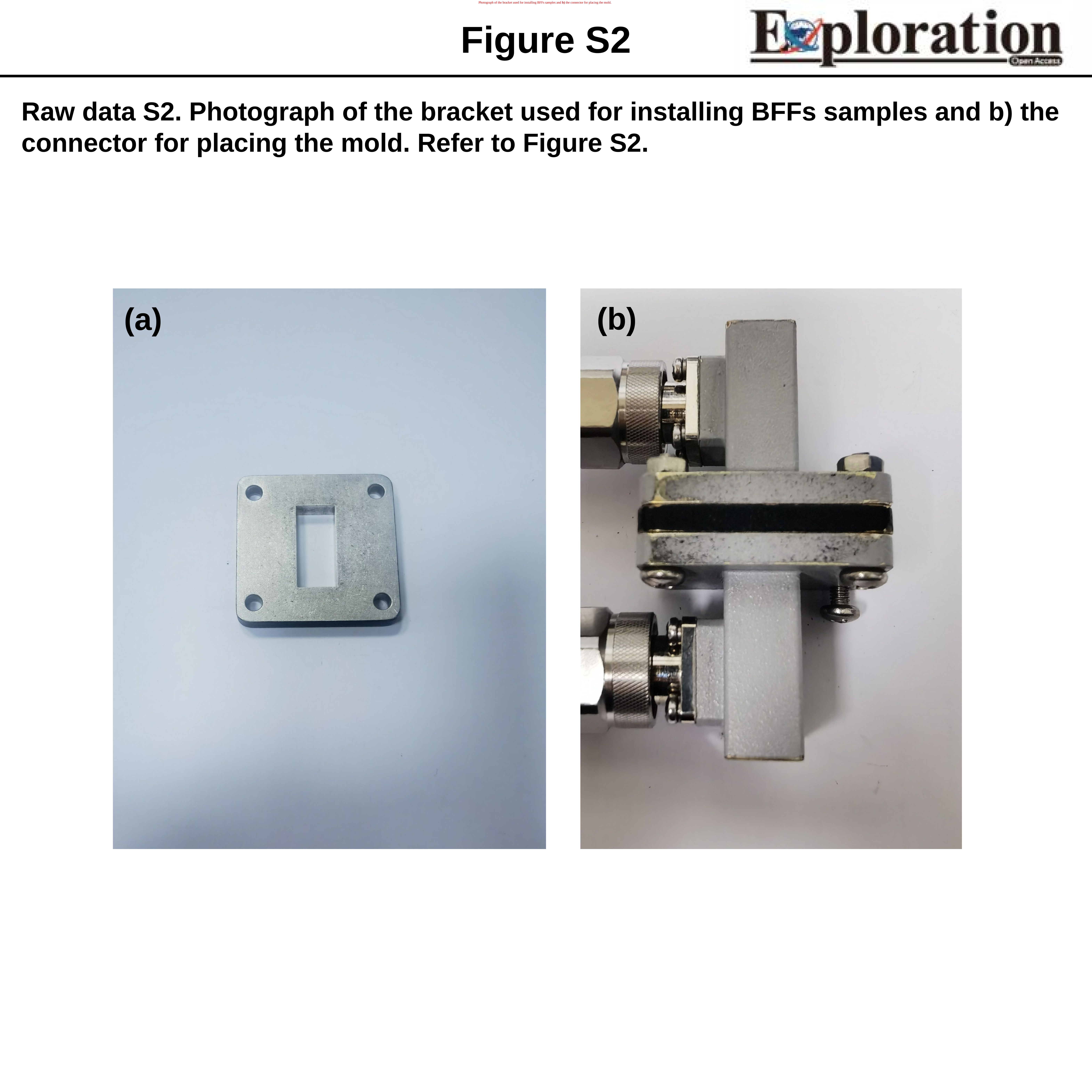

Photograph of the bracket used for installing BFFs samples and b) the connector for placing the mold.
Figure S2
Raw data S2. Photograph of the bracket used for installing BFFs samples and b) the connector for placing the mold. Refer to Figure S2.
(b)
(a)

## Slide 3
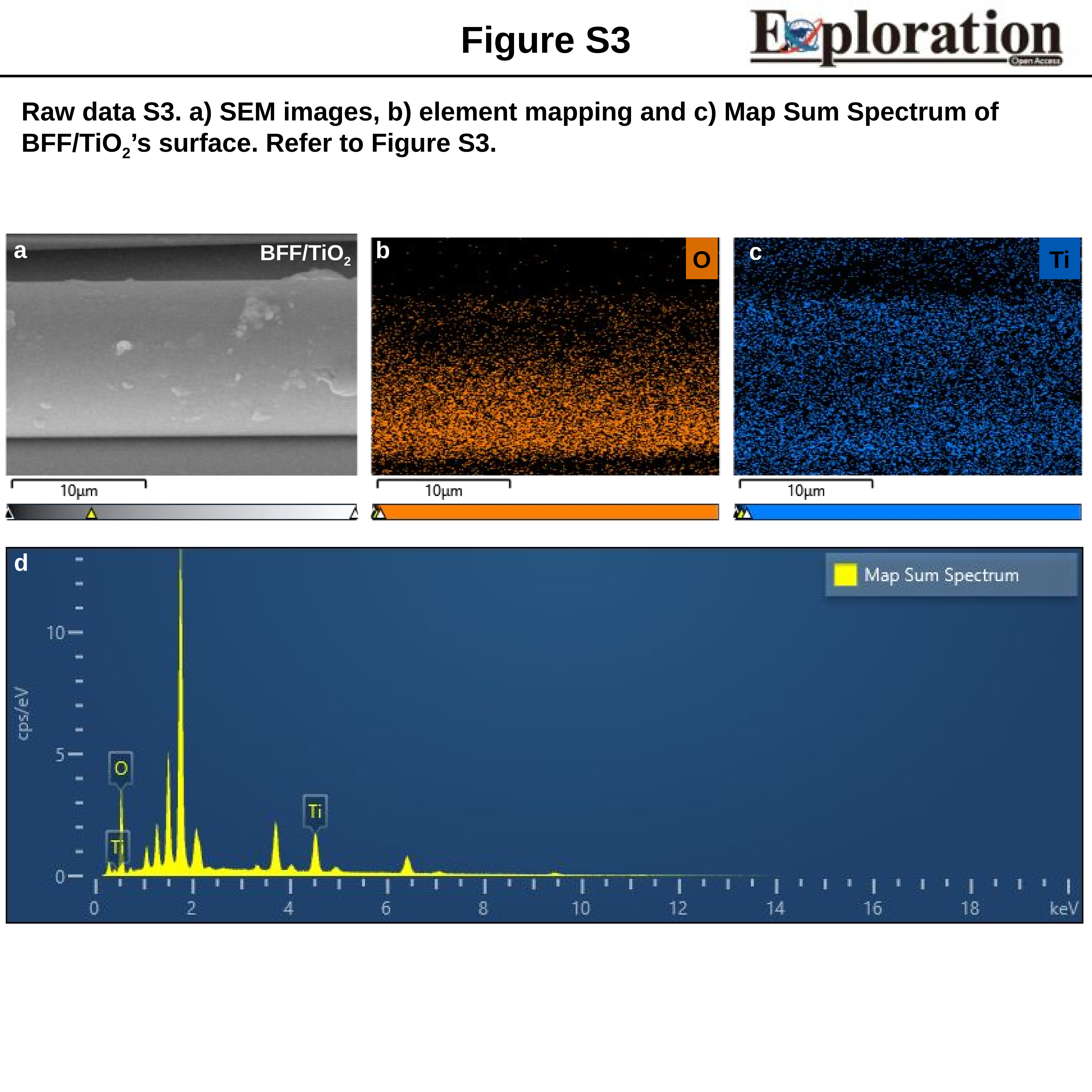

Figure S3
Raw data S3. a) SEM images, b) element mapping and c) Map Sum Spectrum of BFF/TiO2’s surface. Refer to Figure S3.
a
b
c
BFF/TiO2
O
Ti
d

## Slide 4
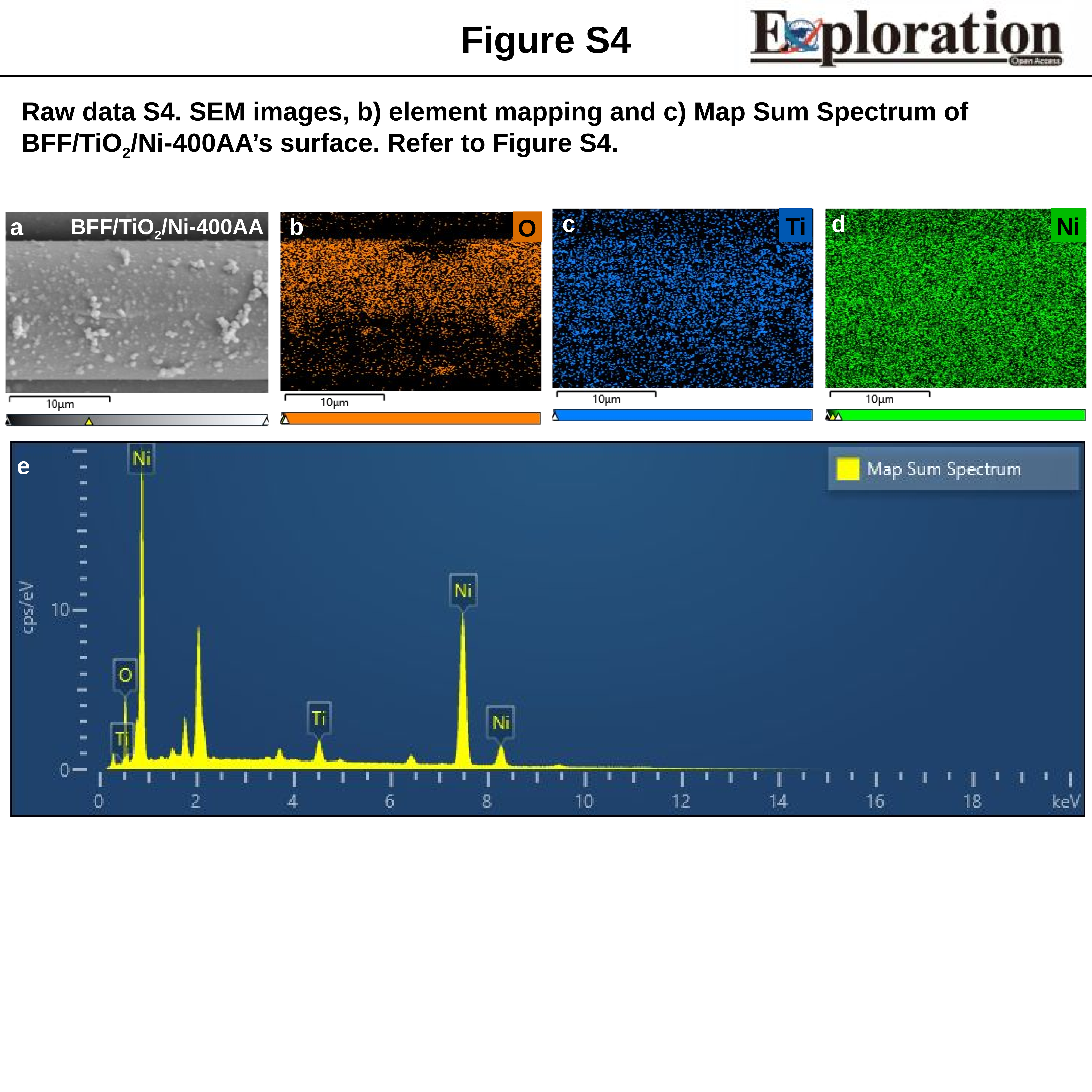

Figure S4
Raw data S4. SEM images, b) element mapping and c) Map Sum Spectrum of BFF/TiO2/Ni-400AA’s surface. Refer to Figure S4.
c
d
Ti
Ni
b
a
BFF/TiO2/Ni-400AA
O
e

## Slide 5
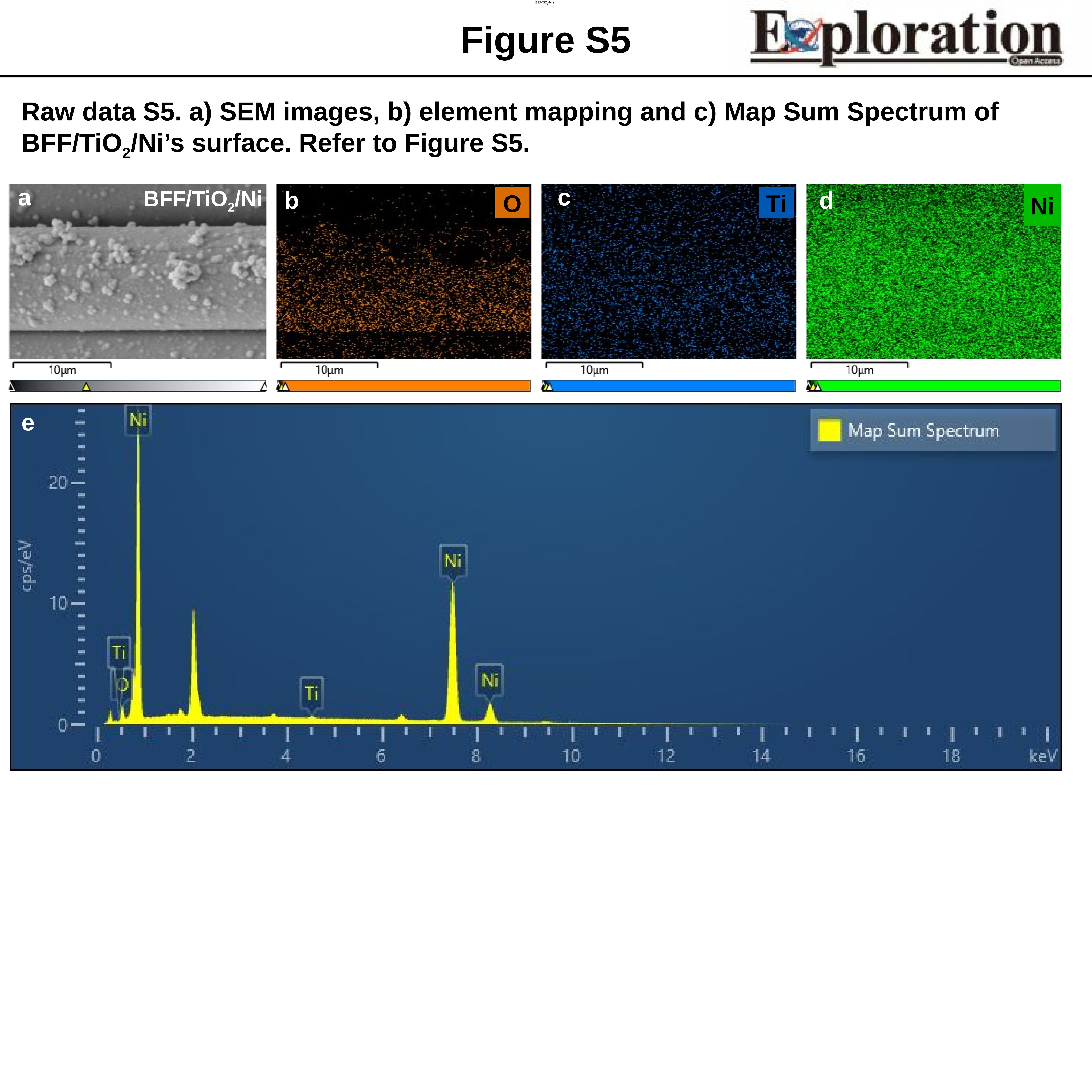

BFF/TiO2/Ni’s
Figure S5
Raw data S5. a) SEM images, b) element mapping and c) Map Sum Spectrum of BFF/TiO2/Ni’s surface. Refer to Figure S5.
a
c
BFF/TiO2/Ni
Ni
b
d
O
Ti
e

## Slide 6
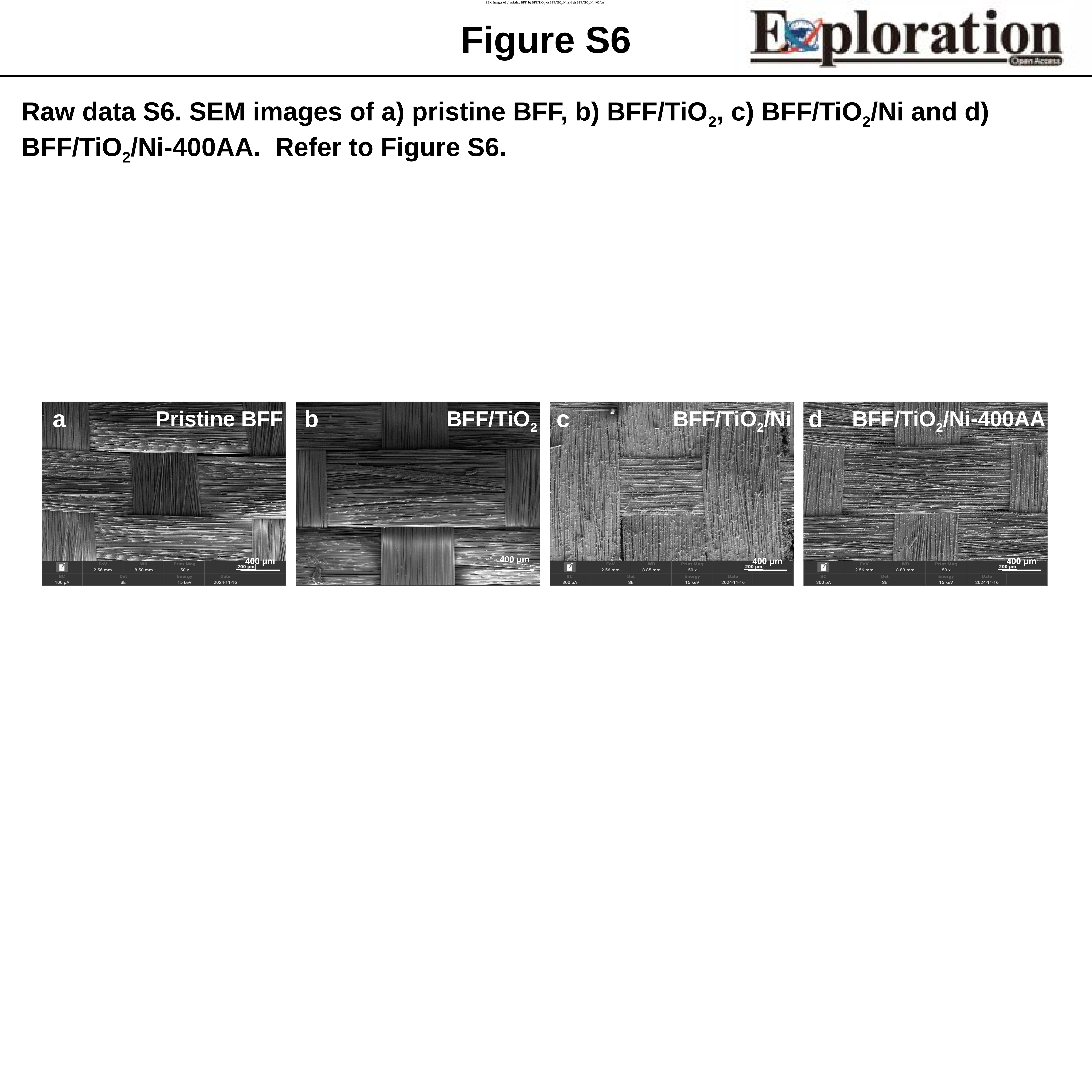

SEM images of a) pristine BFF, b) BFF/TiO2, c) BFF/TiO2/Ni and d) BFF/TiO2/Ni-400AA
Figure S6
Raw data S6. SEM images of a) pristine BFF, b) BFF/TiO2, c) BFF/TiO2/Ni and d) BFF/TiO2/Ni-400AA. Refer to Figure S6.
Pristine BFF
BFF/TiO2
BFF/TiO2/Ni
BFF/TiO2/Ni-400AA
400 μm
400 μm
400 μm
400 μm
a
b
c
d

## Slide 7
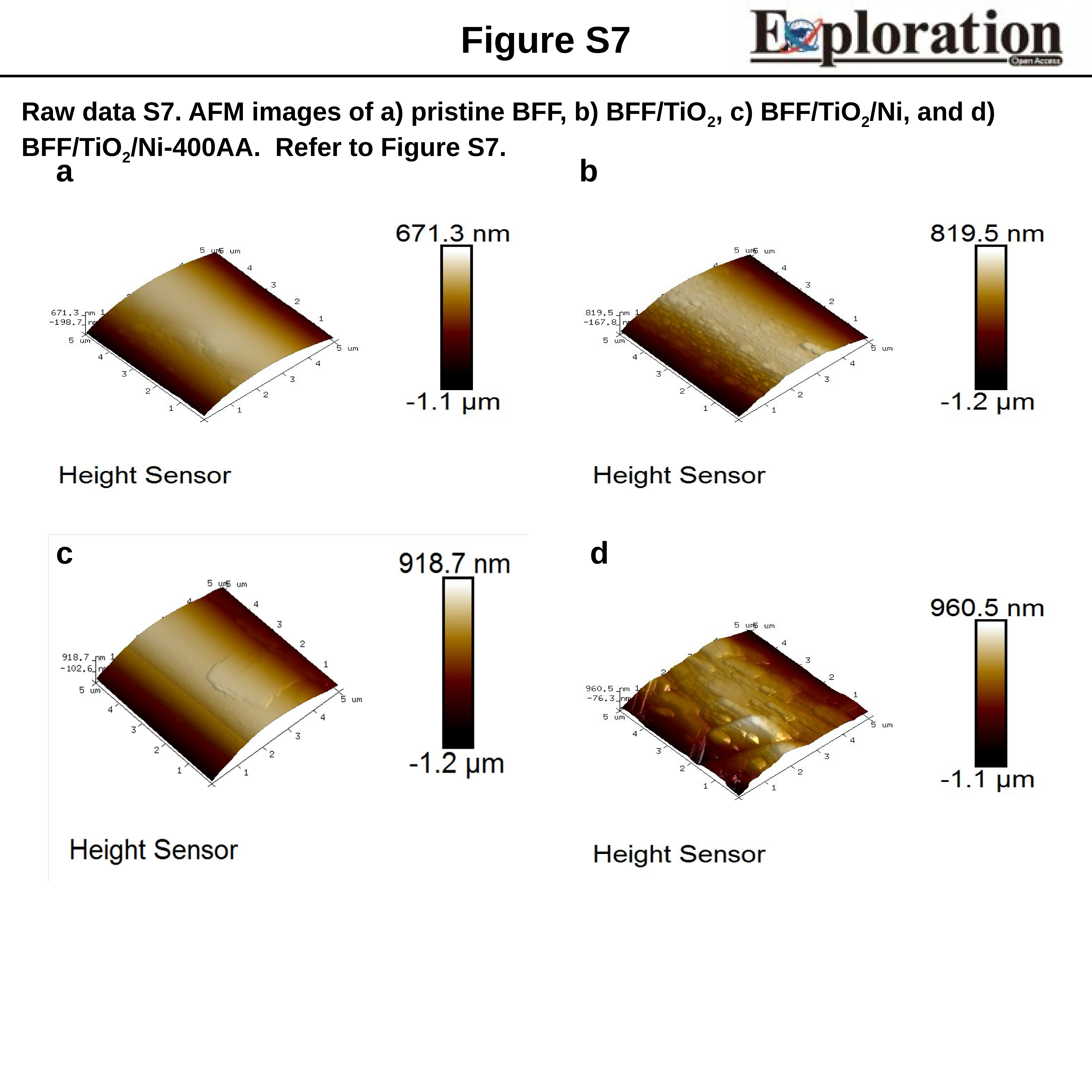

Figure S7
Raw data S7. AFM images of a) pristine BFF, b) BFF/TiO2, c) BFF/TiO2/Ni, and d) BFF/TiO2/Ni-400AA. Refer to Figure S7.
a
b
c
d

## Slide 8
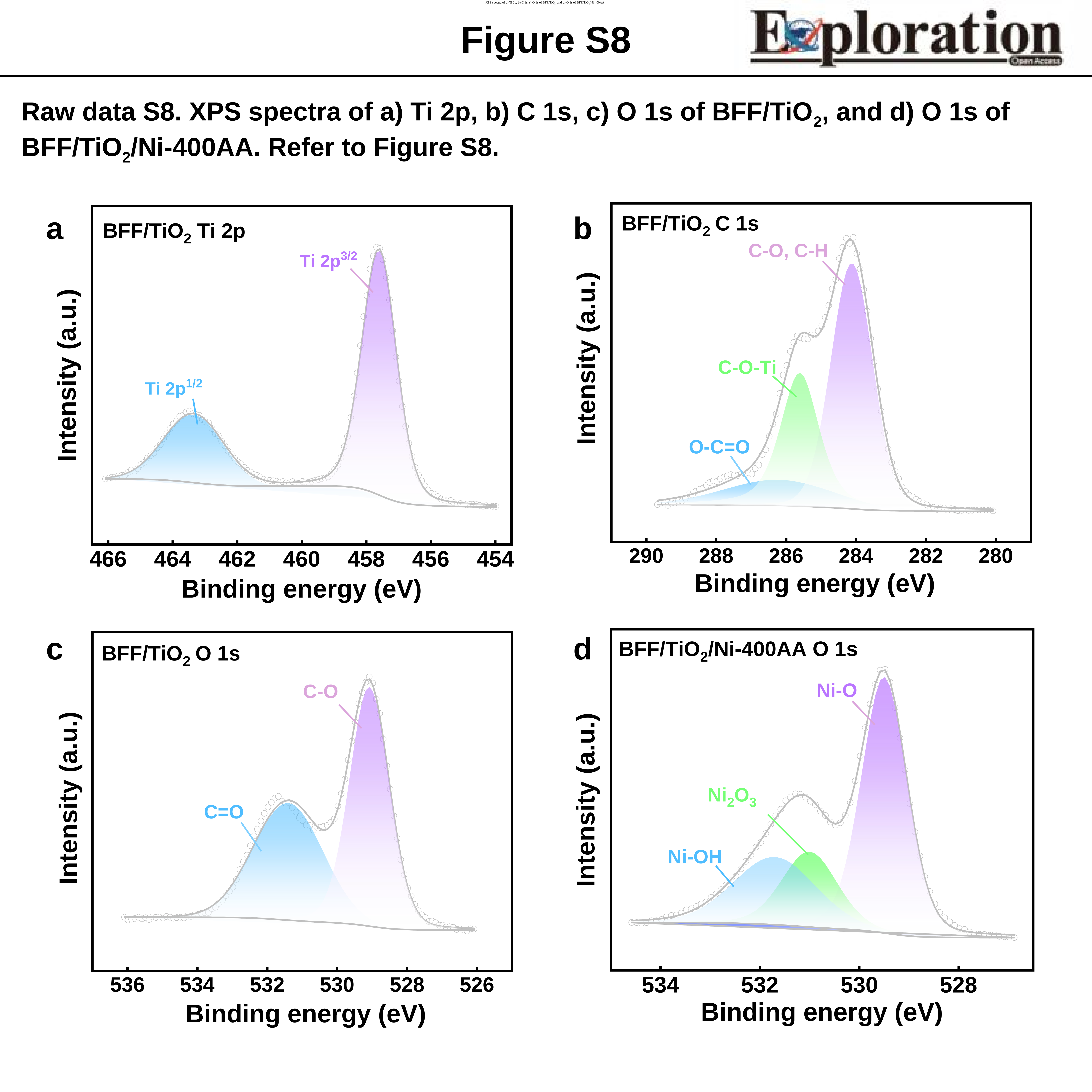

XPS spectra of a) Ti 2p, b) C 1s, c) O 1s of BFF/TiO2, and d) O 1s of BFF/TiO2/Ni-400AA
Figure S8
Raw data S8. XPS spectra of a) Ti 2p, b) C 1s, c) O 1s of BFF/TiO2, and d) O 1s of BFF/TiO2/Ni-400AA. Refer to Figure S8.
a
b
c
d

## Slide 9
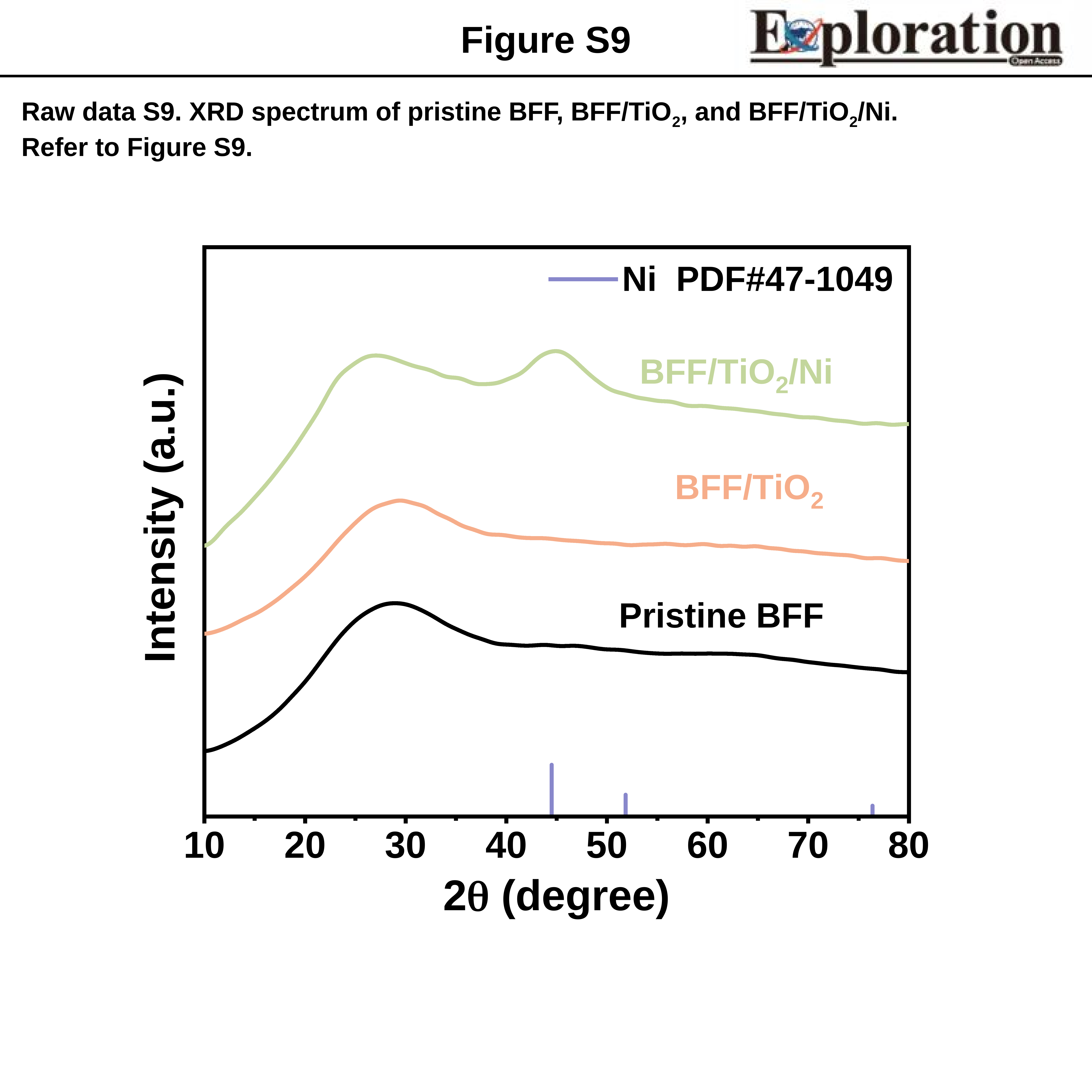

Figure S9
Raw data S9. XRD spectrum of pristine BFF, BFF/TiO2, and BFF/TiO2/Ni.
Refer to Figure S9.

## Slide 10
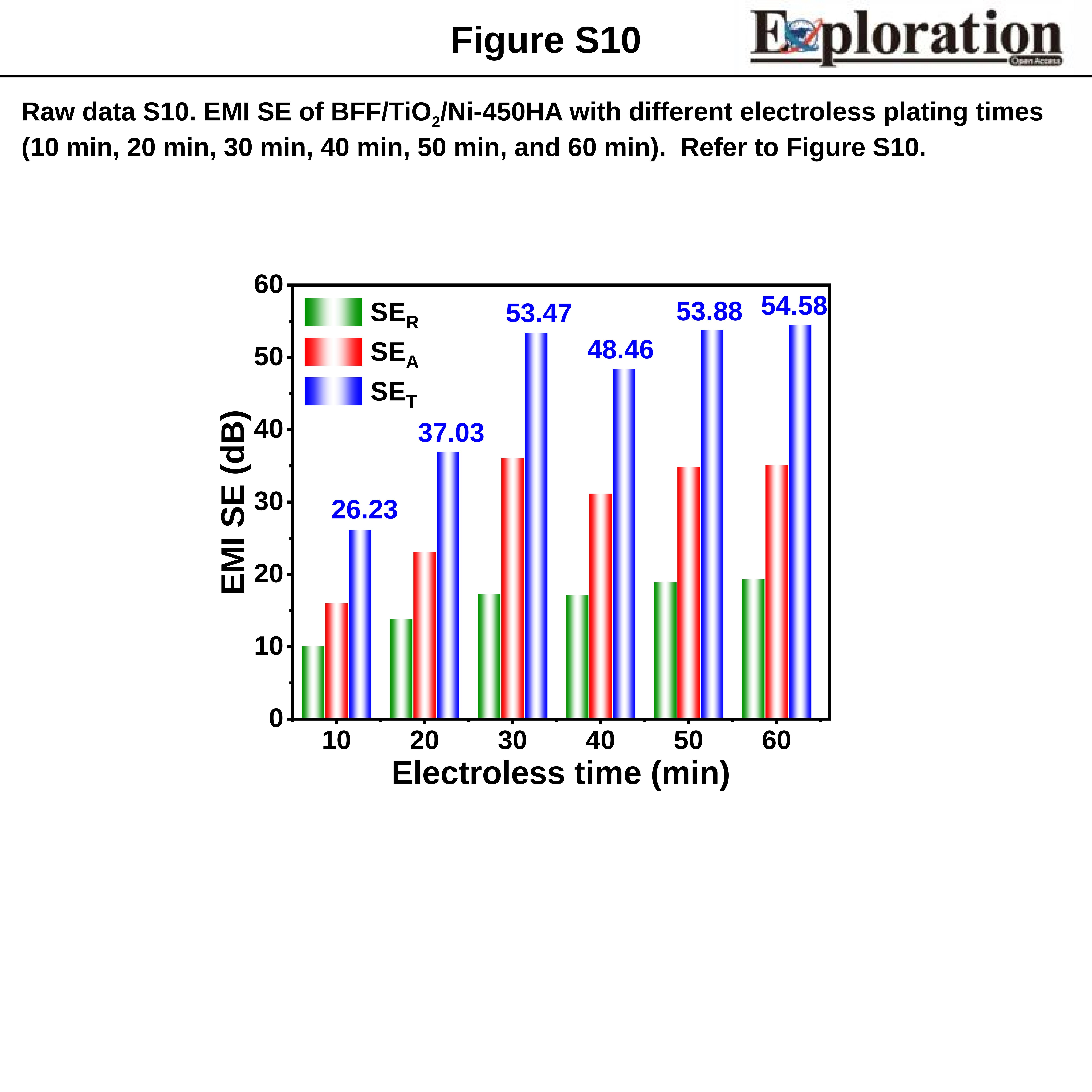

Figure S10
Raw data S10. EMI SE of BFF/TiO2/Ni-450HA with different electroless plating times (10 min, 20 min, 30 min, 40 min, 50 min, and 60 min). Refer to Figure S10.

## Slide 11
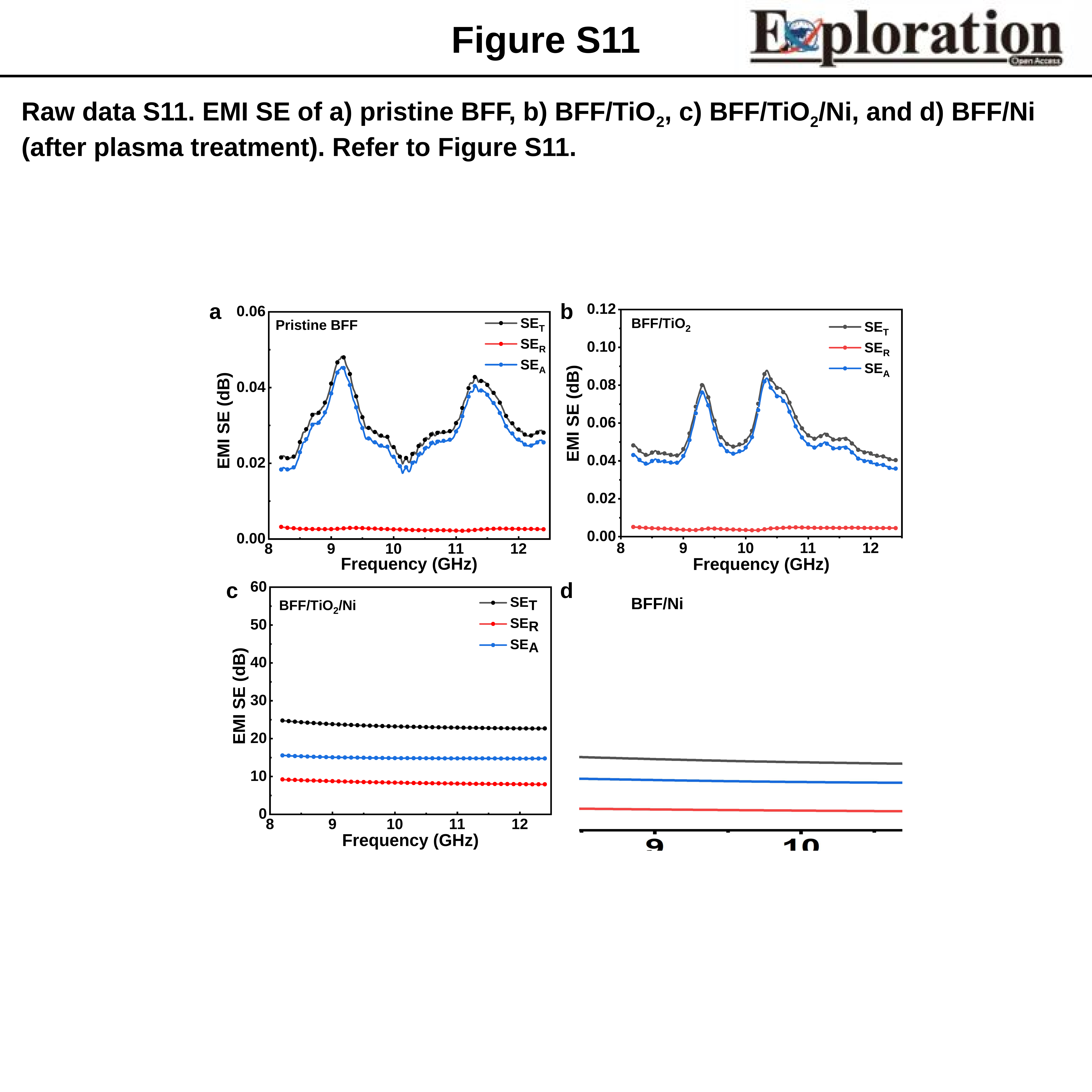

Figure S11
Raw data S11. EMI SE of a) pristine BFF, b) BFF/TiO2, c) BFF/TiO2/Ni, and d) BFF/Ni (after plasma treatment). Refer to Figure S11.
a
b
c
d
BFF/Ni

## Slide 12
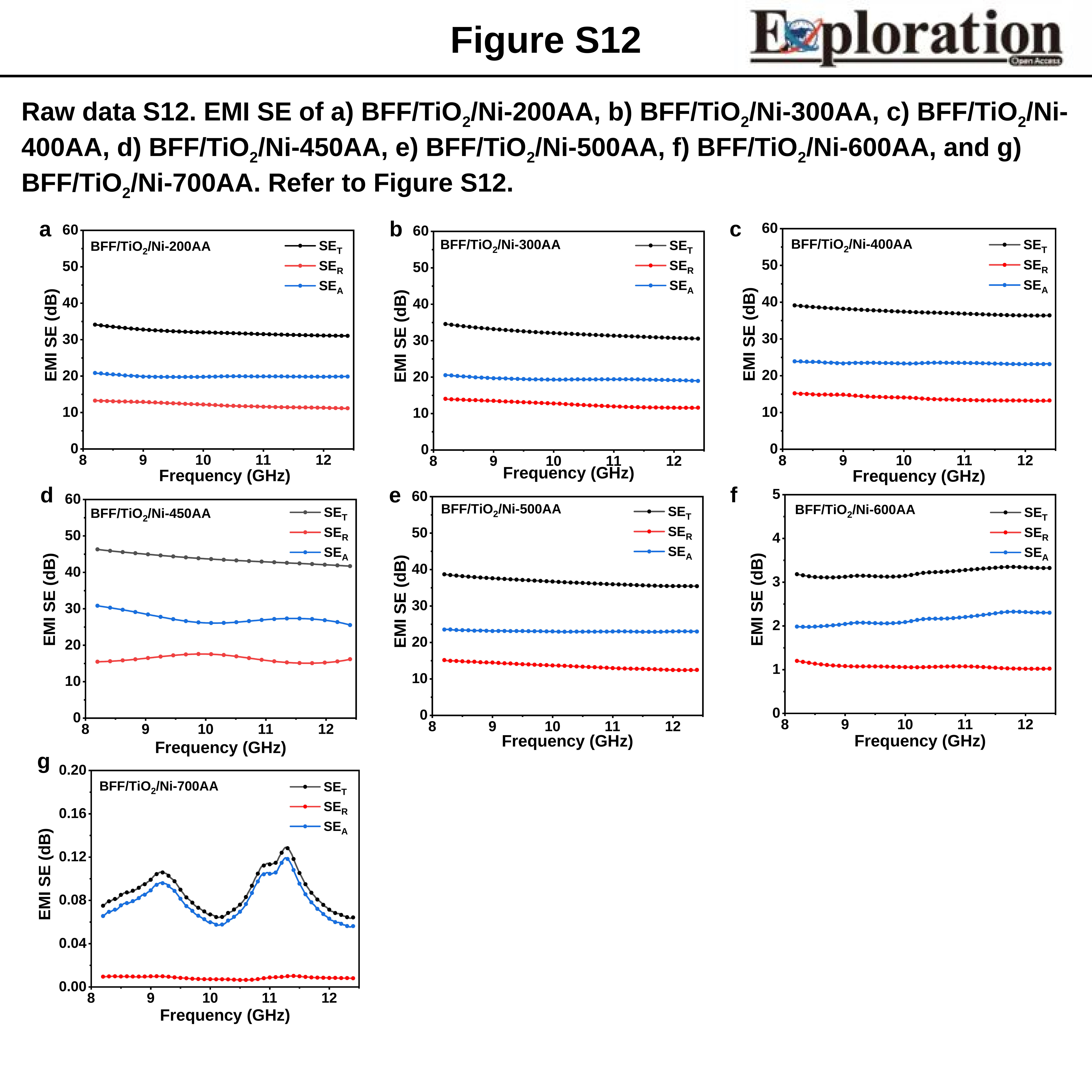

Figure S12
Raw data S12. EMI SE of a) BFF/TiO2/Ni-200AA, b) BFF/TiO2/Ni-300AA, c) BFF/TiO2/Ni-400AA, d) BFF/TiO2/Ni-450AA, e) BFF/TiO2/Ni-500AA, f) BFF/TiO2/Ni-600AA, and g) BFF/TiO2/Ni-700AA. Refer to Figure S12.
a
b
c
f
d
e
g

## Slide 13
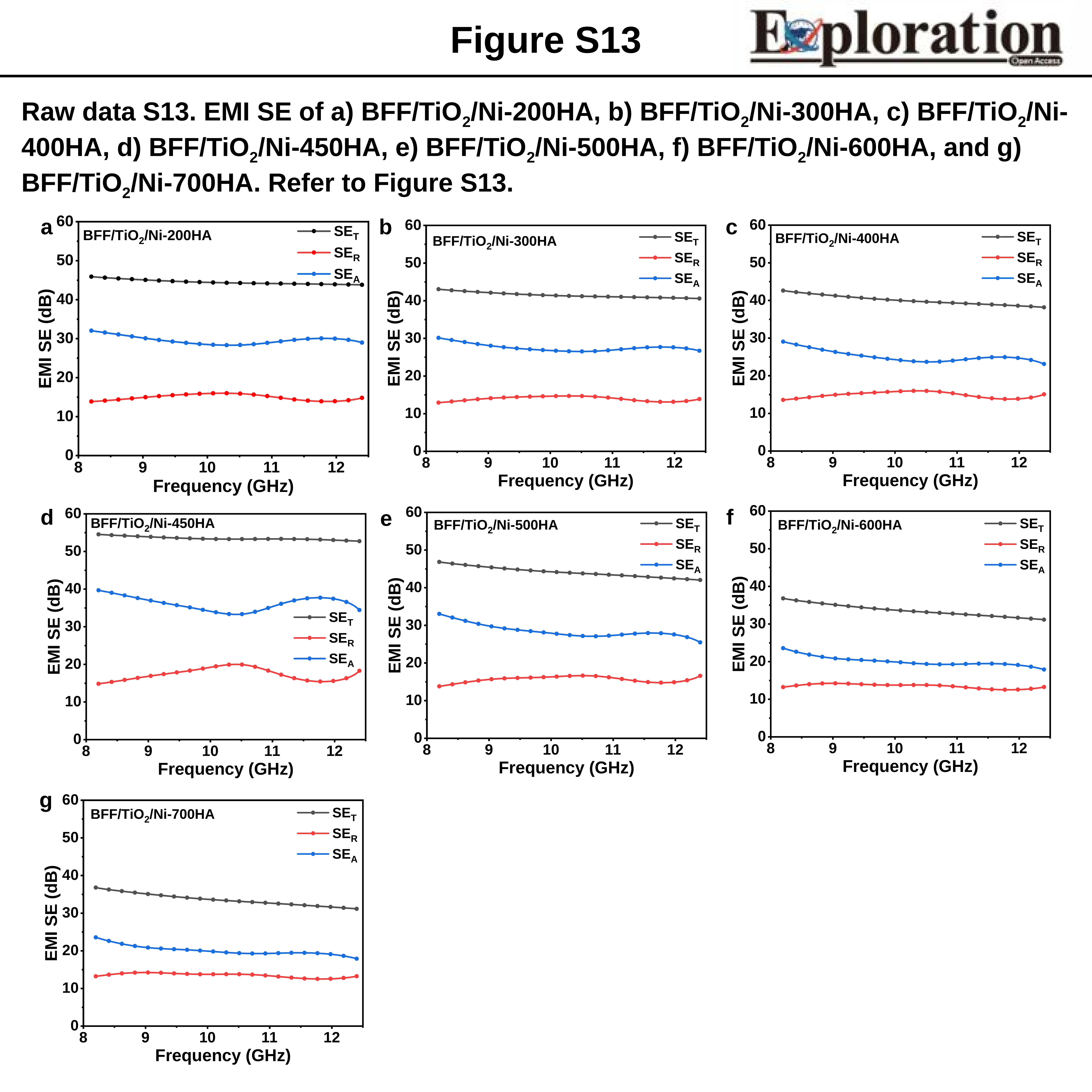

Figure S13
Raw data S13. EMI SE of a) BFF/TiO2/Ni-200HA, b) BFF/TiO2/Ni-300HA, c) BFF/TiO2/Ni-400HA, d) BFF/TiO2/Ni-450HA, e) BFF/TiO2/Ni-500HA, f) BFF/TiO2/Ni-600HA, and g) BFF/TiO2/Ni-700HA. Refer to Figure S13.
a
b
c
f
d
e
g

## Slide 14
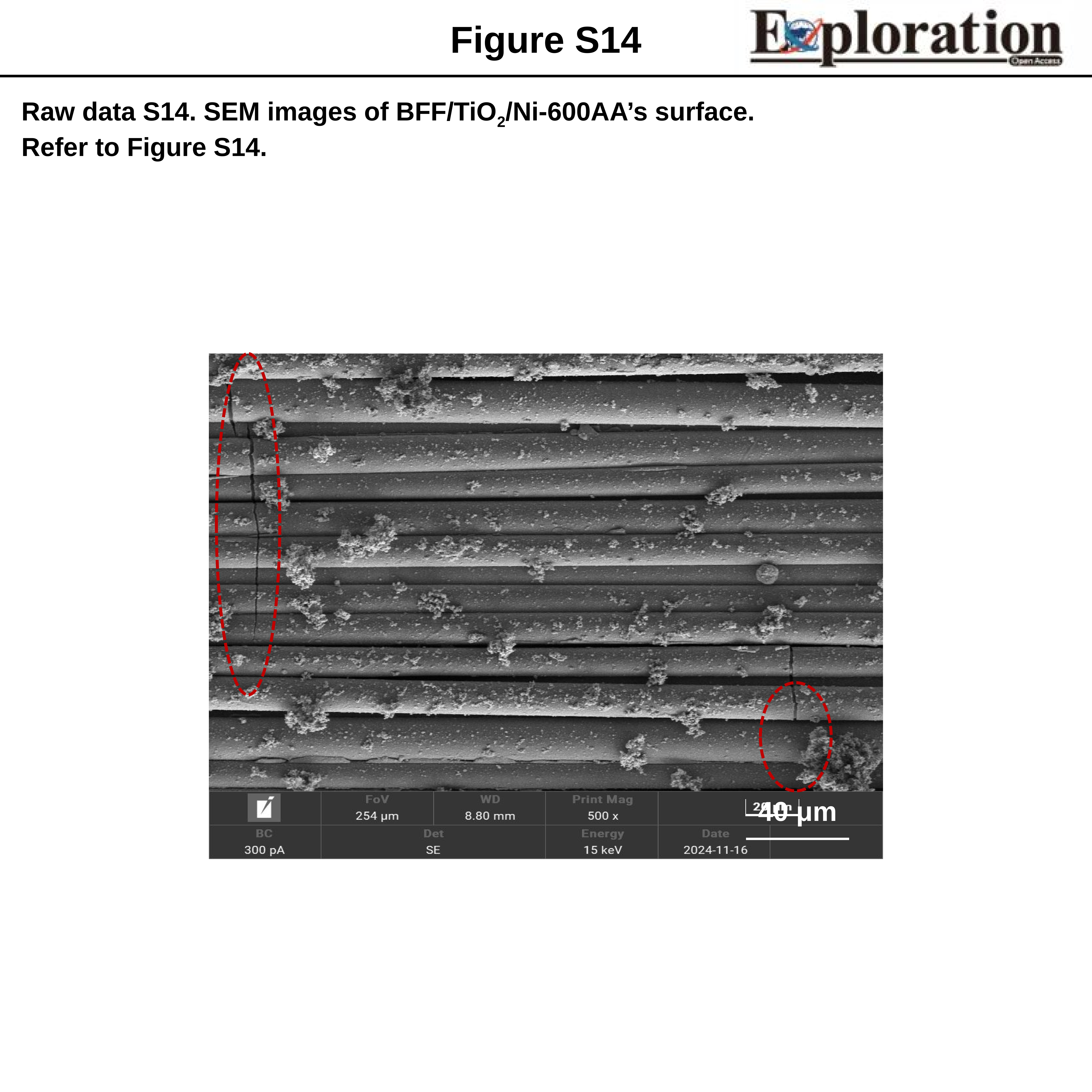

Figure S14
Raw data S14. SEM images of BFF/TiO2/Ni-600AA’s surface.
Refer to Figure S14.
40 μm

## Slide 15
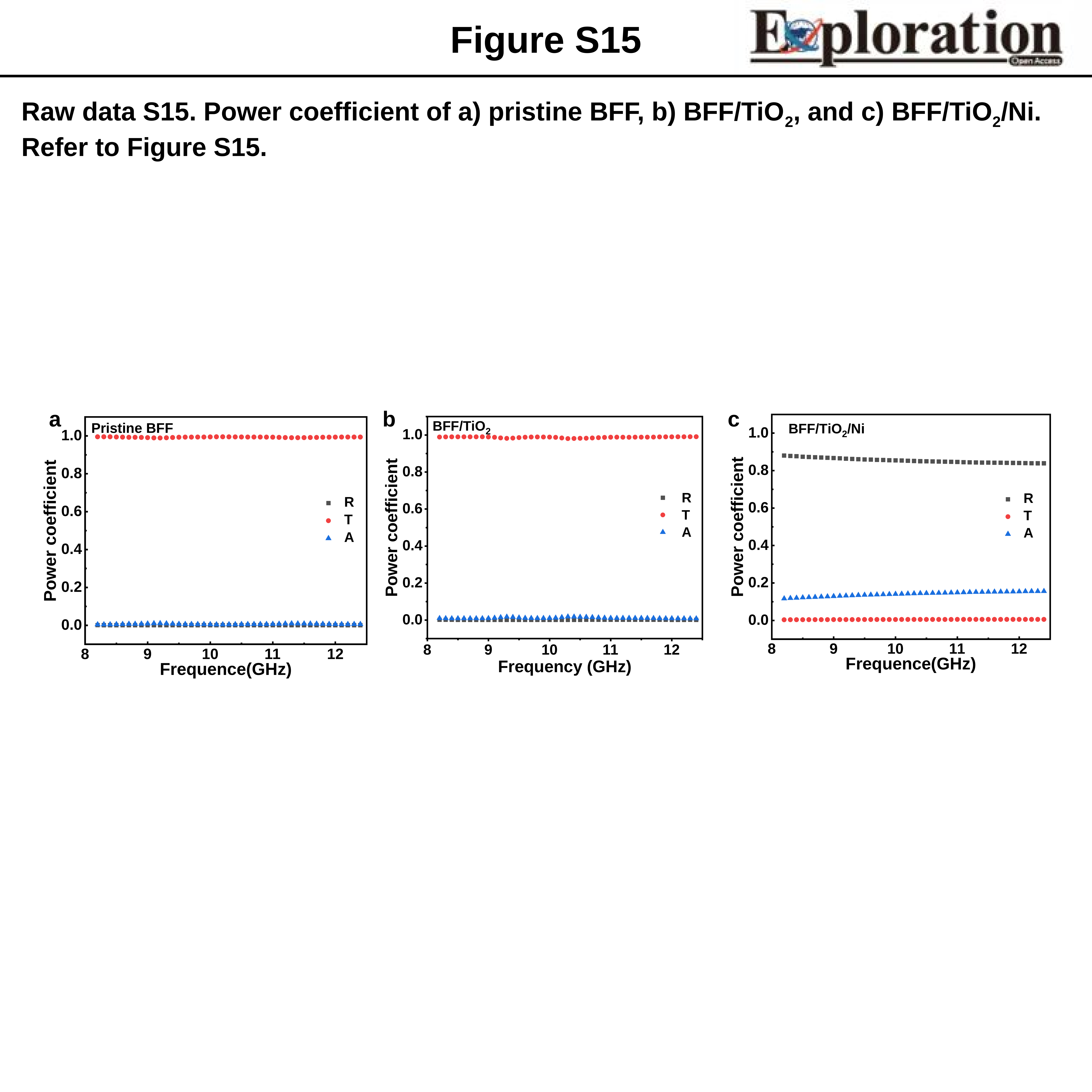

Figure S15
Raw data S15. Power coefficient of a) pristine BFF, b) BFF/TiO2, and c) BFF/TiO2/Ni.
Refer to Figure S15.
a
b
c

## Slide 16
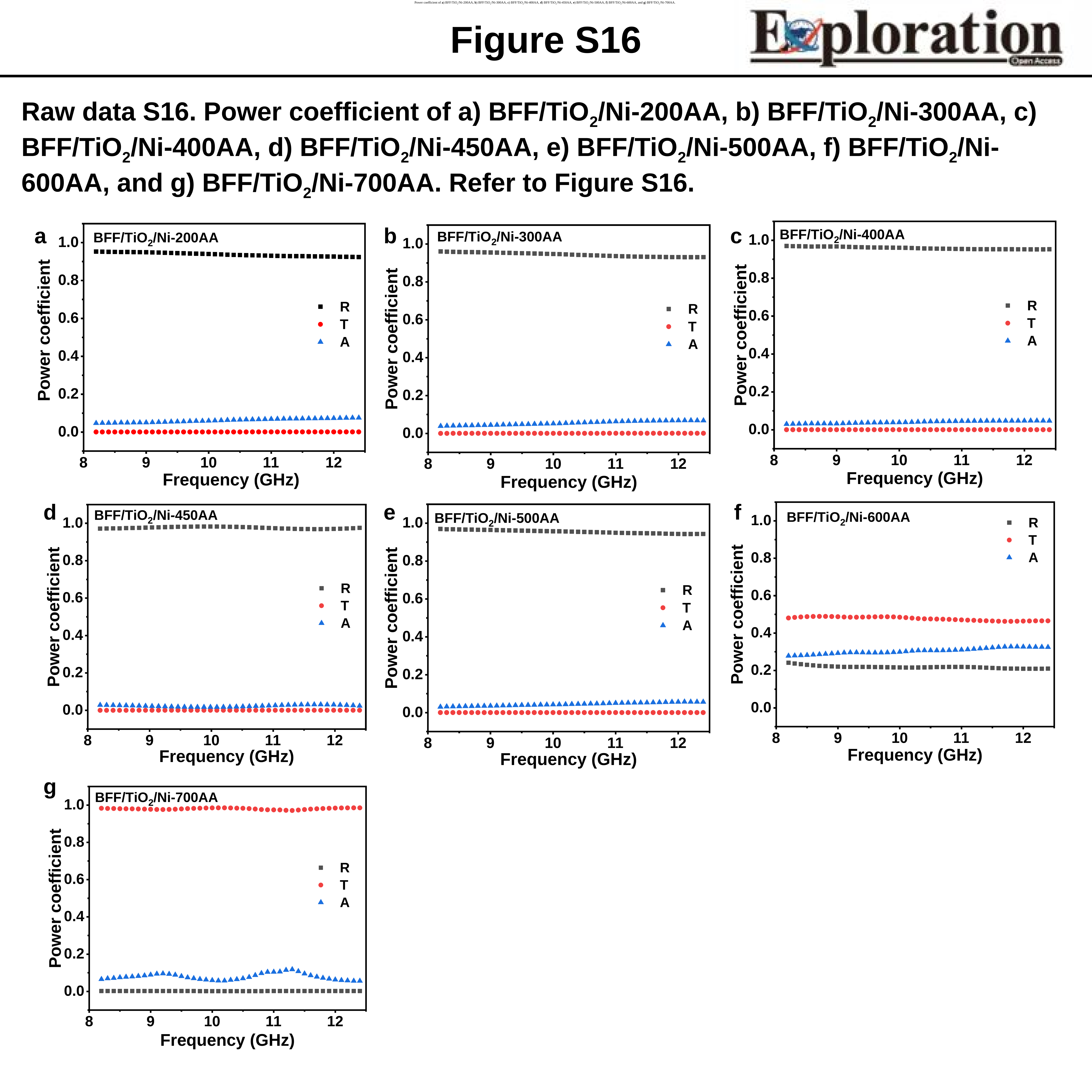

Power coefficient of a) BFF/TiO2/Ni-200AA, b) BFF/TiO2/Ni-300AA, c) BFF/TiO2/Ni-400AA, d) BFF/TiO2/Ni-450AA, e) BFF/TiO2/Ni-500AA, f) BFF/TiO2/Ni-600AA, and g) BFF/TiO2/Ni-700AA.
Figure S16
Raw data S16. Power coefficient of a) BFF/TiO2/Ni-200AA, b) BFF/TiO2/Ni-300AA, c) BFF/TiO2/Ni-400AA, d) BFF/TiO2/Ni-450AA, e) BFF/TiO2/Ni-500AA, f) BFF/TiO2/Ni-600AA, and g) BFF/TiO2/Ni-700AA. Refer to Figure S16.
a
b
c
d
e
f
g

## Slide 17
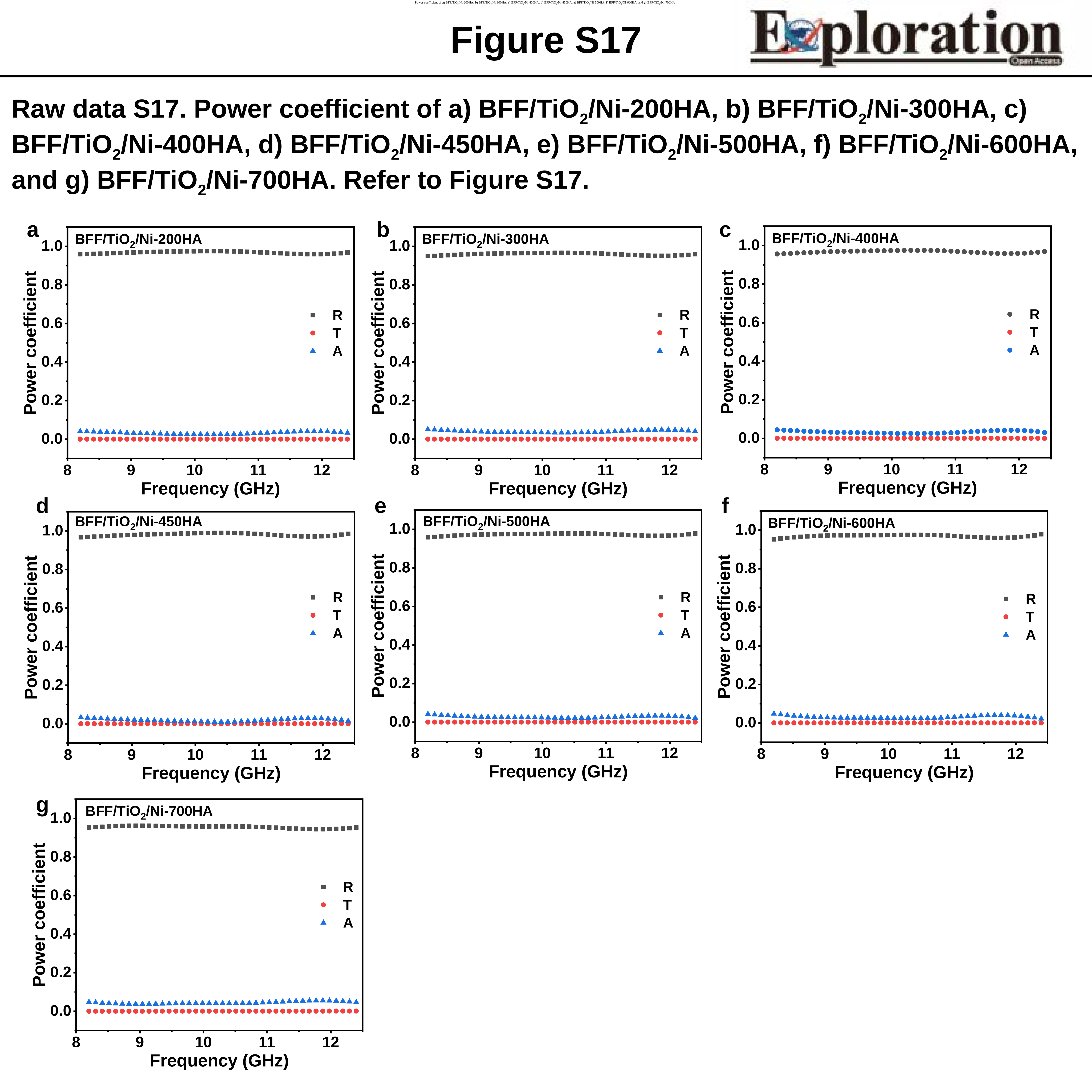

Power coefficient of a) BFF/TiO2/Ni-200HA, b) BFF/TiO2/Ni-300HA, c) BFF/TiO2/Ni-400HA, d) BFF/TiO2/Ni-450HA, e) BFF/TiO2/Ni-500HA, f) BFF/TiO2/Ni-600HA, and g) BFF/TiO2/Ni-700HA
Figure S17
Raw data S17. Power coefficient of a) BFF/TiO2/Ni-200HA, b) BFF/TiO2/Ni-300HA, c) BFF/TiO2/Ni-400HA, d) BFF/TiO2/Ni-450HA, e) BFF/TiO2/Ni-500HA, f) BFF/TiO2/Ni-600HA, and g) BFF/TiO2/Ni-700HA. Refer to Figure S17.
a
b
c
e
d
f
g

## Slide 18
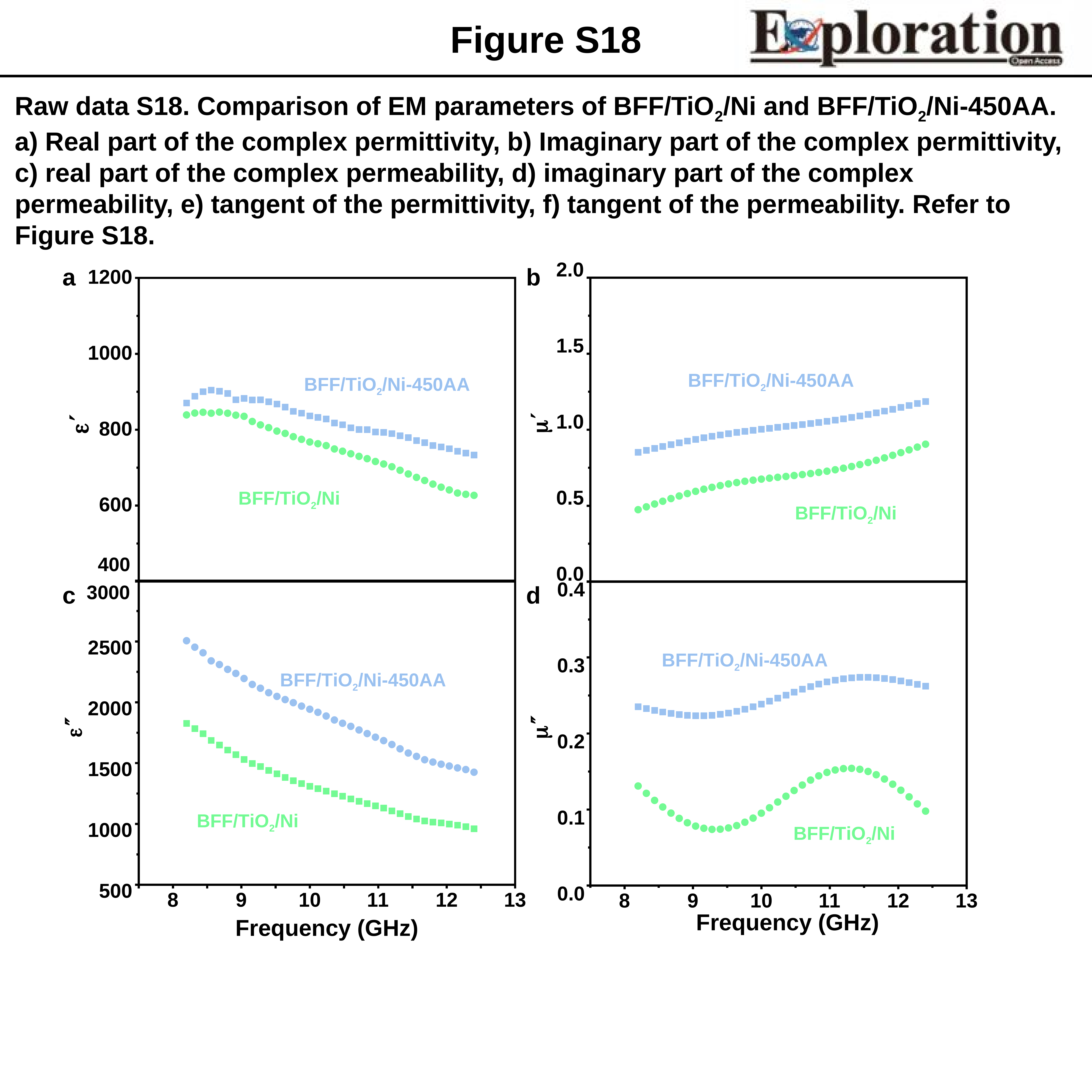

Figure S18
Raw data S18. Comparison of EM parameters of BFF/TiO2/Ni and BFF/TiO2/Ni-450AA. a) Real part of the complex permittivity, b) Imaginary part of the complex permittivity, c) real part of the complex permeability, d) imaginary part of the complex permeability, e) tangent of the permittivity, f) tangent of the permeability. Refer to Figure S18.
a
b
400
3000
c
d

## Slide 19
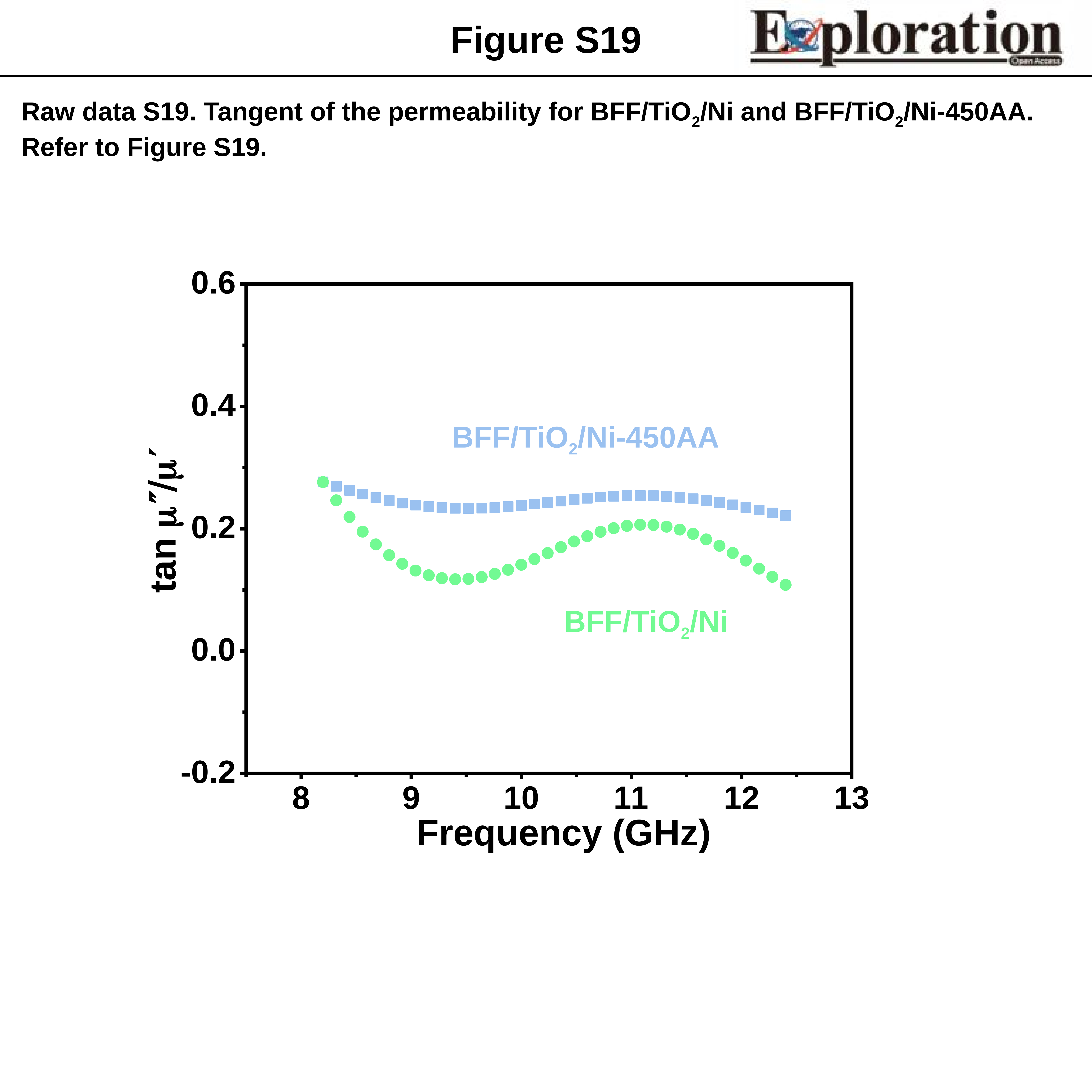

Figure S19
Raw data S19. Tangent of the permeability for BFF/TiO2/Ni and BFF/TiO2/Ni-450AA.
Refer to Figure S19.

## Slide 20
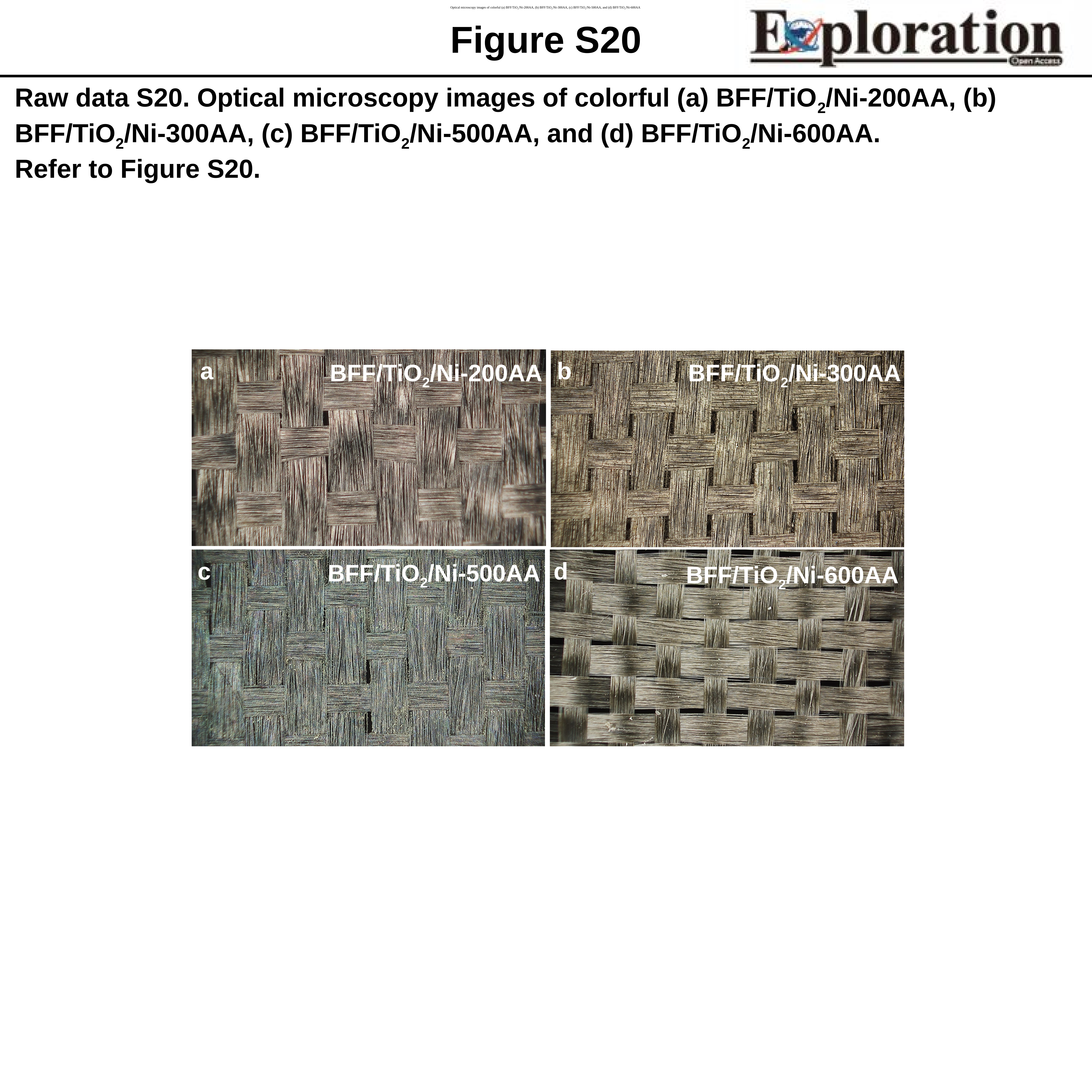

Optical microscopy images of colorful (a) BFF/TiO2/Ni-200AA, (b) BFF/TiO2/Ni-300AA, (c) BFF/TiO2/Ni-500AA, and (d) BFF/TiO2/Ni-600AA
Figure S20
Raw data S20. Optical microscopy images of colorful (a) BFF/TiO2/Ni-200AA, (b) BFF/TiO2/Ni-300AA, (c) BFF/TiO2/Ni-500AA, and (d) BFF/TiO2/Ni-600AA.
Refer to Figure S20.
a
BFF/TiO2/Ni-200AA
b
BFF/TiO2/Ni-300AA
BFF/TiO2/Ni-500AA
c
d
BFF/TiO2/Ni-600AA

## Slide 21
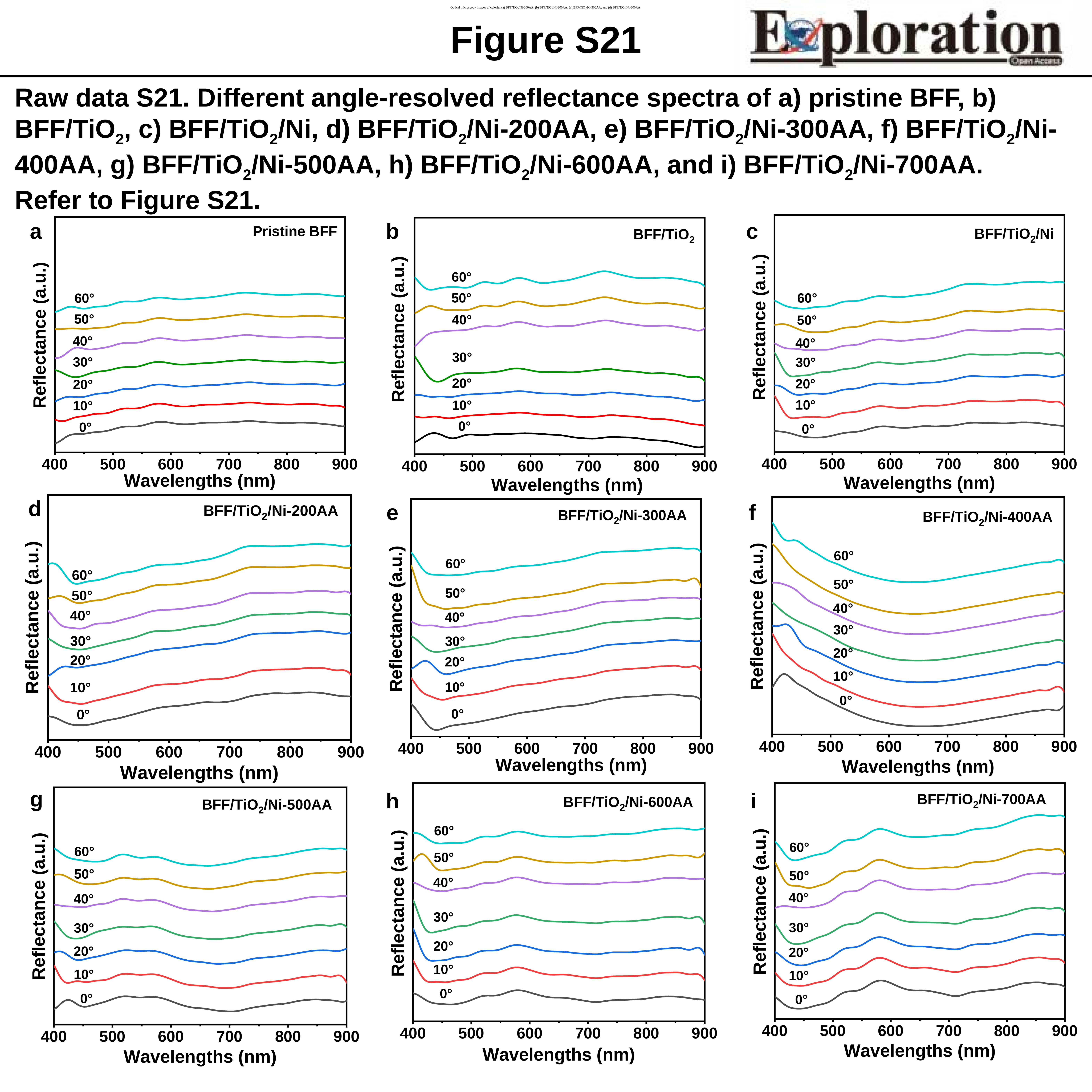

Optical microscopy images of colorful (a) BFF/TiO2/Ni-200AA, (b) BFF/TiO2/Ni-300AA, (c) BFF/TiO2/Ni-500AA, and (d) BFF/TiO2/Ni-600AA
Figure S21
Raw data S21. Different angle-resolved reflectance spectra of a) pristine BFF, b) BFF/TiO2, c) BFF/TiO2/Ni, d) BFF/TiO2/Ni-200AA, e) BFF/TiO2/Ni-300AA, f) BFF/TiO2/Ni-400AA, g) BFF/TiO2/Ni-500AA, h) BFF/TiO2/Ni-600AA, and i) BFF/TiO2/Ni-700AA.
Refer to Figure S21.
c
a
b
d
f
e
g
i
h

## Slide 22
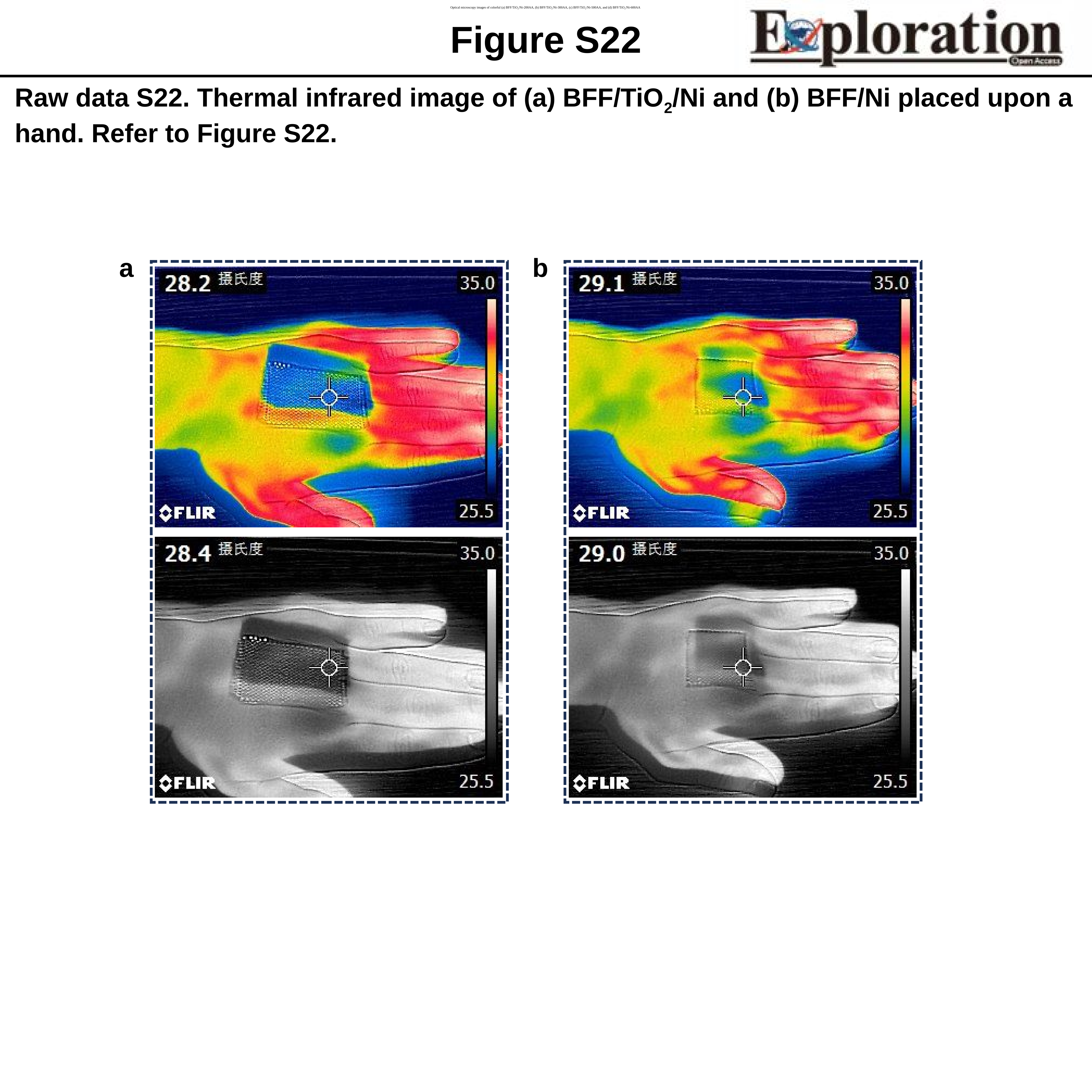

Optical microscopy images of colorful (a) BFF/TiO2/Ni-200AA, (b) BFF/TiO2/Ni-300AA, (c) BFF/TiO2/Ni-500AA, and (d) BFF/TiO2/Ni-600AA
Figure S22
Raw data S22. Thermal infrared image of (a) BFF/TiO2/Ni and (b) BFF/Ni placed upon a hand. Refer to Figure S22.
a
b
